# Supplementary figures and images for: CKAP5 stabilizes CENP-E at kinetochores by regulating microtubule-chromosome attachments (part 1 of 2)
Source: EMBO Rep. 2024 Feb 29;25(4):1909–35. doi: 10.1038/s44319-024-00106-9 (PMC11014917; doi:10.1038/s44319-024-00106-9)

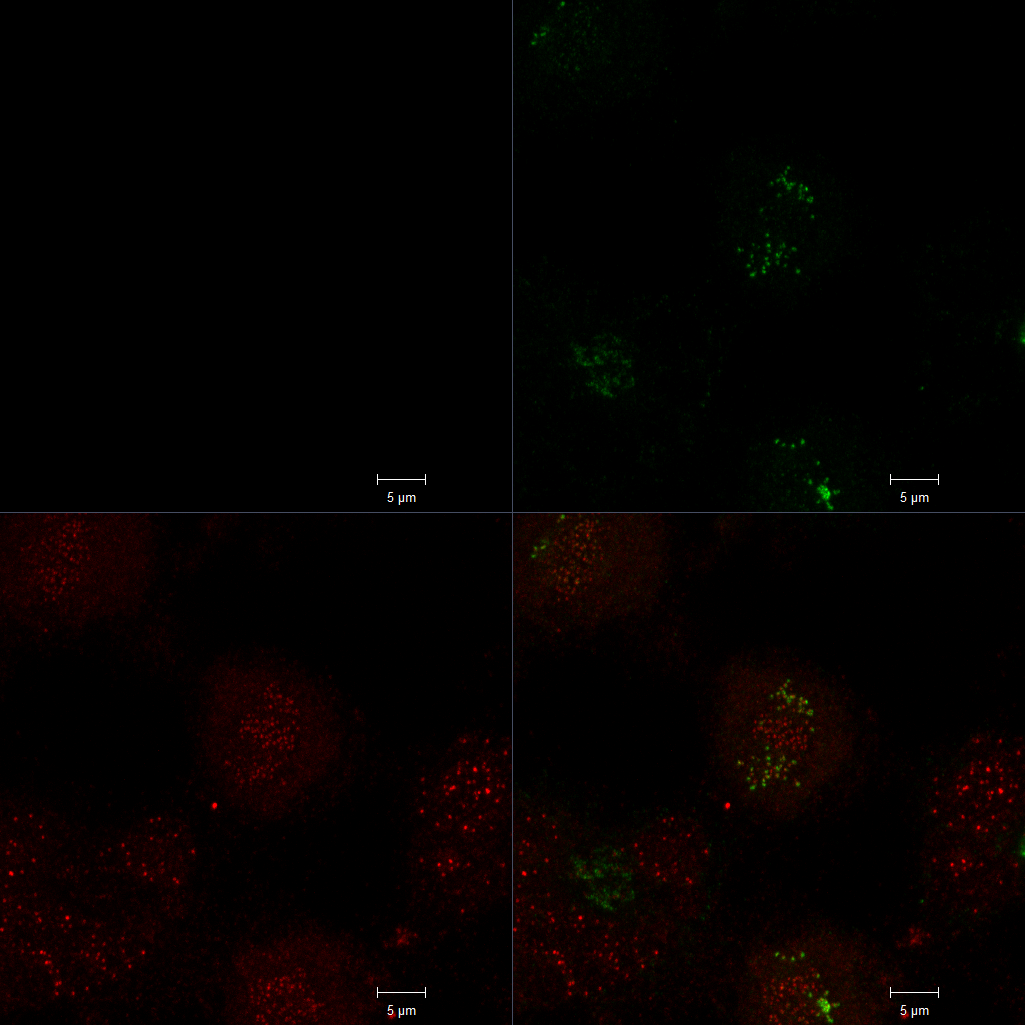

Supplement: Supplementary file 4 — Source Data Fig. 1 [file 44319_2024_106_MOESM4_ESM.zip › Figure 1/1A/CKAP5 esiRNA/CENPT647_CENPE488_DAPI_2018_11_12__12_38_34_Maximum intensity projection.tif]

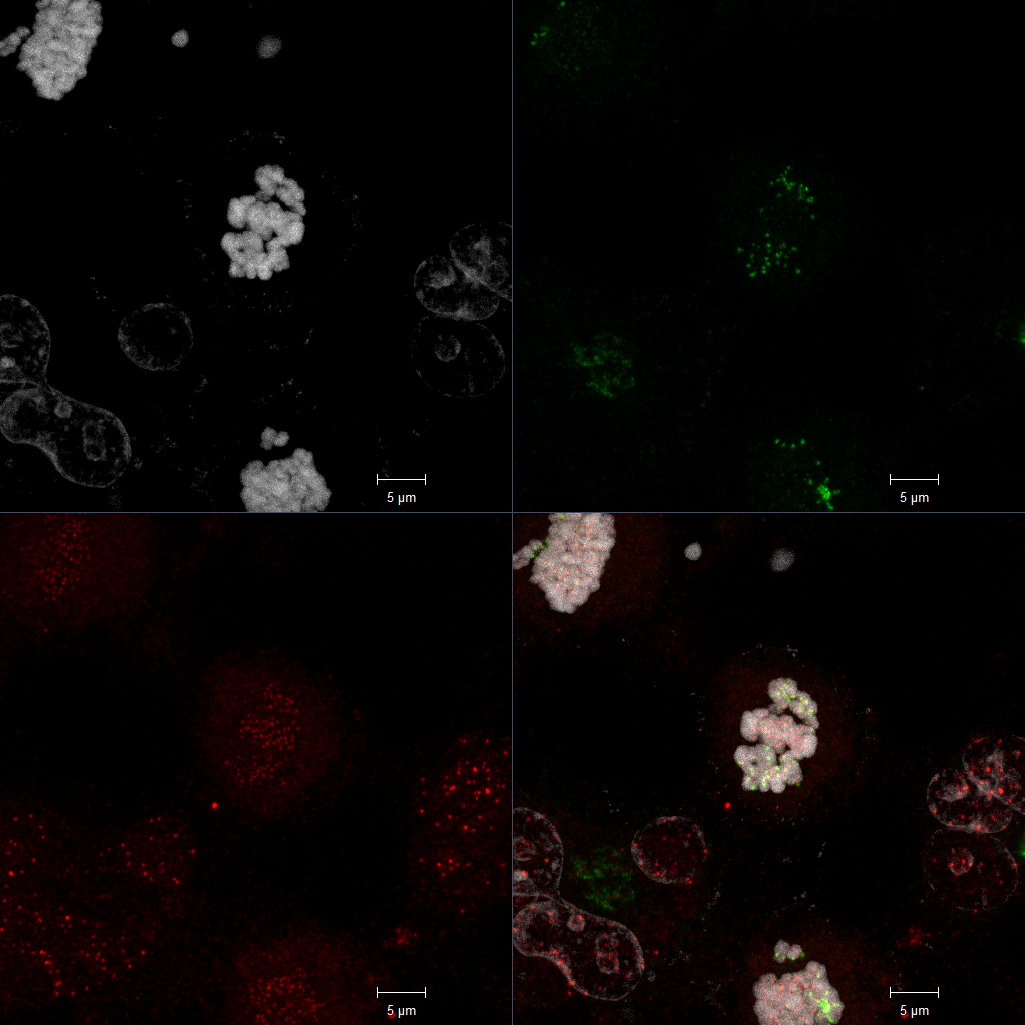

Supplement: Supplementary file 4 — Source Data Fig. 1 [file 44319_2024_106_MOESM4_ESM.zip › Figure 1/1A/CKAP5 esiRNA/CENPT647_CENPE488_DAPI_2018_11_12__12_38_34_Maximum intensity projection.tif1.tif]

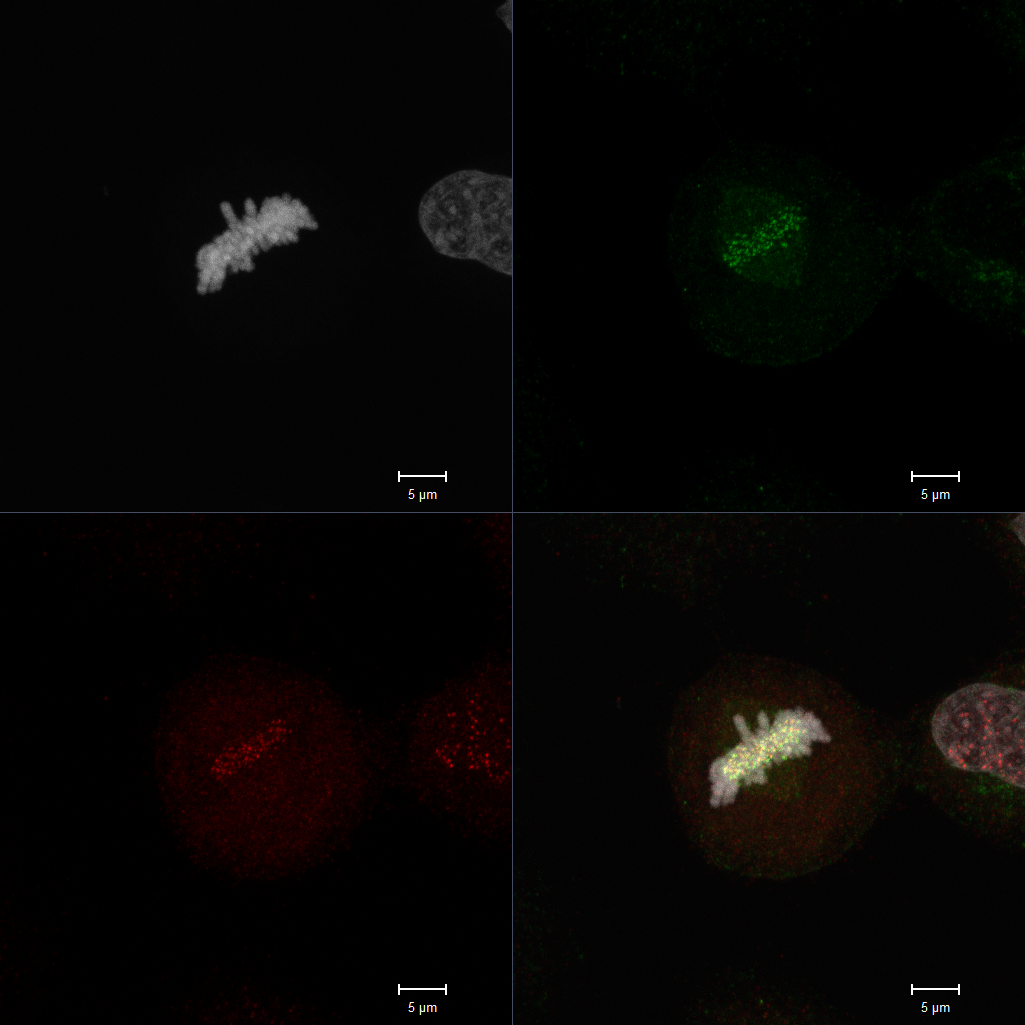

Supplement: Supplementary file 4 — Source Data Fig. 1 [file 44319_2024_106_MOESM4_ESM.zip › Figure 1/1A/control esiRNA/META _CENPT647_CENPE488_DAPI_2018_11_12__11_46_34_Maximum intensity projection.tif]

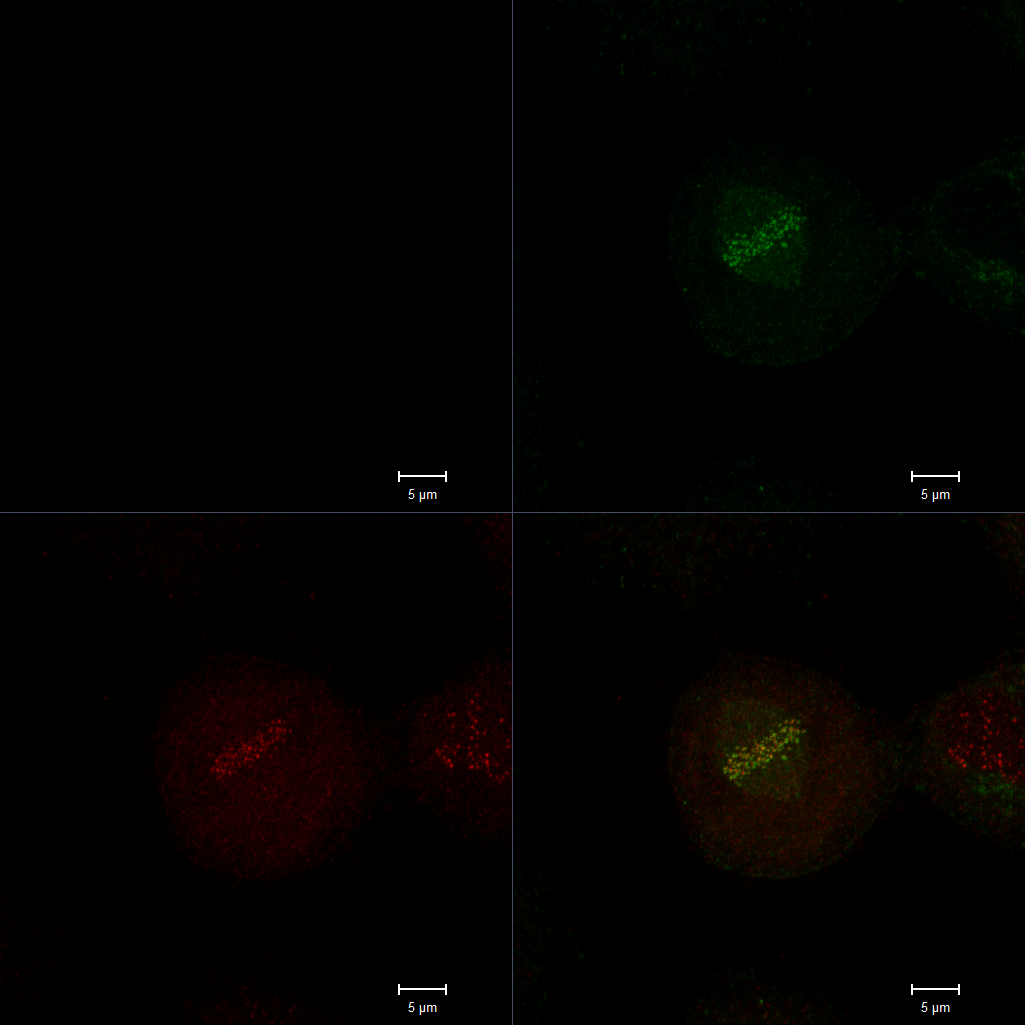

Supplement: Supplementary file 4 — Source Data Fig. 1 [file 44319_2024_106_MOESM4_ESM.zip › Figure 1/1A/control esiRNA/META _CENPT647_CENPE488_DAPI_2018_11_12__11_46_34_Maximum intensity projection.tif1.tif]

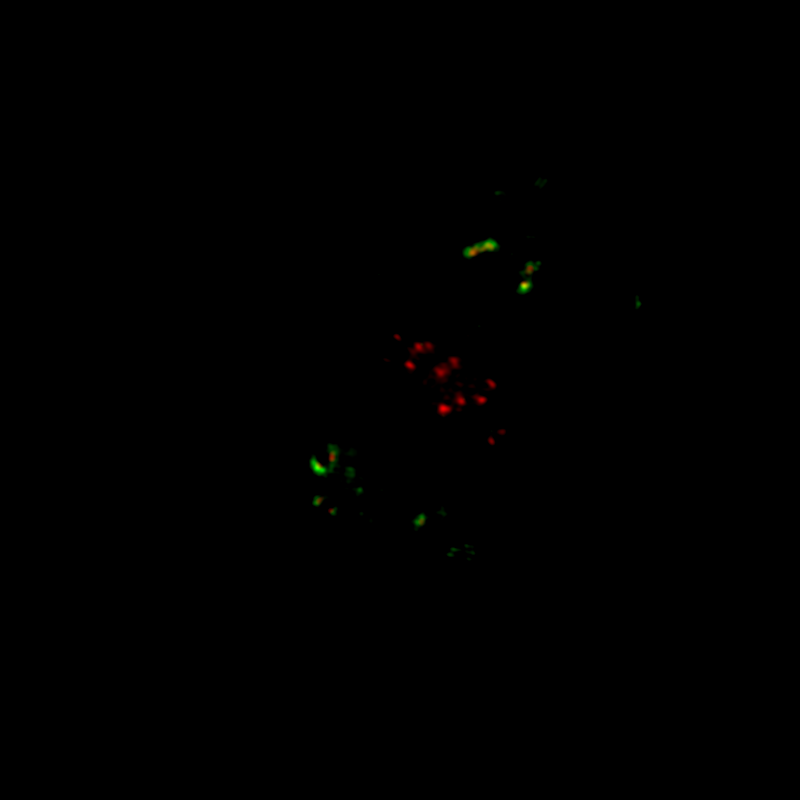

Supplement: Supplementary file 4 — Source Data Fig. 1 [file 44319_2024_106_MOESM4_ESM.zip › Figure 1/1B/CKAP5 esiRNA/Image 5_cut_Structured Illumination.tif]

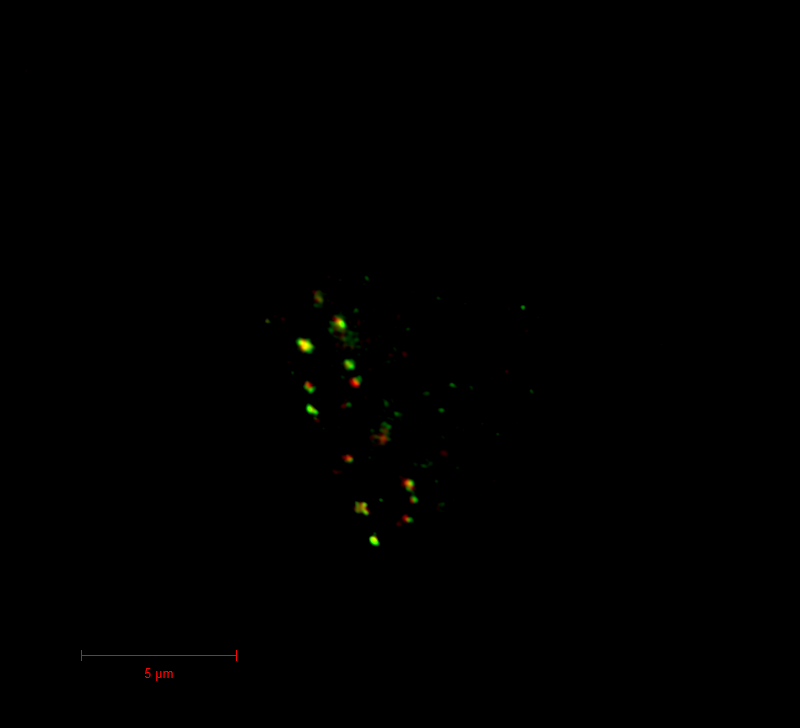

Supplement: Supplementary file 4 — Source Data Fig. 1 [file 44319_2024_106_MOESM4_ESM.zip › Figure 1/1B/Control esiRNA/Image 4_cut3_Structured Illumination.tif3.tif]

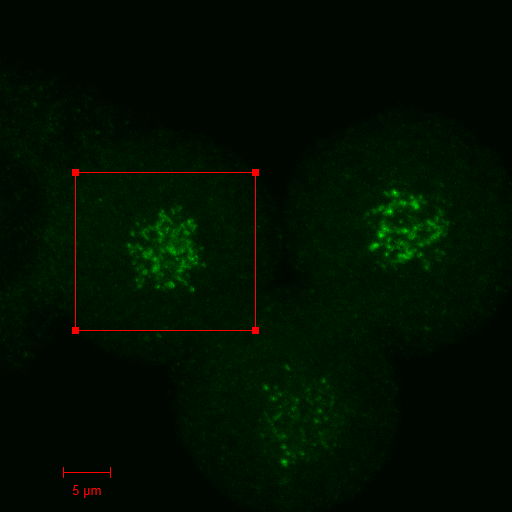

Supplement: Supplementary file 4 — Source Data Fig. 1 [file 44319_2024_106_MOESM4_ESM.zip › Figure 1/1D/CKAP5 esiRNA/Annotation_CENPE488_CENPT568_DAPI_290120_2020_01_29__13_13_40_Maximum intensity projectiontif1tif2tif3tif4.tif]

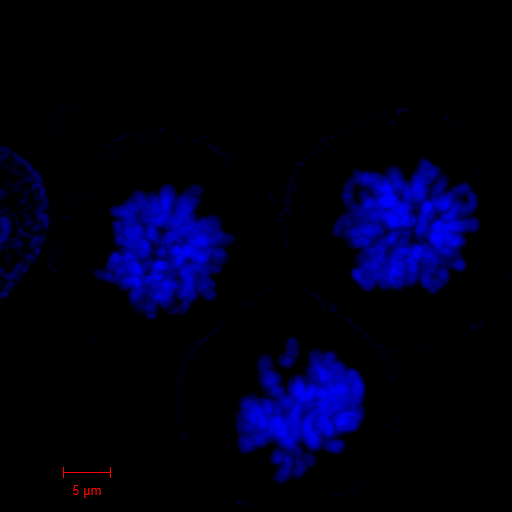

Supplement: Supplementary file 4 — Source Data Fig. 1 [file 44319_2024_106_MOESM4_ESM.zip › Figure 1/1D/CKAP5 esiRNA/CENPE488_CENPT568_DAPI_290120_2020_01_29__13_13_40_Maximum intensity projection.tif1.tif]

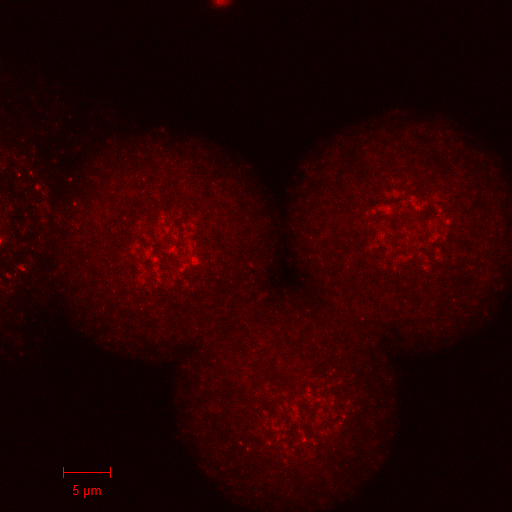

Supplement: Supplementary file 4 — Source Data Fig. 1 [file 44319_2024_106_MOESM4_ESM.zip › Figure 1/1D/CKAP5 esiRNA/CENPE488_CENPT568_DAPI_290120_2020_01_29__13_13_40_Maximum intensity projectiontif1.tif2.tif3.tif]

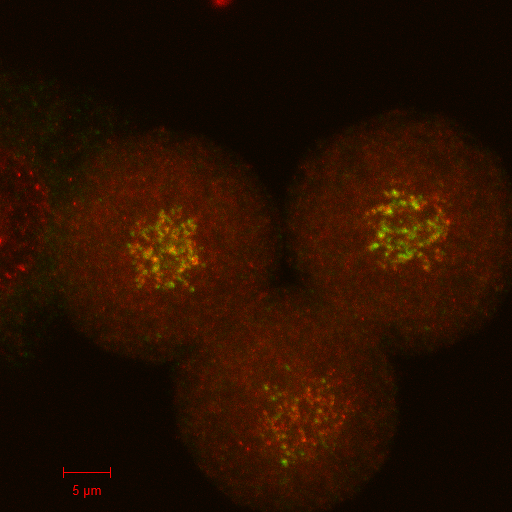

Supplement: Supplementary file 4 — Source Data Fig. 1 [file 44319_2024_106_MOESM4_ESM.zip › Figure 1/1D/CKAP5 esiRNA/CENPE488_CENPT568_DAPI_290120_2020_01_29__13_13_40_Maximum intensity projectiontif1tif2.tif3.tif4.tif]

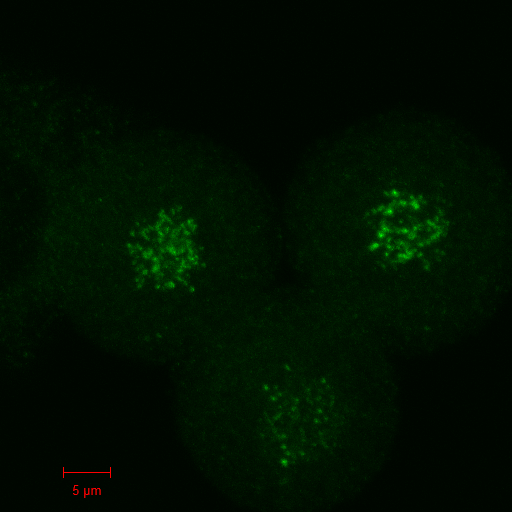

Supplement: Supplementary file 4 — Source Data Fig. 1 [file 44319_2024_106_MOESM4_ESM.zip › Figure 1/1D/CKAP5 esiRNA/CENPE488_CENPT568_DAPI_290120_2020_01_29__13_13_40_Maximum intensity projectiontif1tif2tif3tif4.tif]

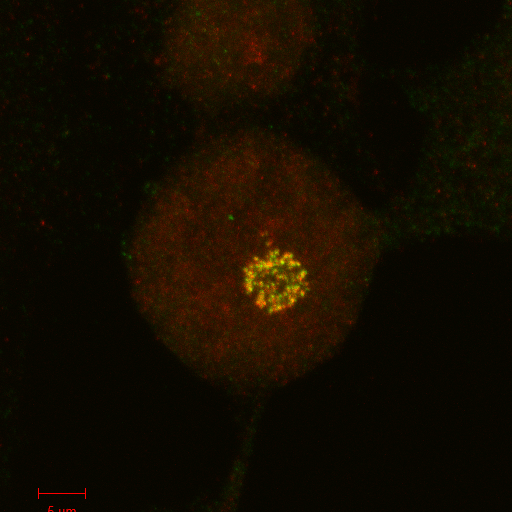

Supplement: Supplementary file 4 — Source Data Fig. 1 [file 44319_2024_106_MOESM4_ESM.zip › Figure 1/1D/Control esiRNA/CENPE488_CENPT568_DAPI_290120_2020_01_29__13_25_51_Maximum intensity projection.tif]

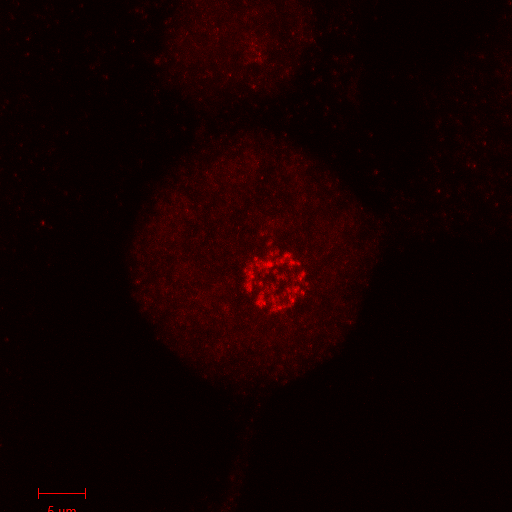

Supplement: Supplementary file 4 — Source Data Fig. 1 [file 44319_2024_106_MOESM4_ESM.zip › Figure 1/1D/Control esiRNA/CENPE488_CENPT568_DAPI_290120_2020_01_29__13_25_51_Maximum intensity projection.tif1.tif]

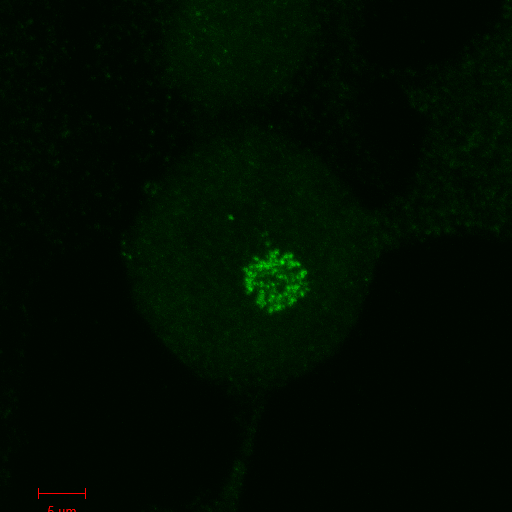

Supplement: Supplementary file 4 — Source Data Fig. 1 [file 44319_2024_106_MOESM4_ESM.zip › Figure 1/1D/Control esiRNA/CENPE488_CENPT568_DAPI_290120_2020_01_29__13_25_51_Maximum intensity projection.tif1.tif2.tif]

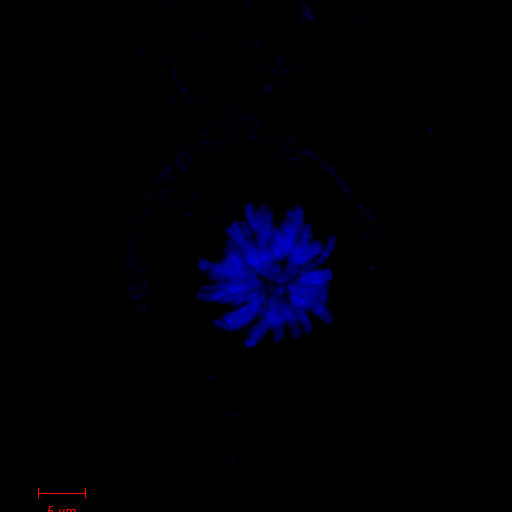

Supplement: Supplementary file 4 — Source Data Fig. 1 [file 44319_2024_106_MOESM4_ESM.zip › Figure 1/1D/Control esiRNA/CENPE488_CENPT568_DAPI_290120_2020_01_29__13_25_51_Maximum intensity projectiontif1.tif2.tif3.tif]

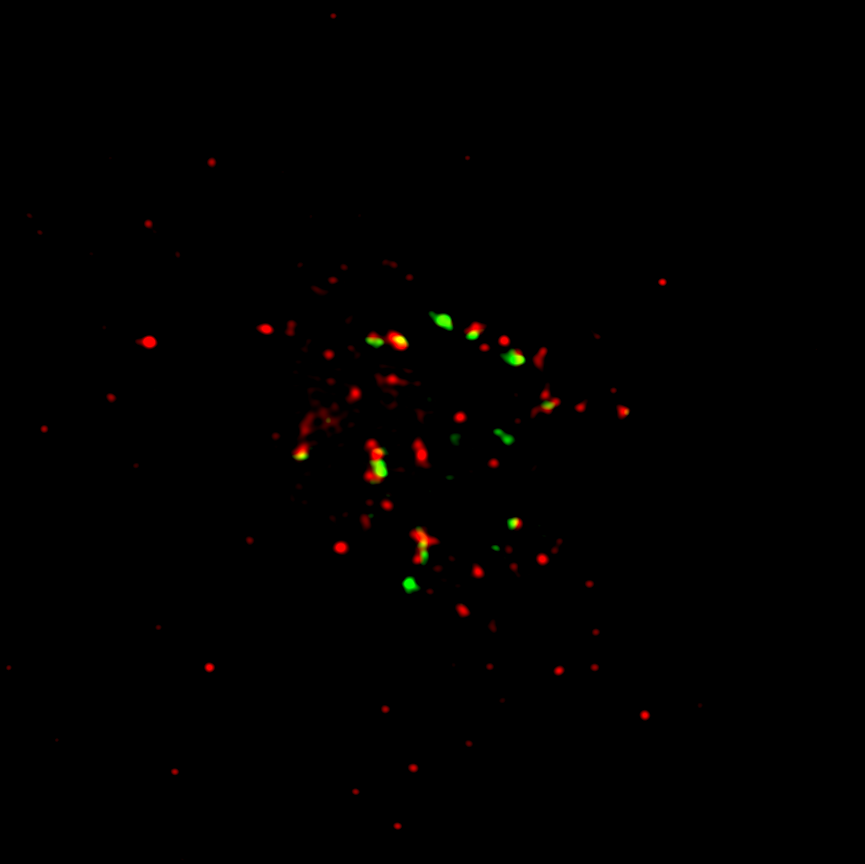

Supplement: Supplementary file 4 — Source Data Fig. 1 [file 44319_2024_106_MOESM4_ESM.zip › Figure 1/1E/CKAP5 esiRNA/Image 9c_Structured Illumination.tif6.tif]

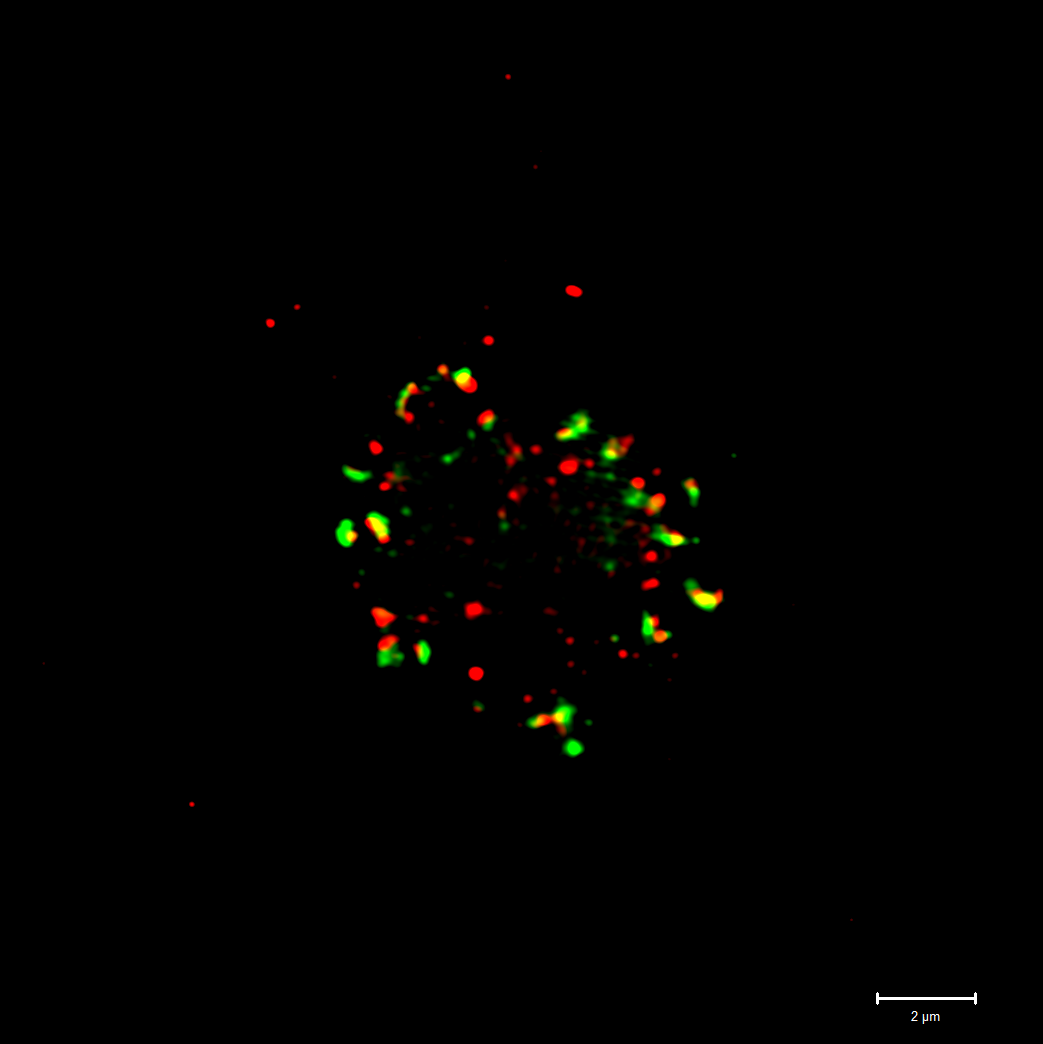

Supplement: Supplementary file 4 — Source Data Fig. 1 [file 44319_2024_106_MOESM4_ESM.zip › Figure 1/1E/Control esiRNA/Image 3c_Structured Illumination.tif6.tif]

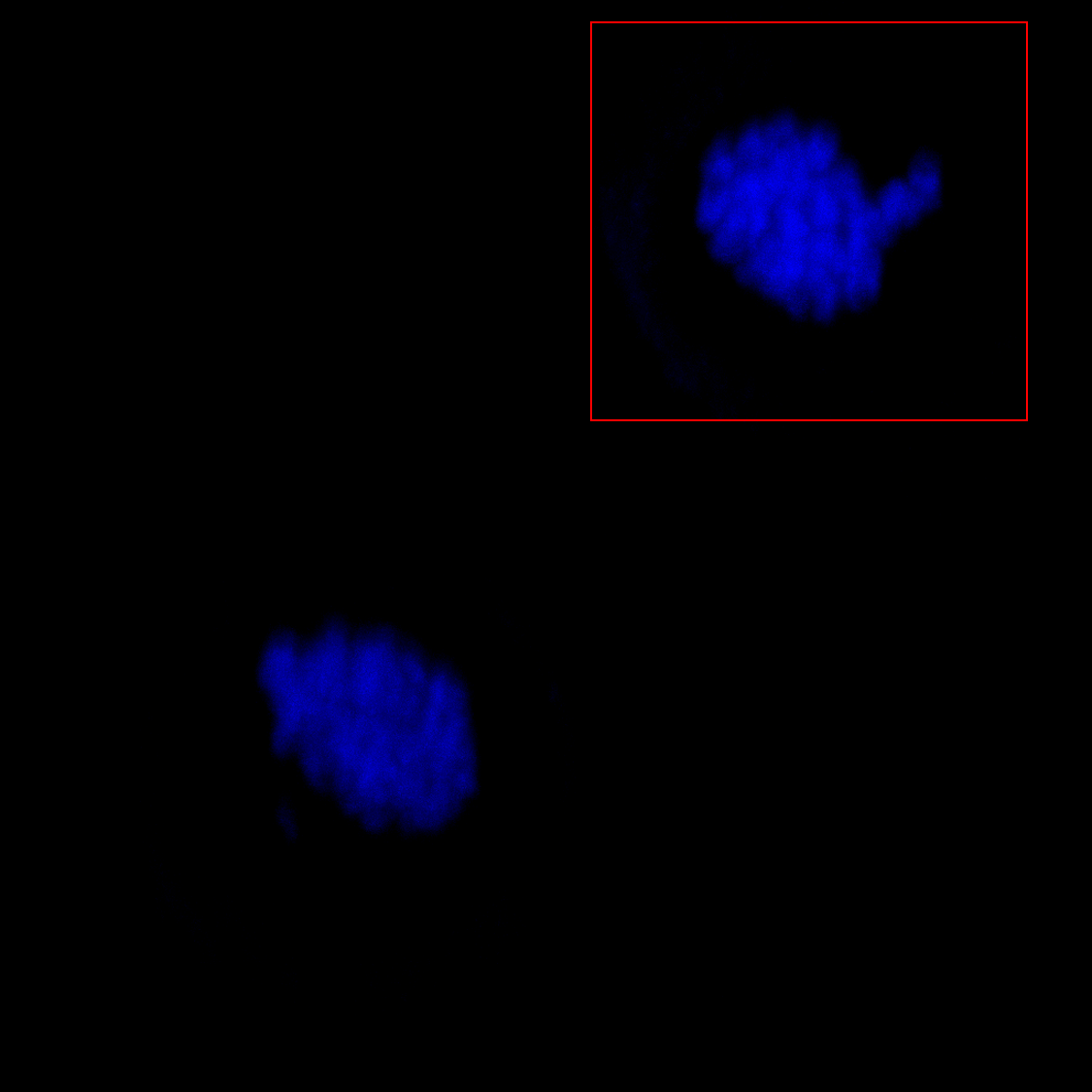

Supplement: Supplementary file 4 — Source Data Fig. 1 [file 44319_2024_106_MOESM4_ESM.zip › Figure 1/1G/CKAP5 esiRNA/Annotation.tif]

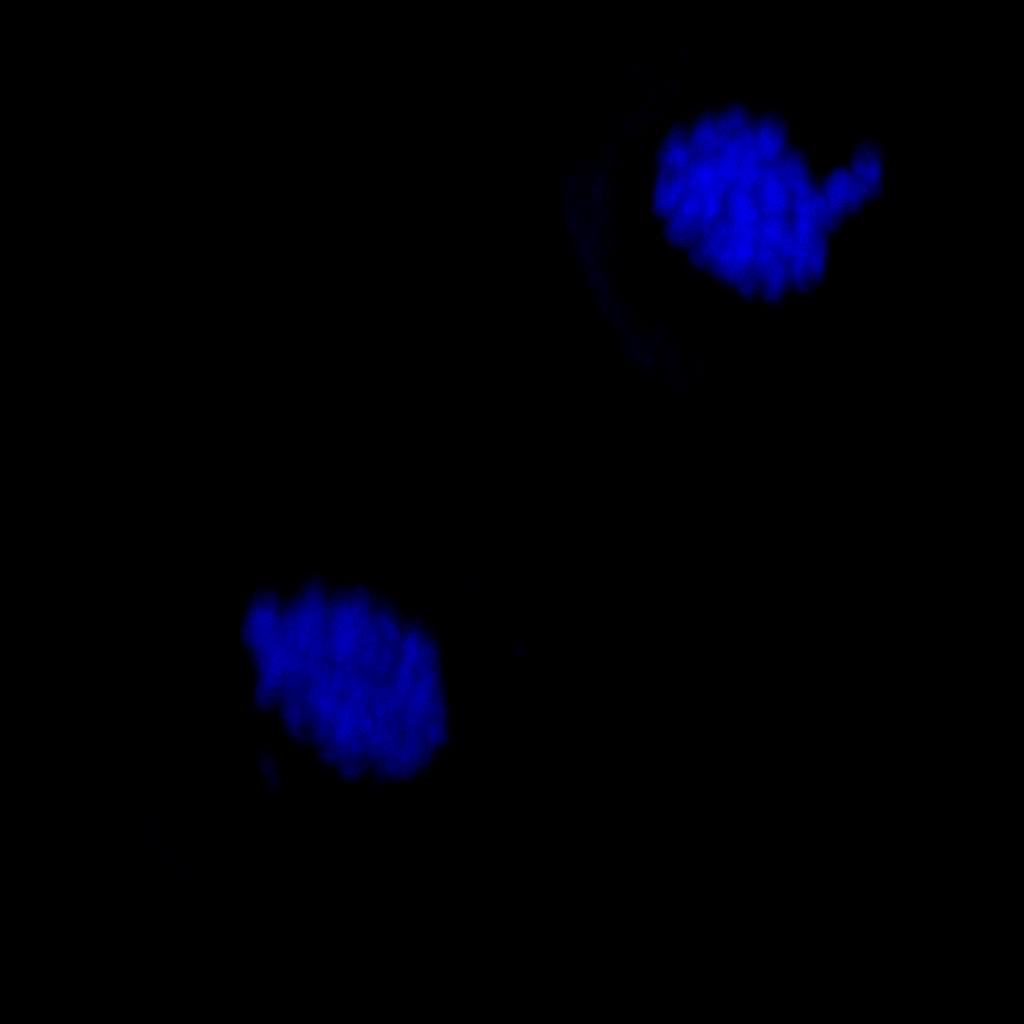

Supplement: Supplementary file 4 — Source Data Fig. 1 [file 44319_2024_106_MOESM4_ESM.zip › Figure 1/1G/CKAP5 esiRNA/Image 10_Maximum intensity projection.tif]

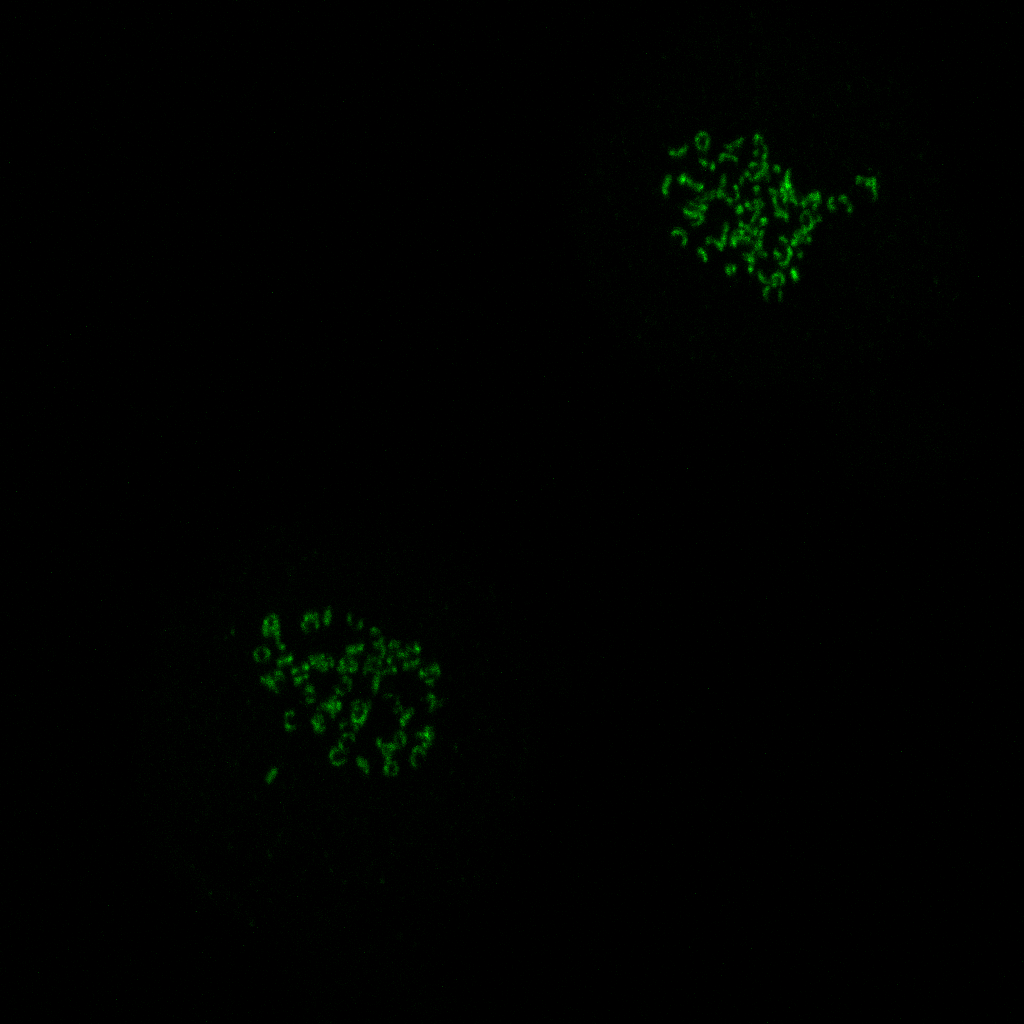

Supplement: Supplementary file 4 — Source Data Fig. 1 [file 44319_2024_106_MOESM4_ESM.zip › Figure 1/1G/CKAP5 esiRNA/Image 10_Maximum intensity projection.tif1.tif]

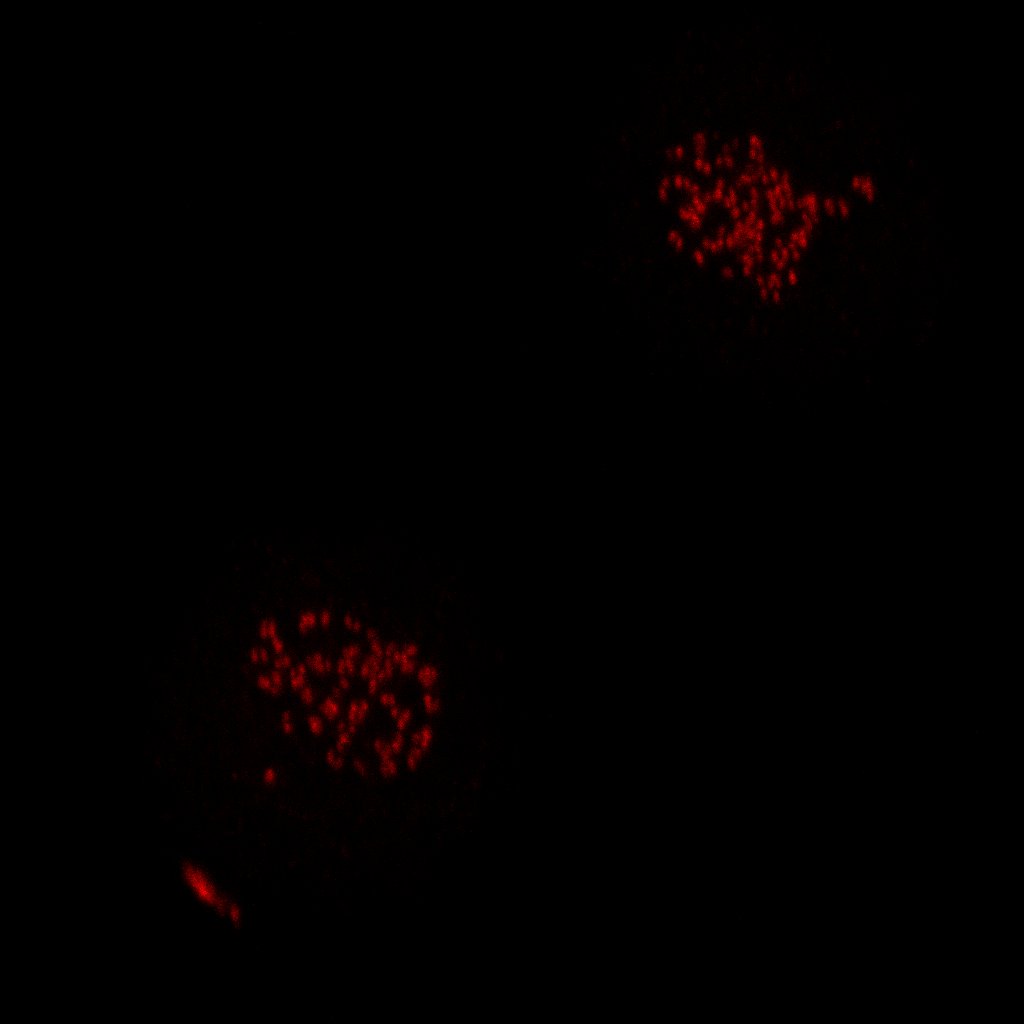

Supplement: Supplementary file 4 — Source Data Fig. 1 [file 44319_2024_106_MOESM4_ESM.zip › Figure 1/1G/CKAP5 esiRNA/Image 10_Maximum intensity projection.tif2.tif]

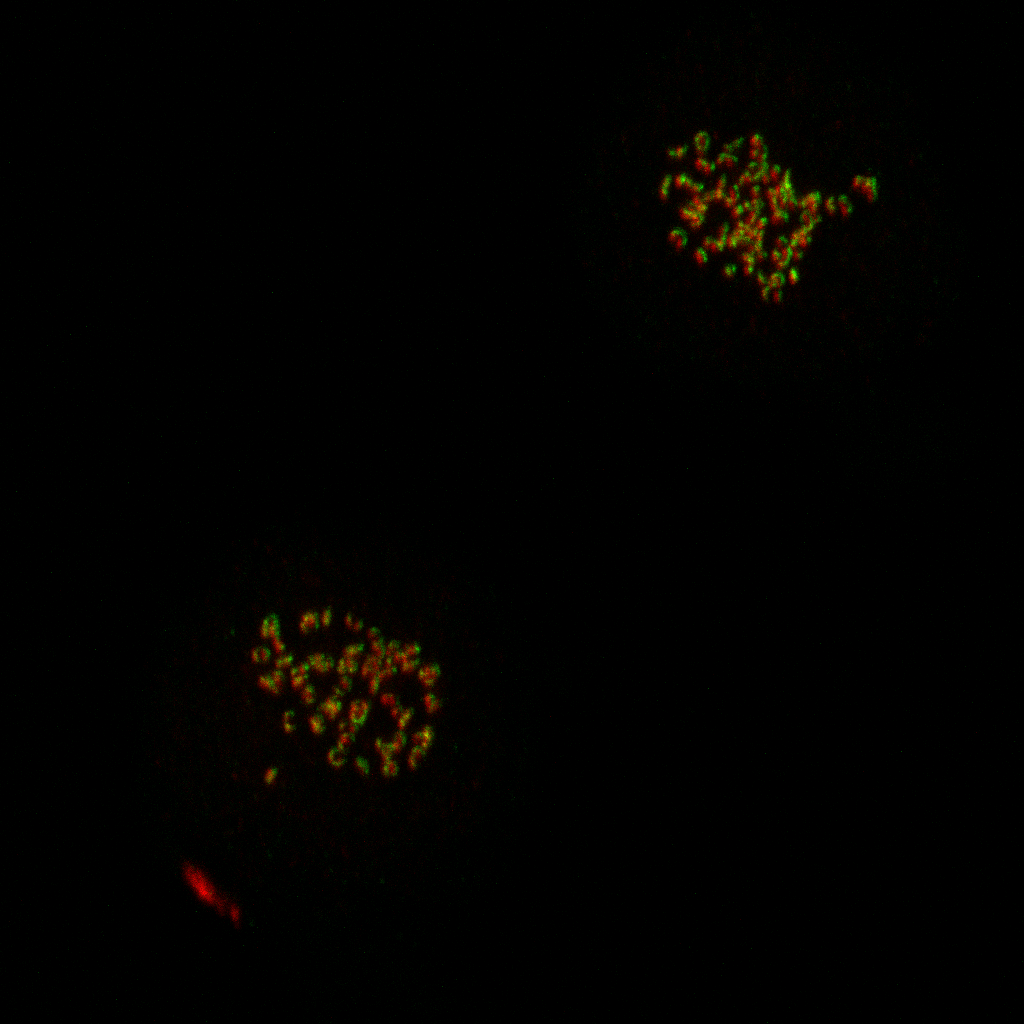

Supplement: Supplementary file 4 — Source Data Fig. 1 [file 44319_2024_106_MOESM4_ESM.zip › Figure 1/1G/CKAP5 esiRNA/Image 10_Maximum intensity projection.tif3.tif]

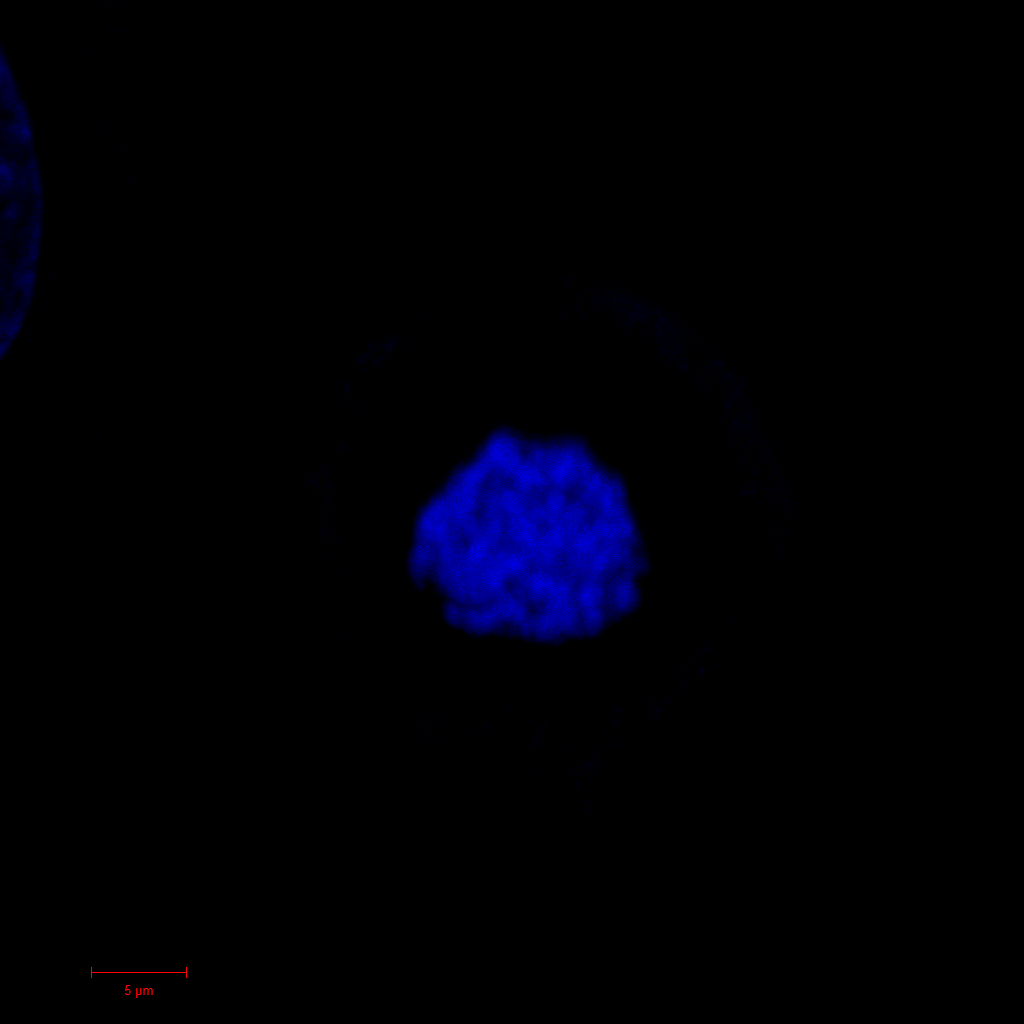

Supplement: Supplementary file 4 — Source Data Fig. 1 [file 44319_2024_106_MOESM4_ESM.zip › Figure 1/1G/Control esiRNA/Image 82_Maximum intensity projection.tif]

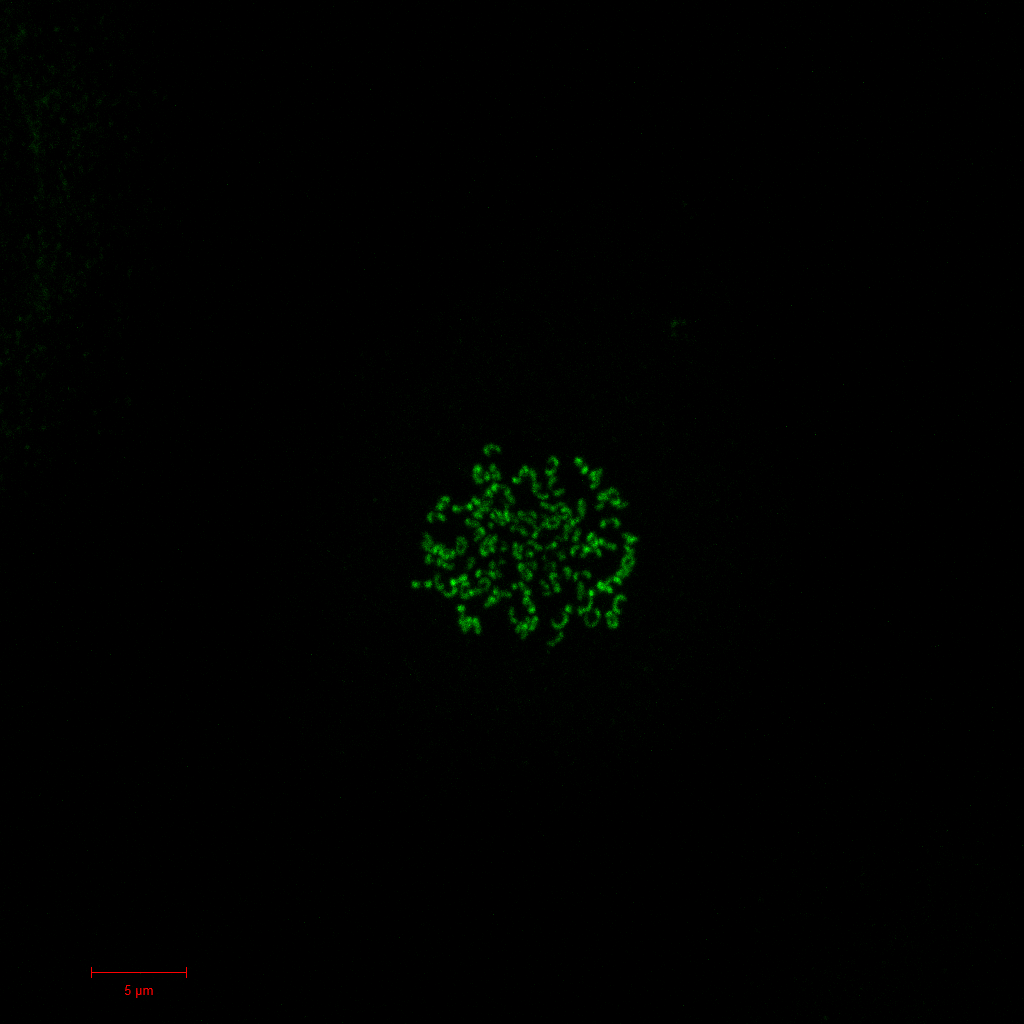

Supplement: Supplementary file 4 — Source Data Fig. 1 [file 44319_2024_106_MOESM4_ESM.zip › Figure 1/1G/Control esiRNA/Image 82_Maximum intensity projection.tif1.tif]

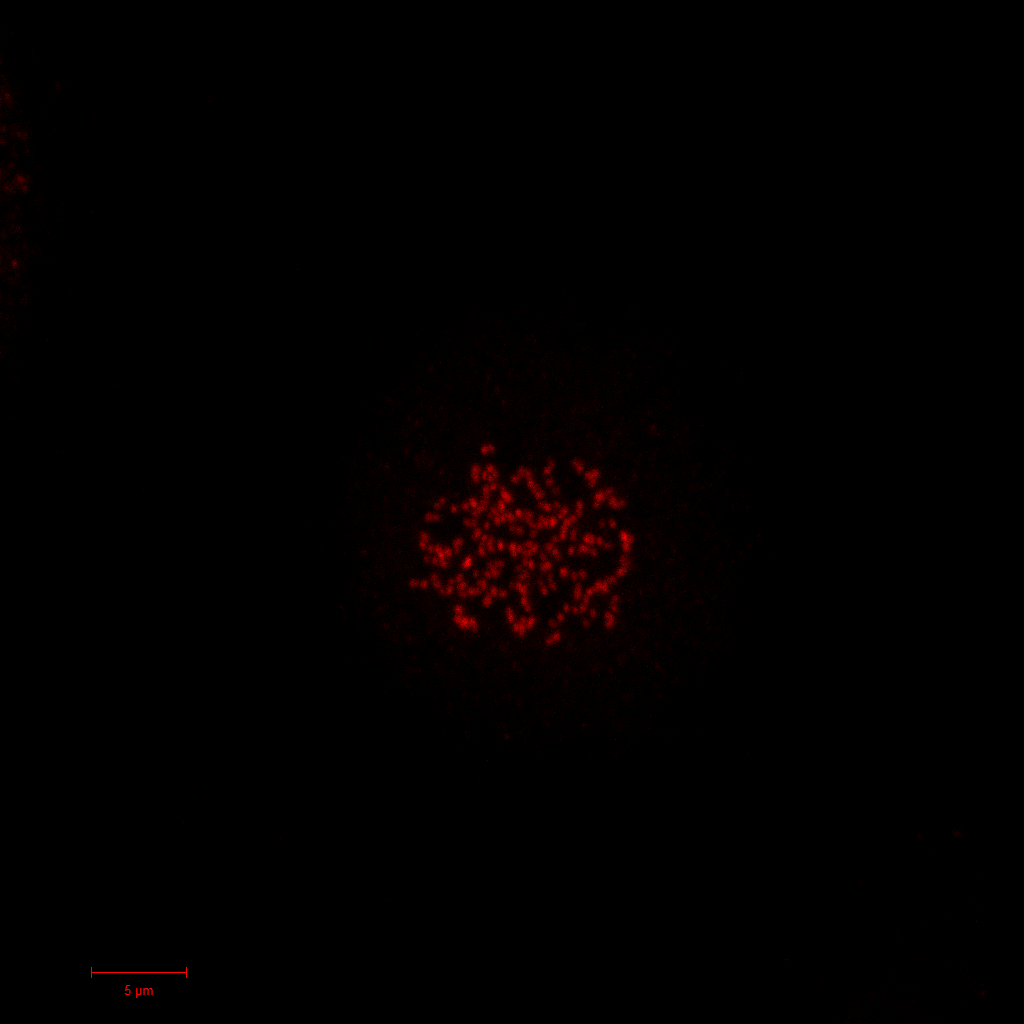

Supplement: Supplementary file 4 — Source Data Fig. 1 [file 44319_2024_106_MOESM4_ESM.zip › Figure 1/1G/Control esiRNA/Image 82_Maximum intensity projection.tif2.tif]

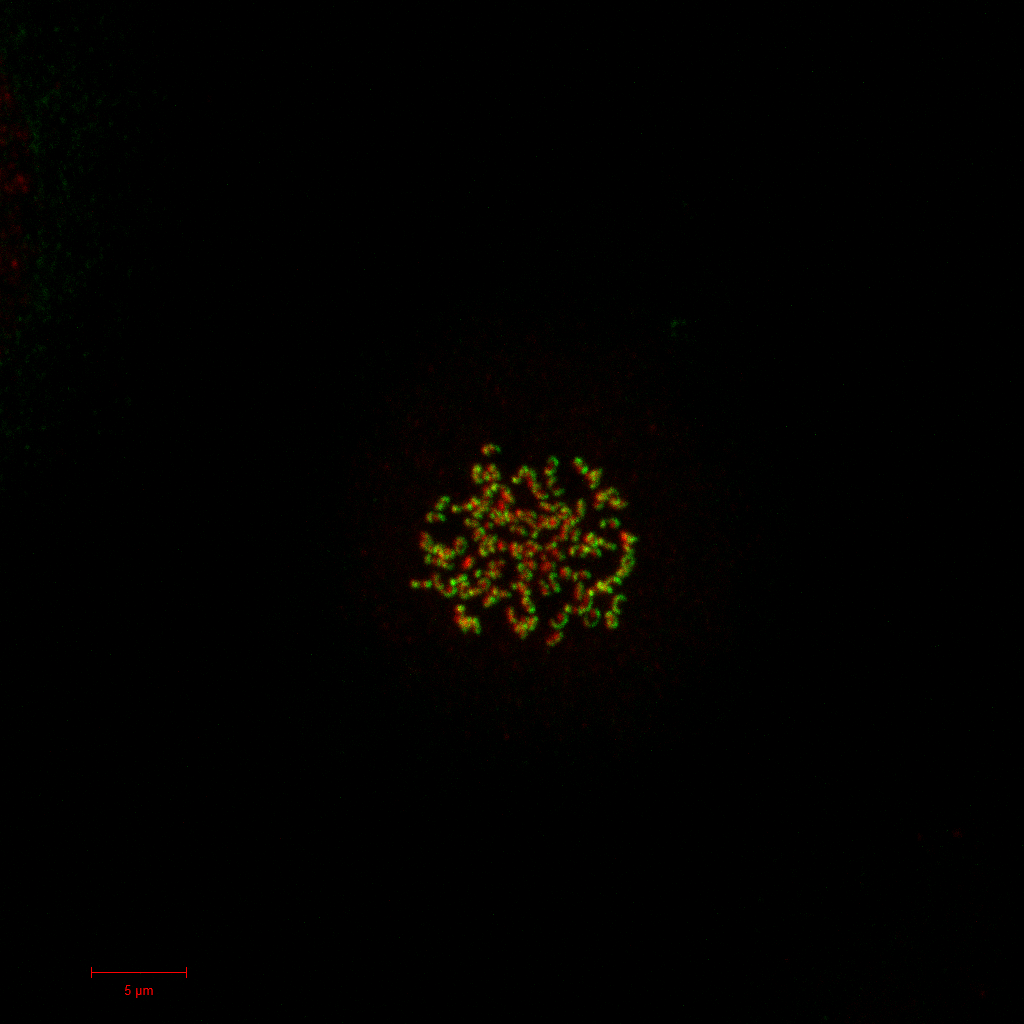

Supplement: Supplementary file 4 — Source Data Fig. 1 [file 44319_2024_106_MOESM4_ESM.zip › Figure 1/1G/Control esiRNA/Image 82_Maximum intensity projection.tif3.tif]

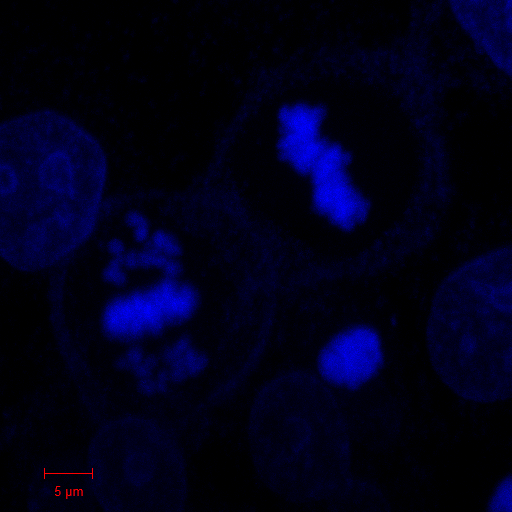

Supplement: Supplementary file 4 — Source Data Fig. 1 [file 44319_2024_106_MOESM4_ESM.zip › Figure 1/1I/1429-2032/Image 27_Maximum intensity projection.tif]

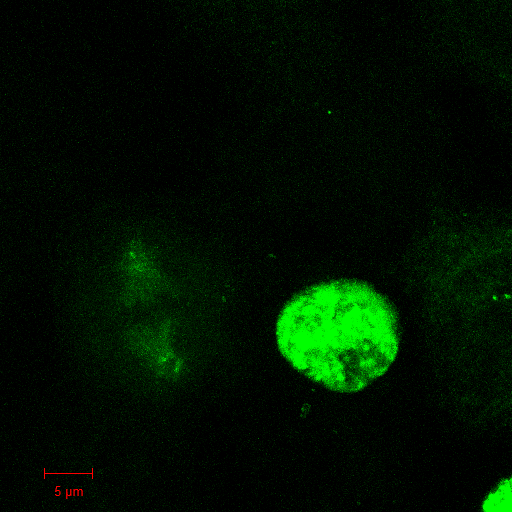

Supplement: Supplementary file 4 — Source Data Fig. 1 [file 44319_2024_106_MOESM4_ESM.zip › Figure 1/1I/1429-2032/Image 27_Maximum intensity projection.tif1.tif]

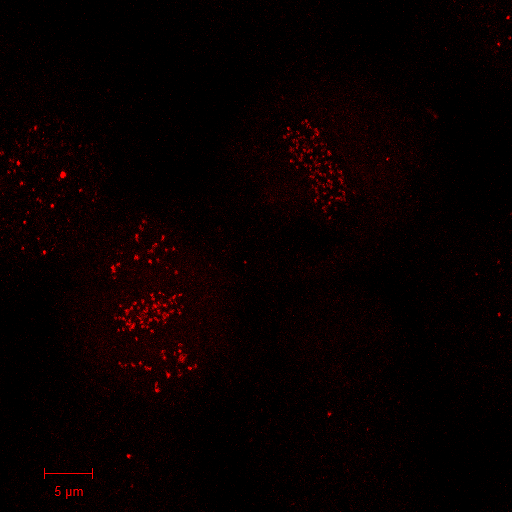

Supplement: Supplementary file 4 — Source Data Fig. 1 [file 44319_2024_106_MOESM4_ESM.zip › Figure 1/1I/1429-2032/Image 27_Maximum intensity projection.tif2.tif]

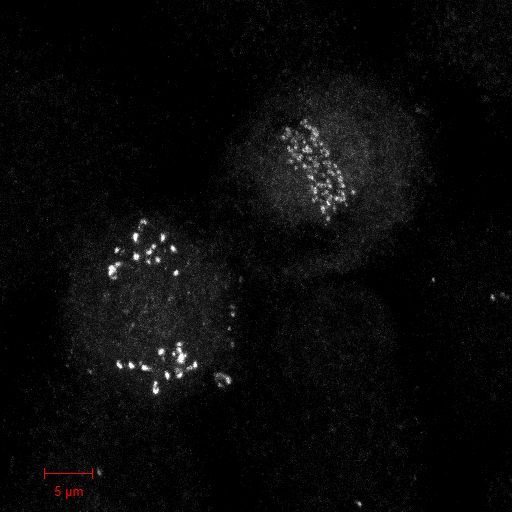

Supplement: Supplementary file 4 — Source Data Fig. 1 [file 44319_2024_106_MOESM4_ESM.zip › Figure 1/1I/1429-2032/Image 27_Maximum intensity projection.tif3.tif]

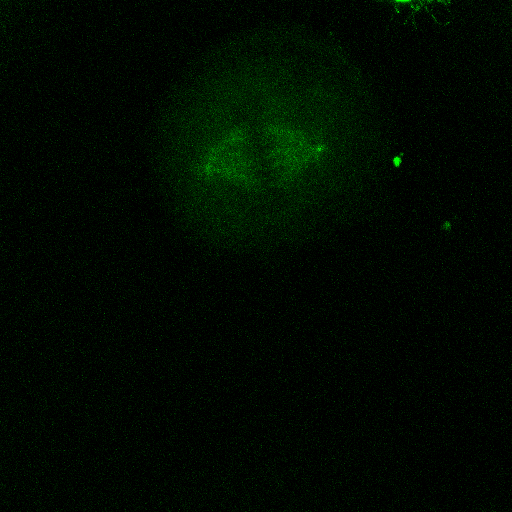

Supplement: Supplementary file 4 — Source Data Fig. 1 [file 44319_2024_106_MOESM4_ESM.zip › Figure 1/1I/853-2032/Image 45_Maximum intensity projection.tif1.tif]

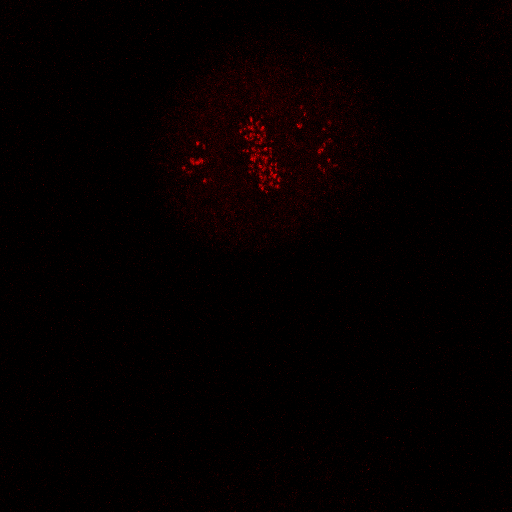

Supplement: Supplementary file 4 — Source Data Fig. 1 [file 44319_2024_106_MOESM4_ESM.zip › Figure 1/1I/853-2032/Image 45_Maximum intensity projection.tif1.tif2.tif]

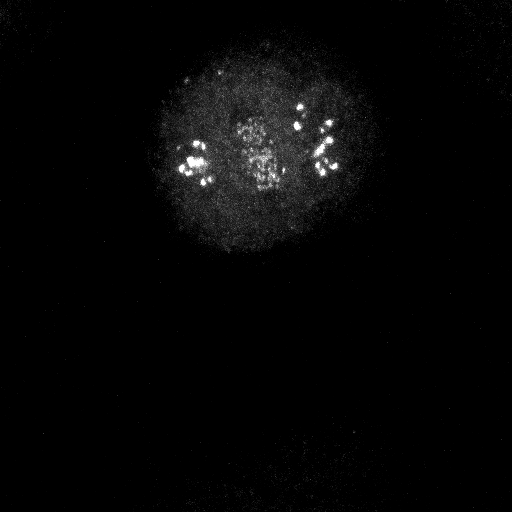

Supplement: Supplementary file 4 — Source Data Fig. 1 [file 44319_2024_106_MOESM4_ESM.zip › Figure 1/1I/853-2032/Image 45_Maximum intensity projectiontif1.tif2.tif3.tif]

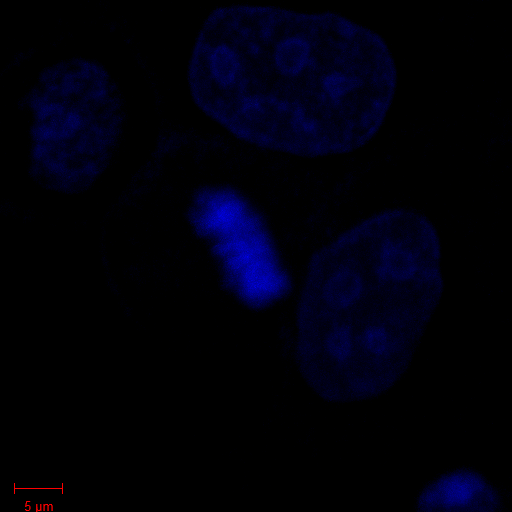

Supplement: Supplementary file 4 — Source Data Fig. 1 [file 44319_2024_106_MOESM4_ESM.zip › Figure 1/1I/FL/Image 35_Maximum intensity projection.tif]

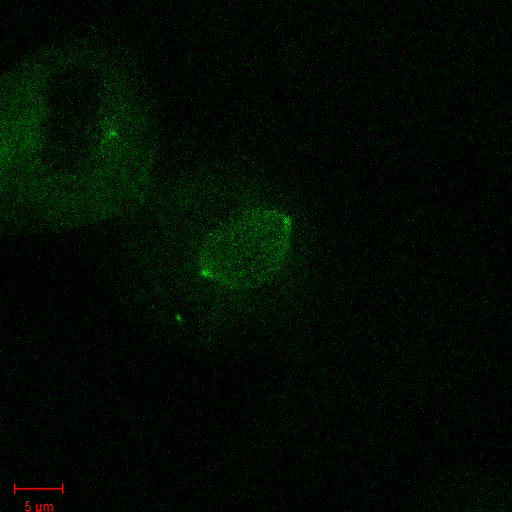

Supplement: Supplementary file 4 — Source Data Fig. 1 [file 44319_2024_106_MOESM4_ESM.zip › Figure 1/1I/FL/Image 35_Maximum intensity projection.tif1.tif]

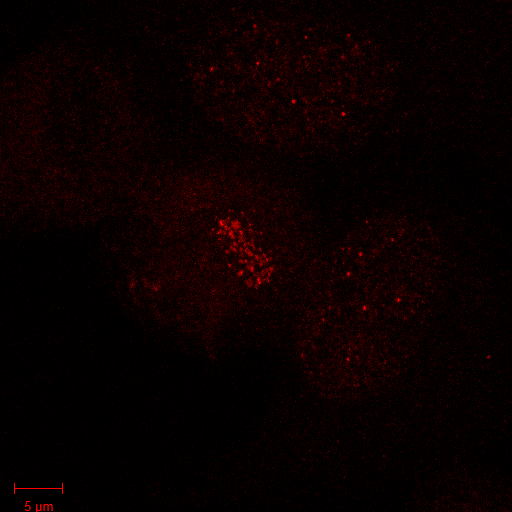

Supplement: Supplementary file 4 — Source Data Fig. 1 [file 44319_2024_106_MOESM4_ESM.zip › Figure 1/1I/FL/Image 35_Maximum intensity projection.tif1.tif2.tif]

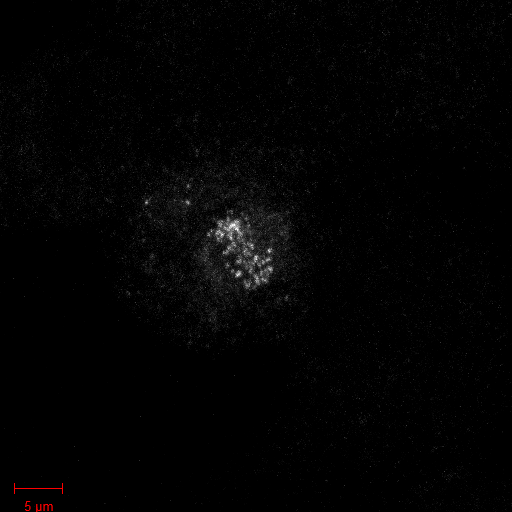

Supplement: Supplementary file 4 — Source Data Fig. 1 [file 44319_2024_106_MOESM4_ESM.zip › Figure 1/1I/FL/Image 35_Maximum intensity projectiontif1.tif2.tif3.tif]

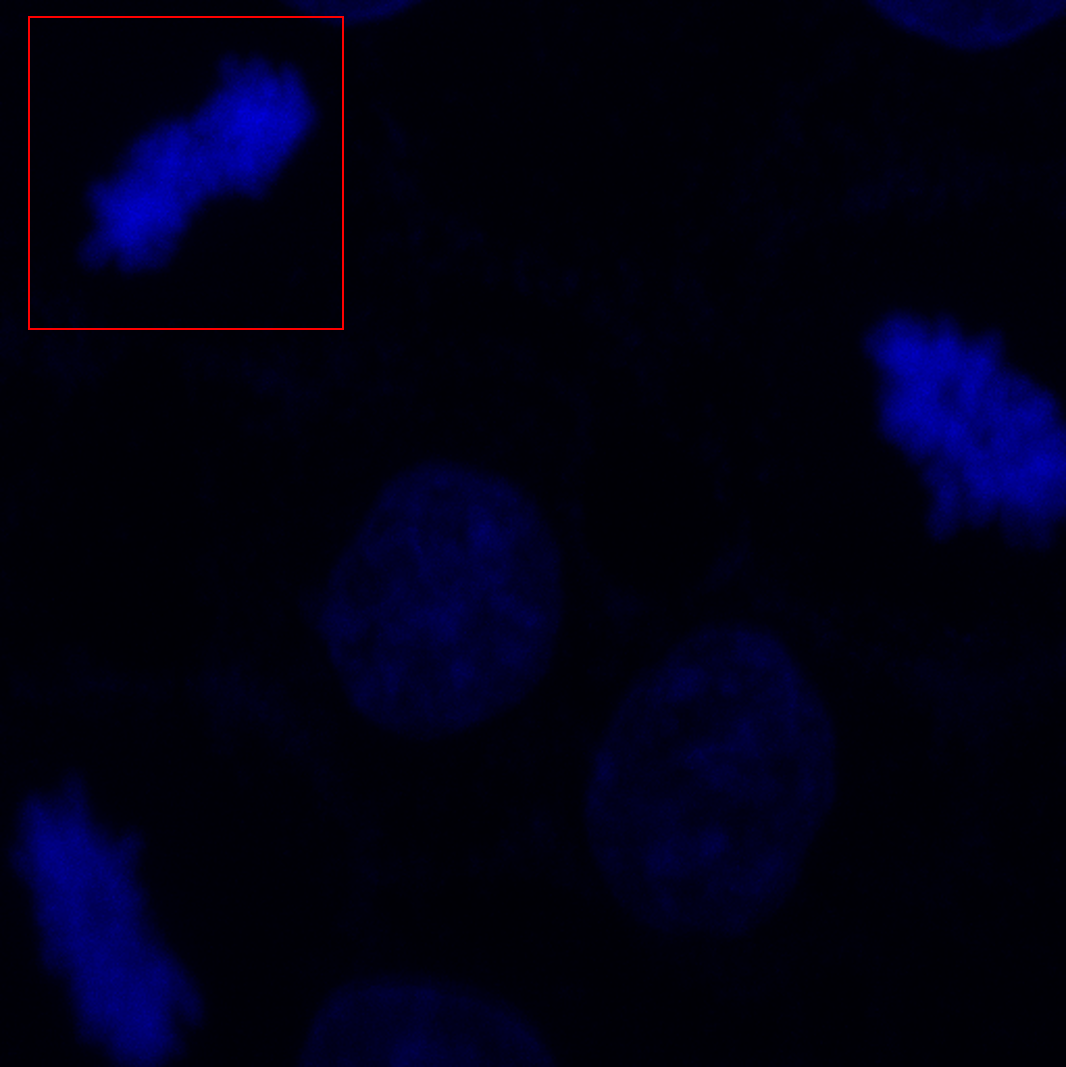

Supplement: Supplementary file 4 — Source Data Fig. 1 [file 44319_2024_106_MOESM4_ESM.zip › Figure 1/1I/Untransfected/Annotation.tif]

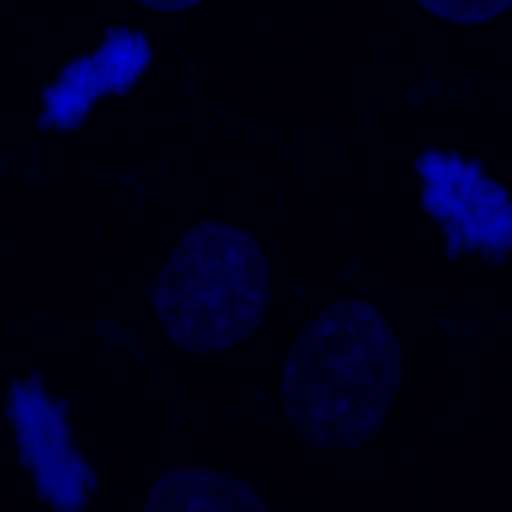

Supplement: Supplementary file 4 — Source Data Fig. 1 [file 44319_2024_106_MOESM4_ESM.zip › Figure 1/1I/Untransfected/Image 56_Maximum intensity projection.tif]

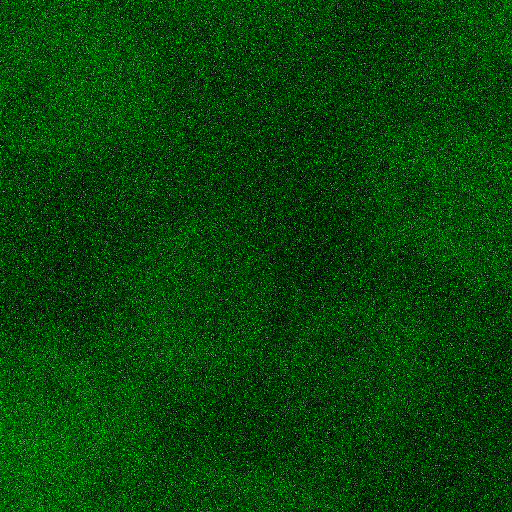

Supplement: Supplementary file 4 — Source Data Fig. 1 [file 44319_2024_106_MOESM4_ESM.zip › Figure 1/1I/Untransfected/Image 56_Maximum intensity projection.tif1.tif]

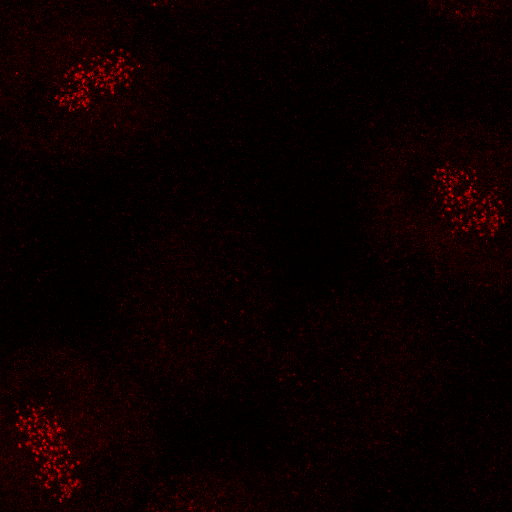

Supplement: Supplementary file 4 — Source Data Fig. 1 [file 44319_2024_106_MOESM4_ESM.zip › Figure 1/1I/Untransfected/Image 56_Maximum intensity projection.tif2.tif]

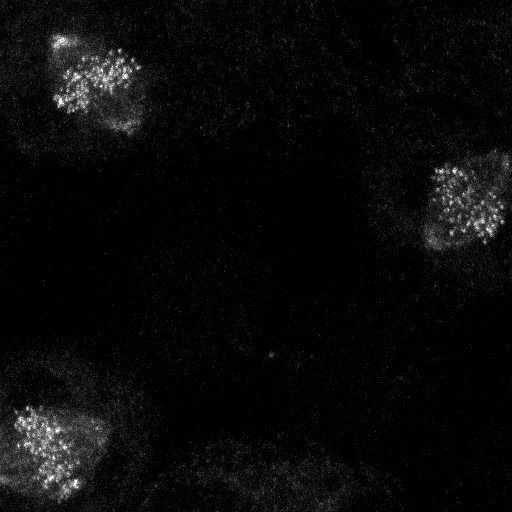

Supplement: Supplementary file 4 — Source Data Fig. 1 [file 44319_2024_106_MOESM4_ESM.zip › Figure 1/1I/Untransfected/Image 56_Maximum intensity projection.tif3.tif]

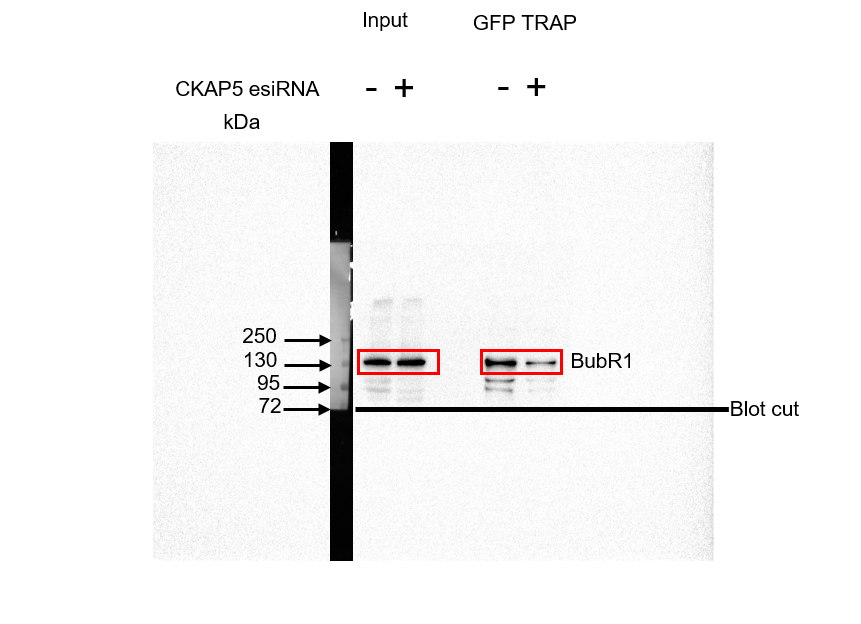

Supplement: Supplementary file 5 — Source Data Fig. 2 [file 44319_2024_106_MOESM5_ESM.zip › Figure 2/2A/BubR1/Annotation.tif]

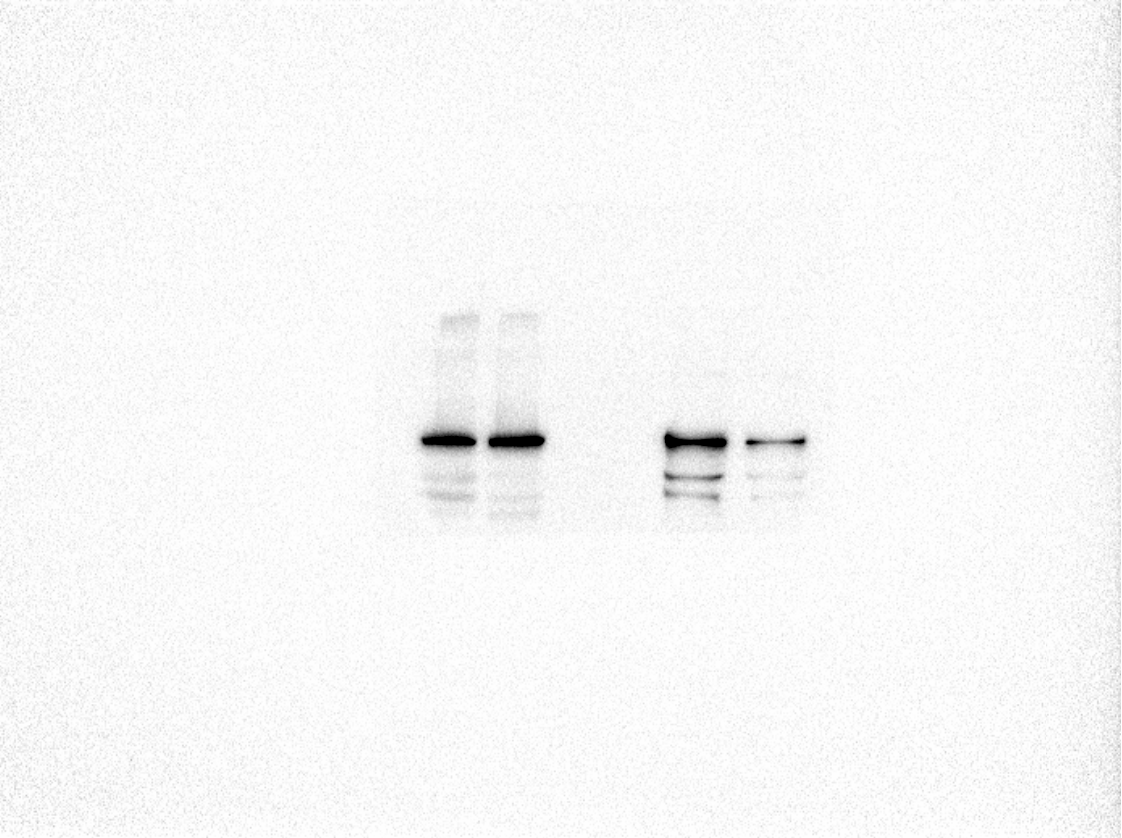

Supplement: Supplementary file 5 — Source Data Fig. 2 [file 44319_2024_106_MOESM5_ESM.zip › Figure 2/2A/BubR1/INPUT_TRAP.tif]

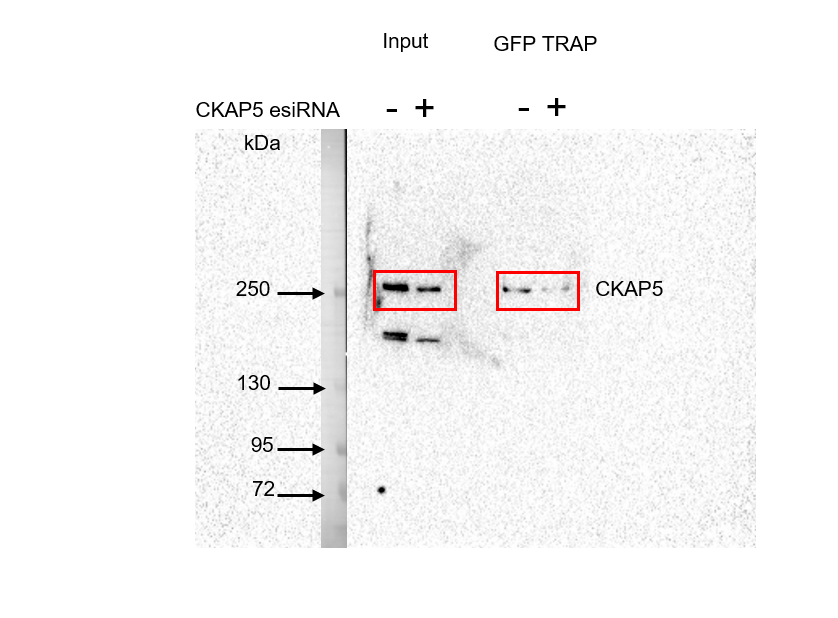

Supplement: Supplementary file 5 — Source Data Fig. 2 [file 44319_2024_106_MOESM5_ESM.zip › Figure 2/2A/CKAP5/Annotation.tif]

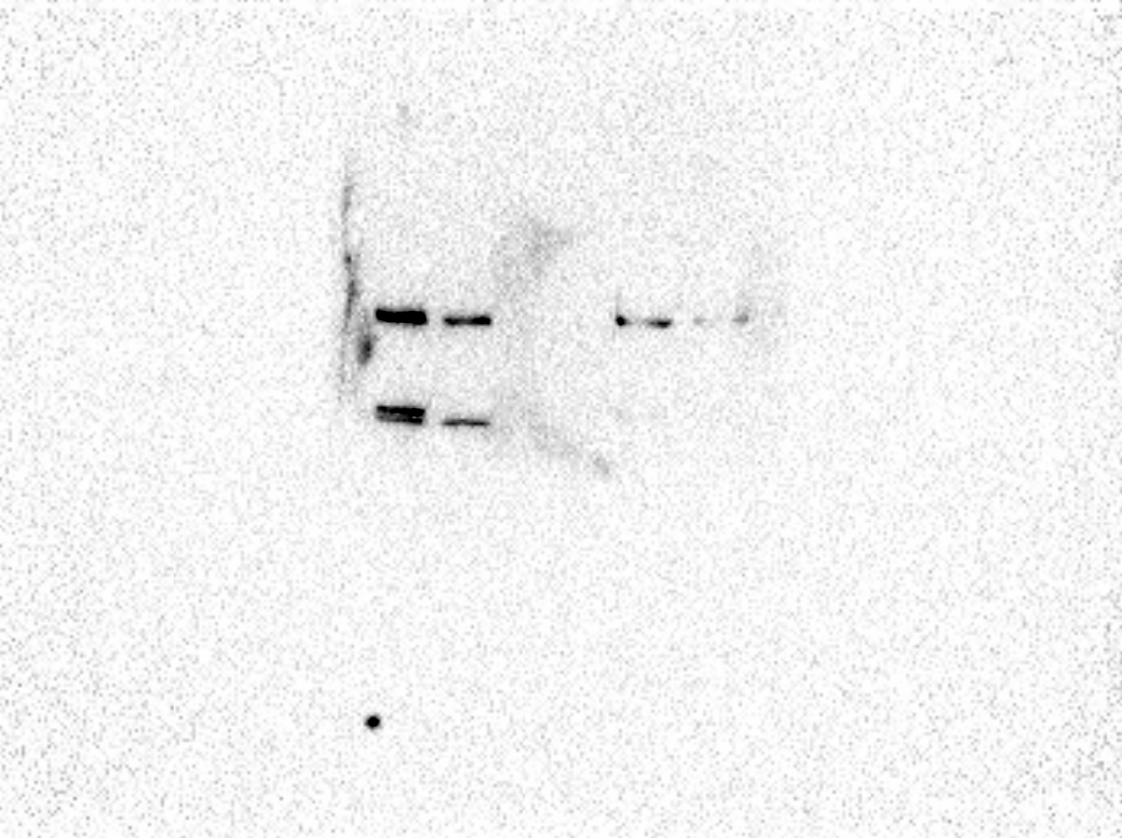

Supplement: Supplementary file 5 — Source Data Fig. 2 [file 44319_2024_106_MOESM5_ESM.zip › Figure 2/2A/CKAP5/INPUT_TRAP.tif]

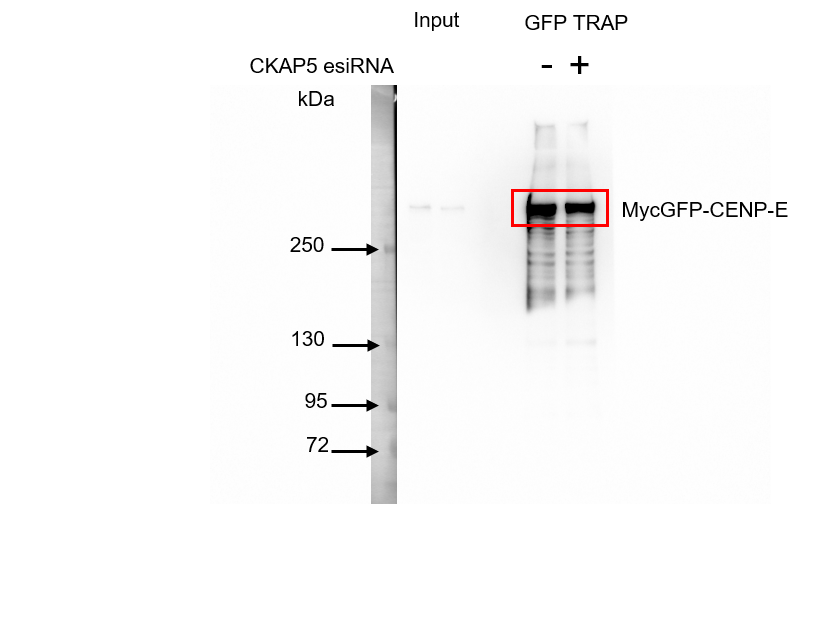

Supplement: Supplementary file 5 — Source Data Fig. 2 [file 44319_2024_106_MOESM5_ESM.zip › Figure 2/2A/MycGFP-CENP-E/GFP TRAP/Annotation.tif]

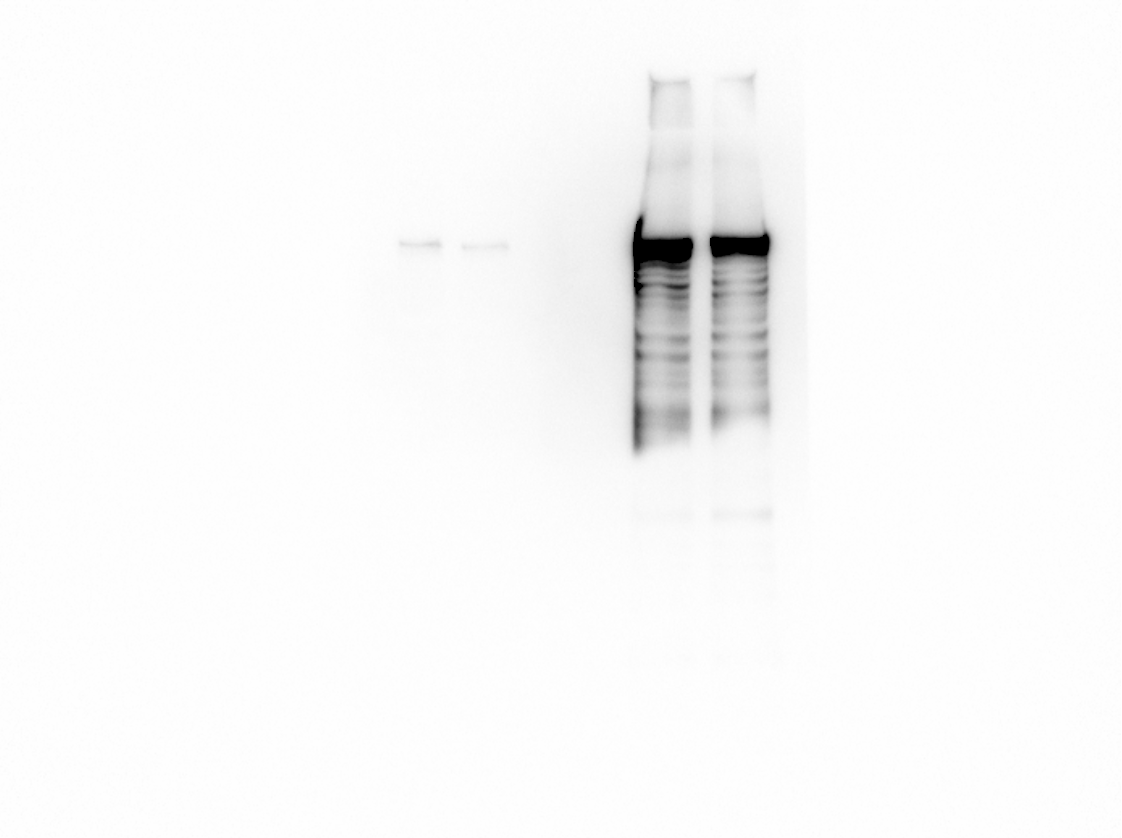

Supplement: Supplementary file 5 — Source Data Fig. 2 [file 44319_2024_106_MOESM5_ESM.zip › Figure 2/2A/MycGFP-CENP-E/GFP TRAP/TRAP.tif]

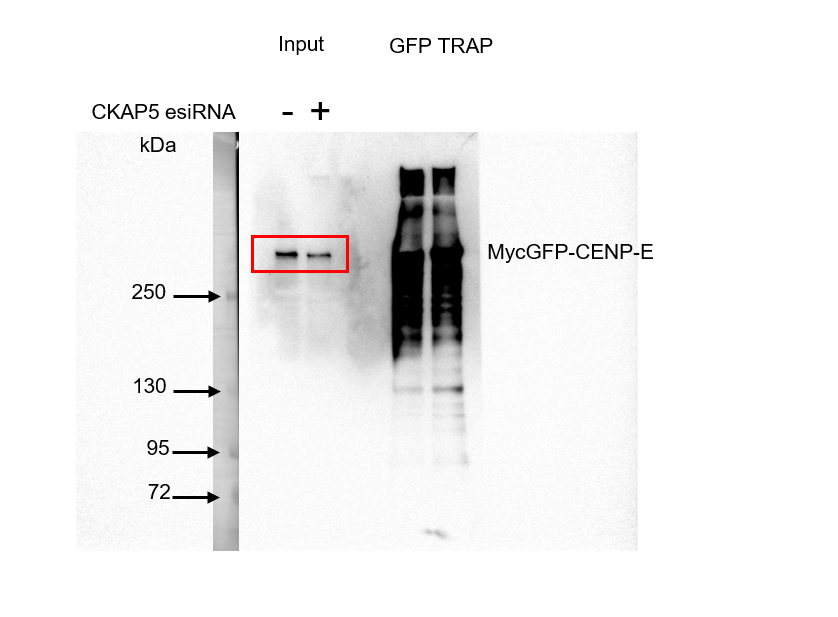

Supplement: Supplementary file 5 — Source Data Fig. 2 [file 44319_2024_106_MOESM5_ESM.zip › Figure 2/2A/MycGFP-CENP-E/INPUT/Annotation.tif]

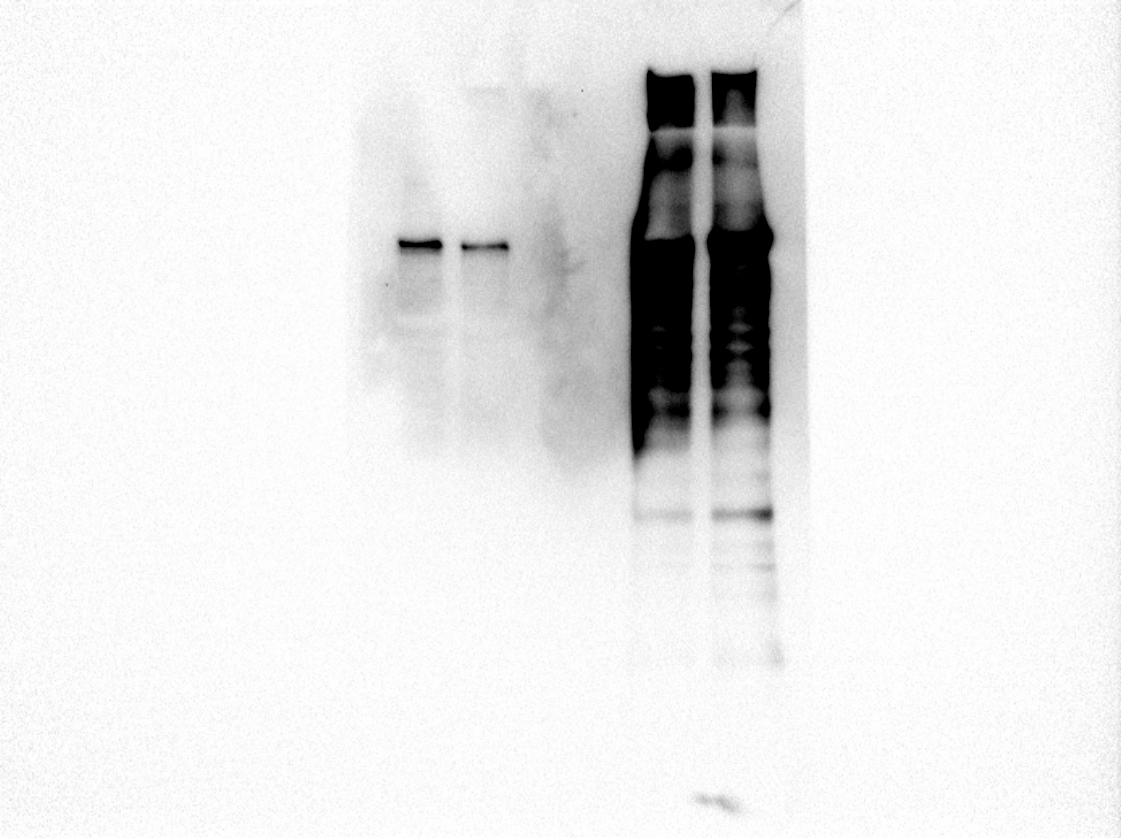

Supplement: Supplementary file 5 — Source Data Fig. 2 [file 44319_2024_106_MOESM5_ESM.zip › Figure 2/2A/MycGFP-CENP-E/INPUT/INPUT.tif]

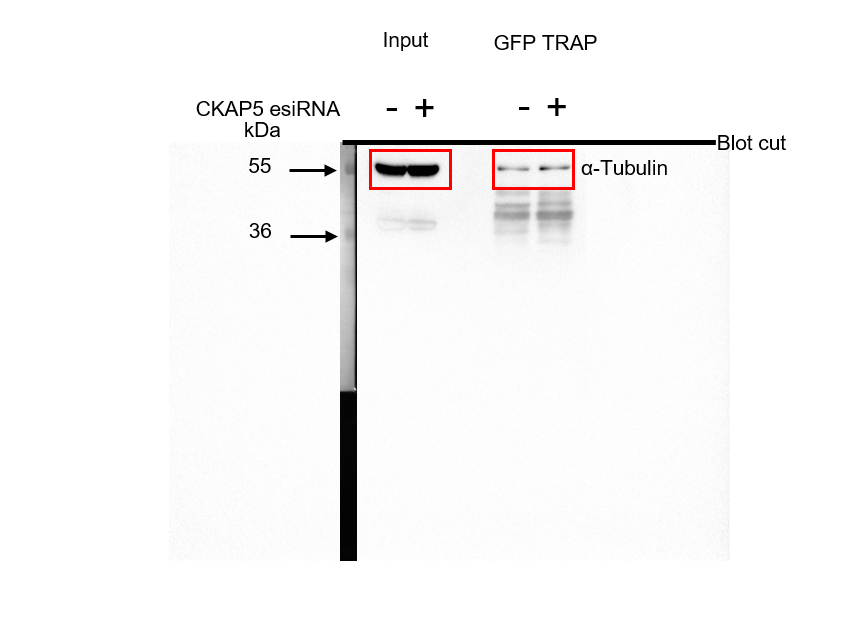

Supplement: Supplementary file 5 — Source Data Fig. 2 [file 44319_2024_106_MOESM5_ESM.zip › Figure 2/2A/tubulin/Annotation.tif]

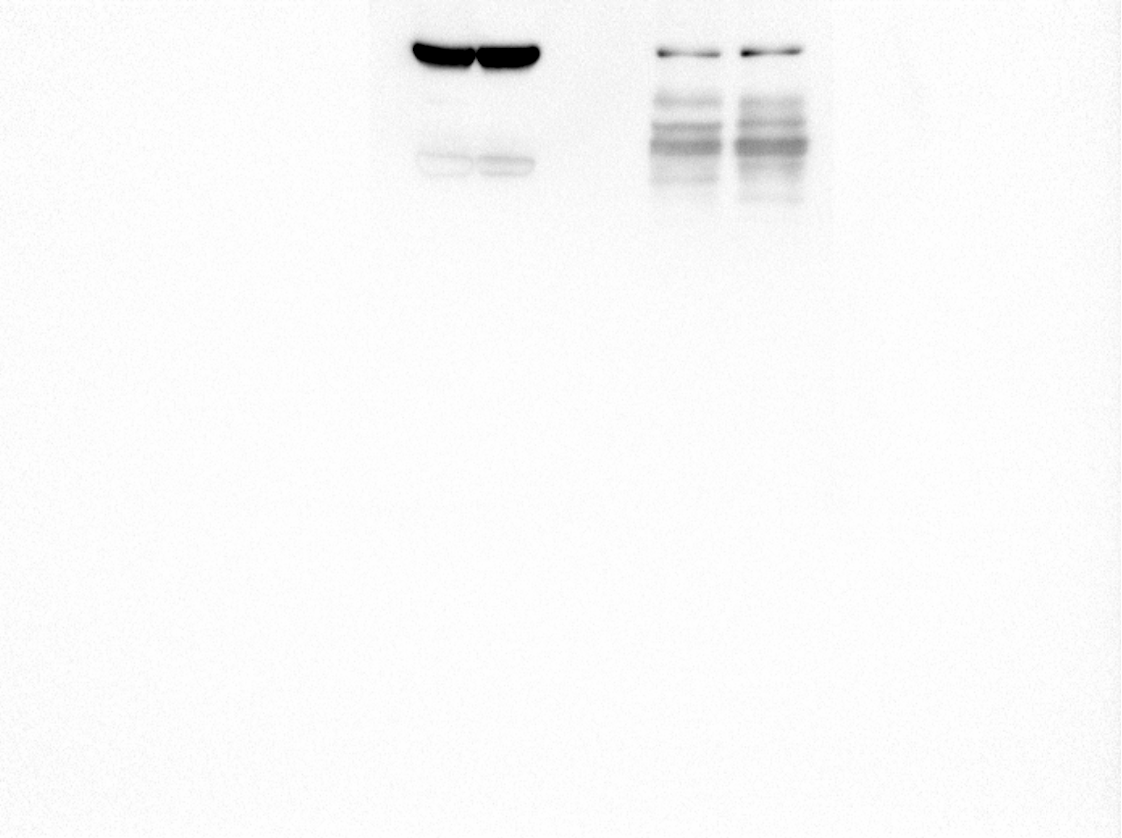

Supplement: Supplementary file 5 — Source Data Fig. 2 [file 44319_2024_106_MOESM5_ESM.zip › Figure 2/2A/tubulin/INPUT_TRAP.tif]

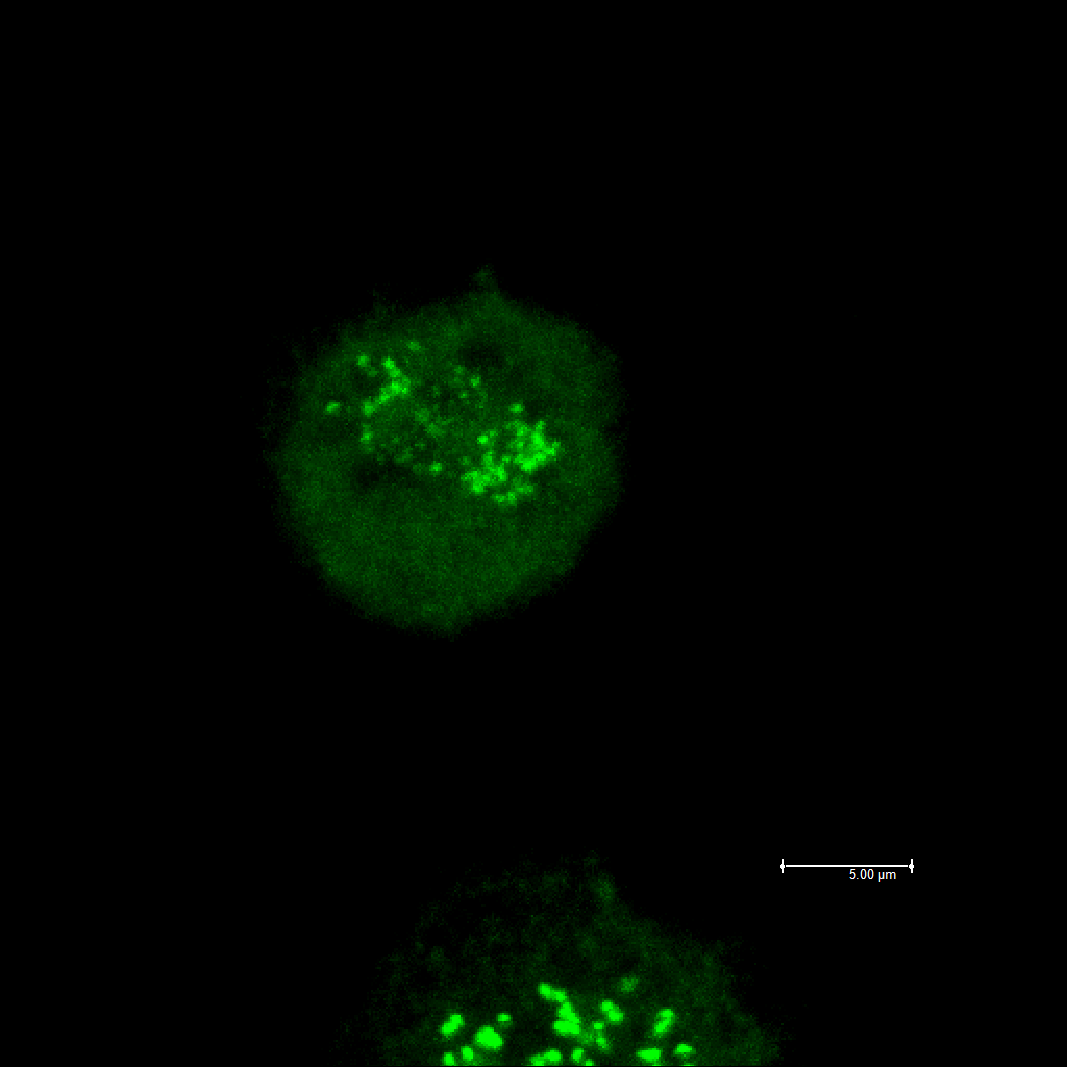

Supplement: Supplementary file 5 — Source Data Fig. 2 [file 44319_2024_106_MOESM5_ESM.zip › Figure 2/2C/CKAP5 esiRNA/togesi_bubr1cy5_knl1568_n=1_121122.lif_Series014Snapshot1_ch00.tif]

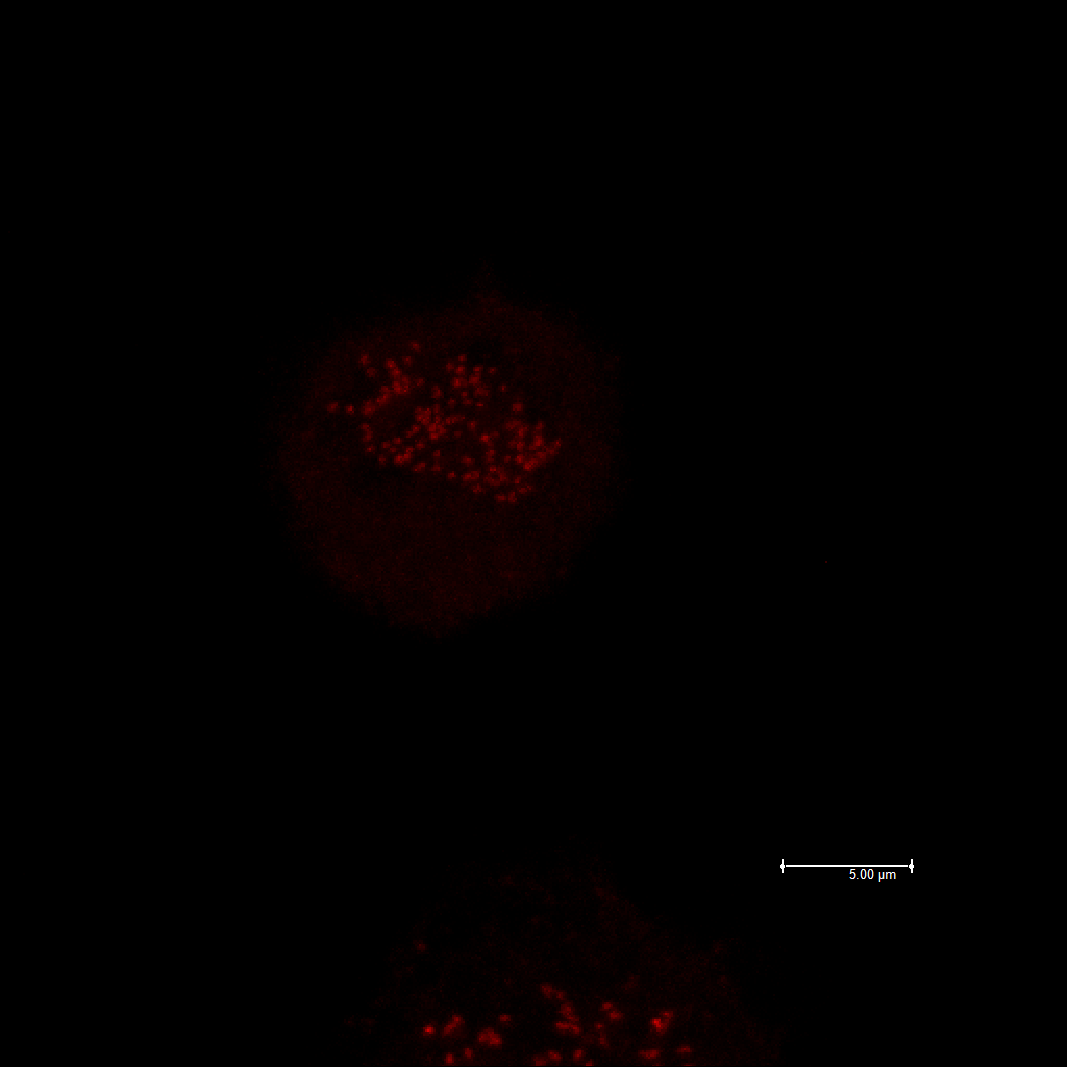

Supplement: Supplementary file 5 — Source Data Fig. 2 [file 44319_2024_106_MOESM5_ESM.zip › Figure 2/2C/CKAP5 esiRNA/togesi_bubr1cy5_knl1568_n=1_121122.lif_Series014Snapshot2_ch00.tif]

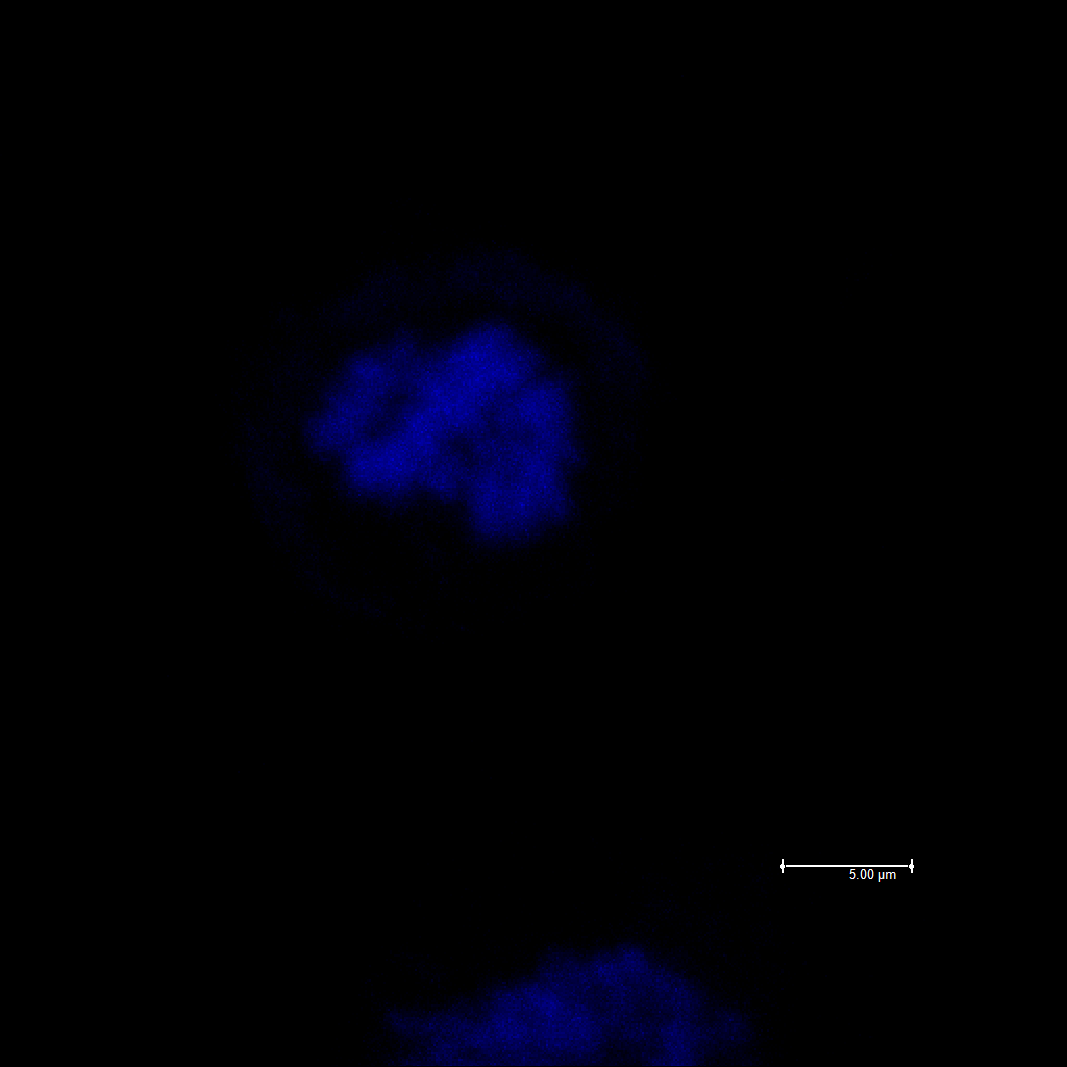

Supplement: Supplementary file 5 — Source Data Fig. 2 [file 44319_2024_106_MOESM5_ESM.zip › Figure 2/2C/CKAP5 esiRNA/togesi_bubr1cy5_knl1568_n=1_121122.lif_Series014Snapshot3_ch00.tif]

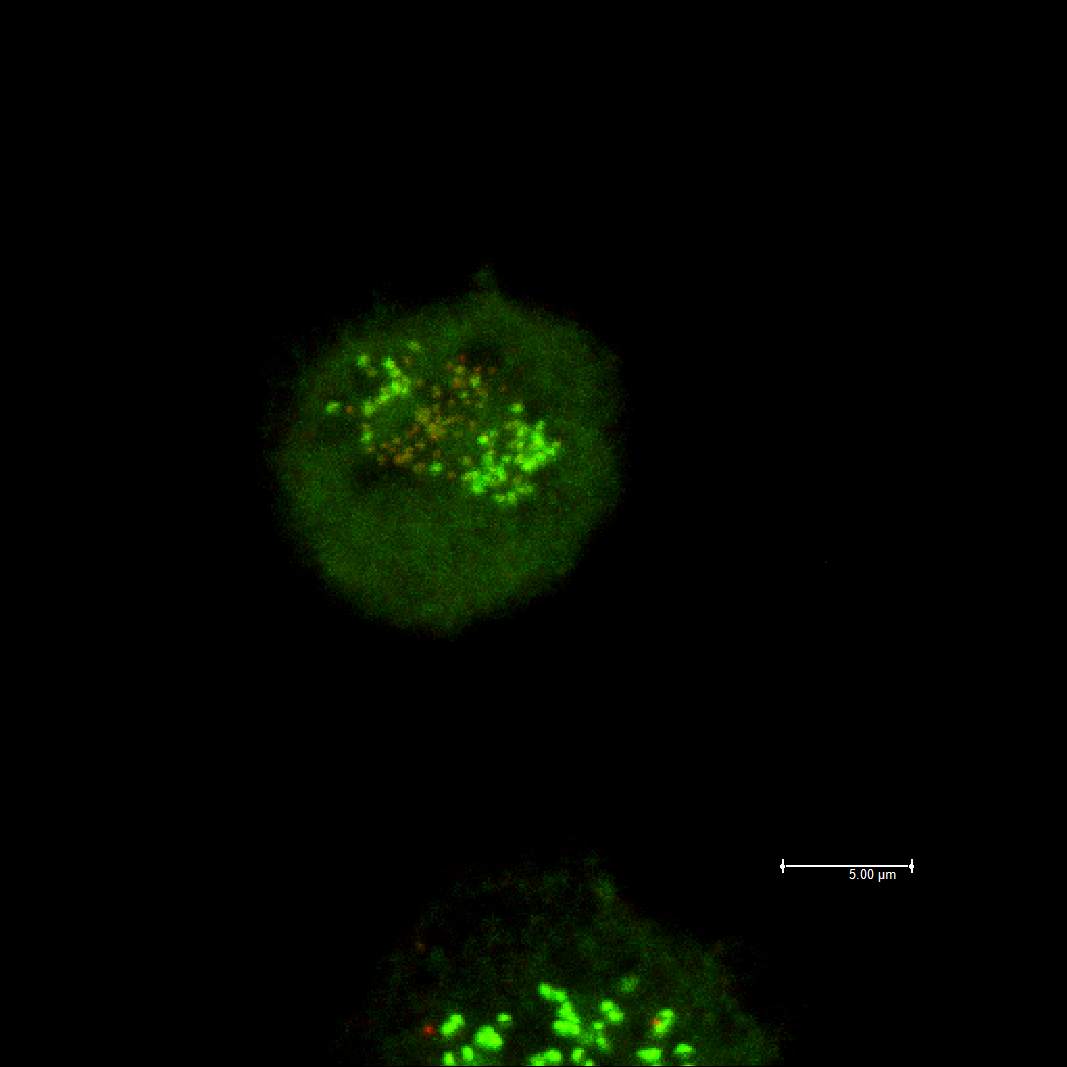

Supplement: Supplementary file 5 — Source Data Fig. 2 [file 44319_2024_106_MOESM5_ESM.zip › Figure 2/2C/CKAP5 esiRNA/togesi_bubr1cy5_knl1568_n=1_121122.lif_Series014Snapshot4_ch00.tif]

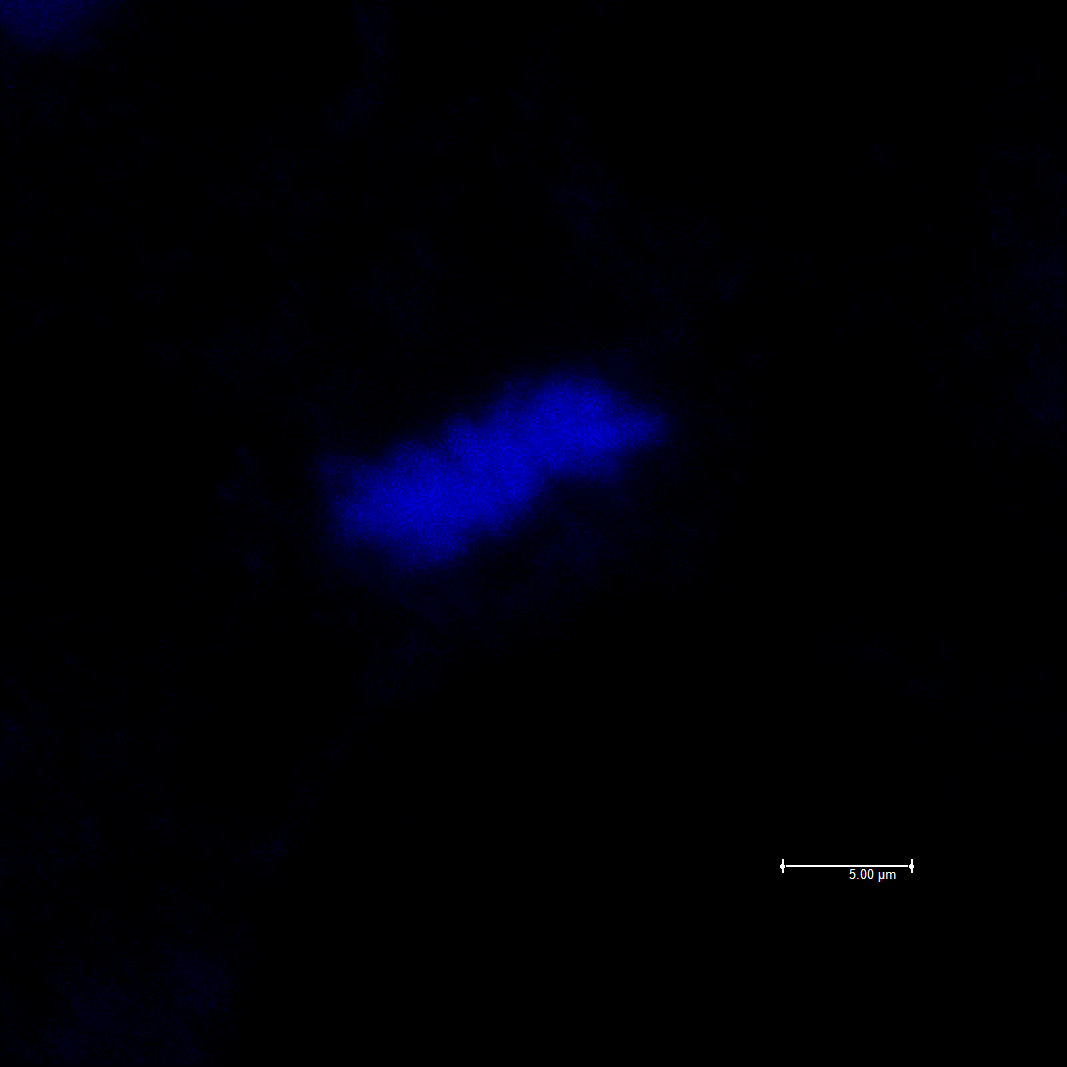

Supplement: Supplementary file 5 — Source Data Fig. 2 [file 44319_2024_106_MOESM5_ESM.zip › Figure 2/2C/Control esiRNA/ctrlesi_bubr1cy5_knl1568_n=1_121122.lif_Series010Snapshot1_ch00.tif]

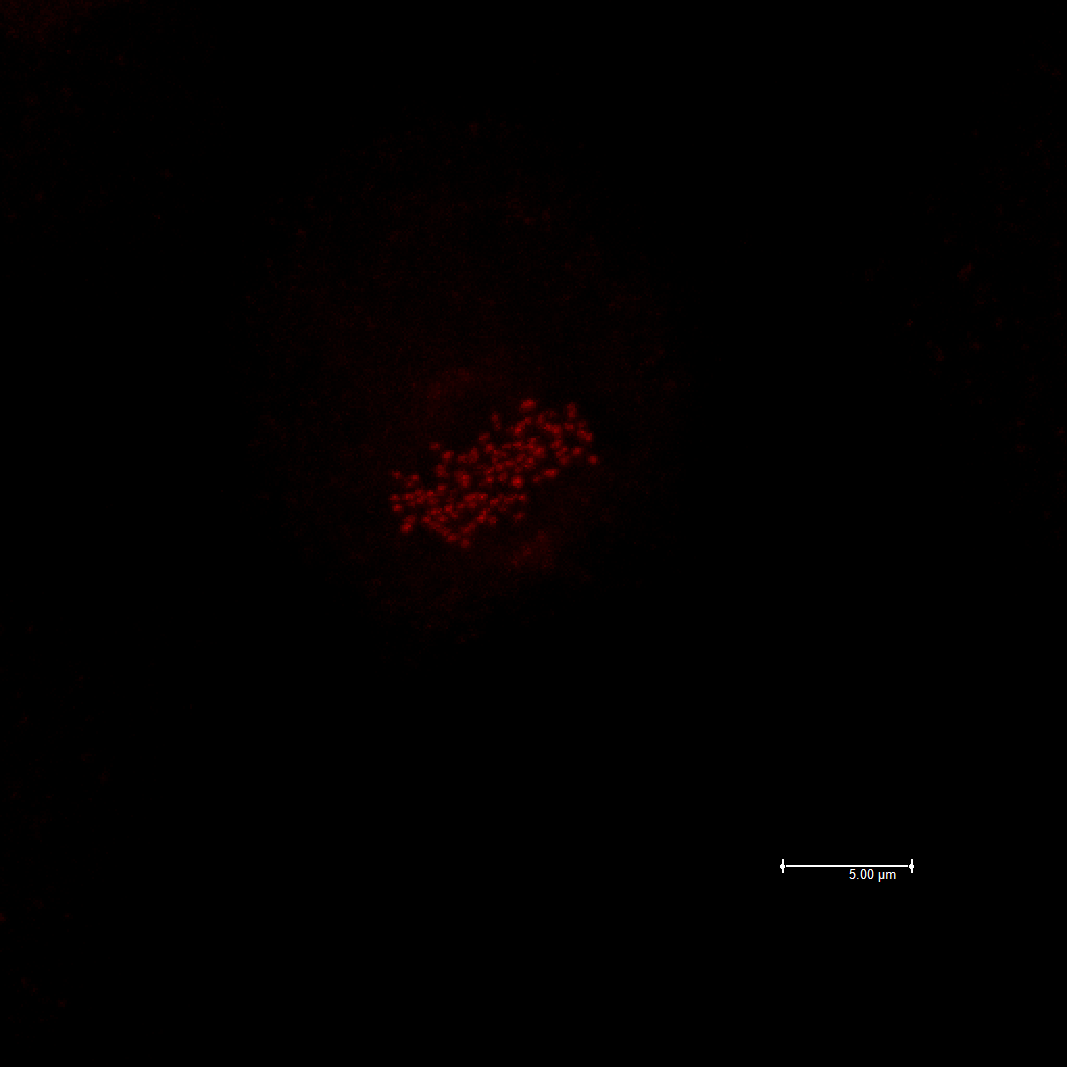

Supplement: Supplementary file 5 — Source Data Fig. 2 [file 44319_2024_106_MOESM5_ESM.zip › Figure 2/2C/Control esiRNA/ctrlesi_bubr1cy5_knl1568_n=1_121122.lif_Series010Snapshot2_ch00.tif]

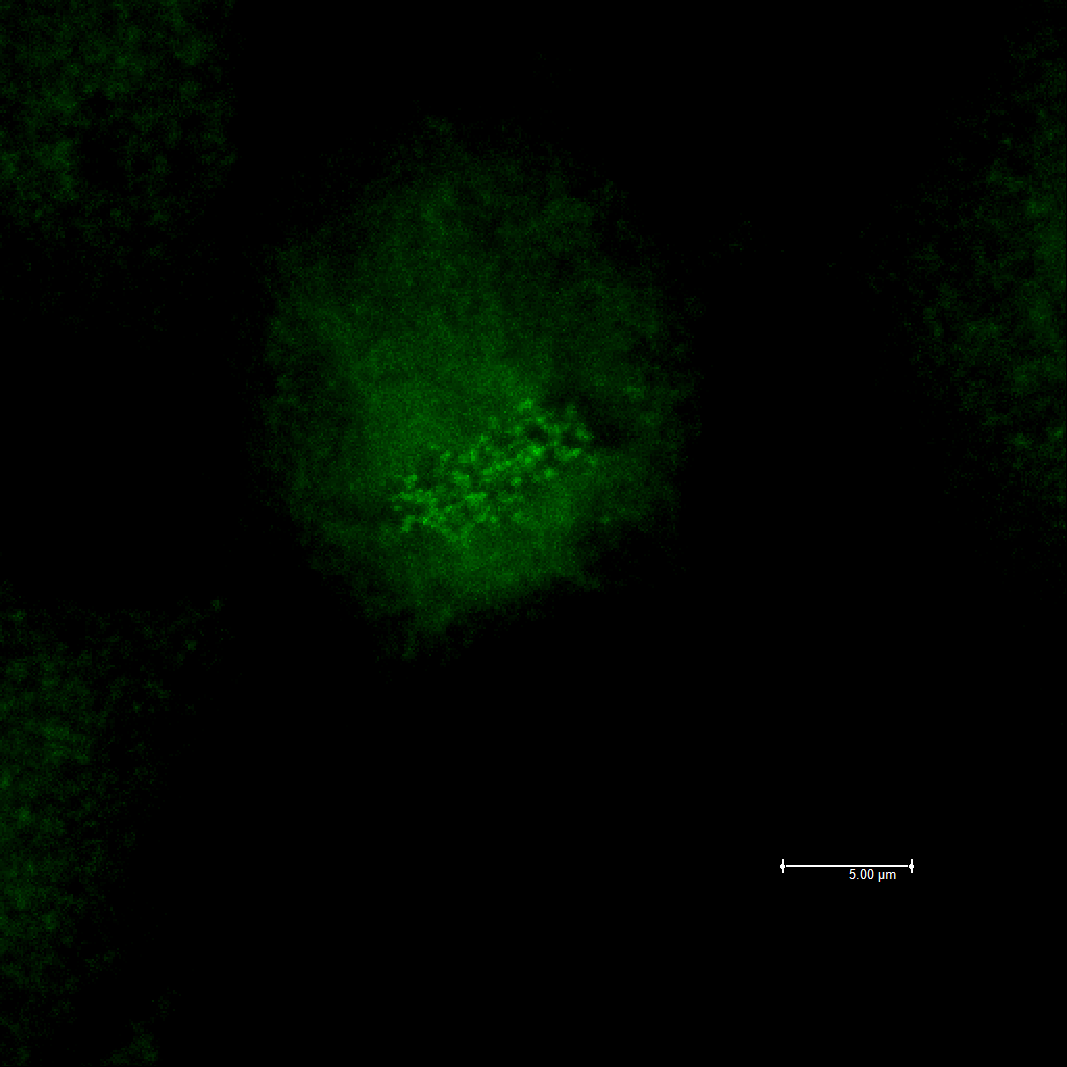

Supplement: Supplementary file 5 — Source Data Fig. 2 [file 44319_2024_106_MOESM5_ESM.zip › Figure 2/2C/Control esiRNA/ctrlesi_bubr1cy5_knl1568_n=1_121122.lif_Series010Snapshot3_ch00.tif]

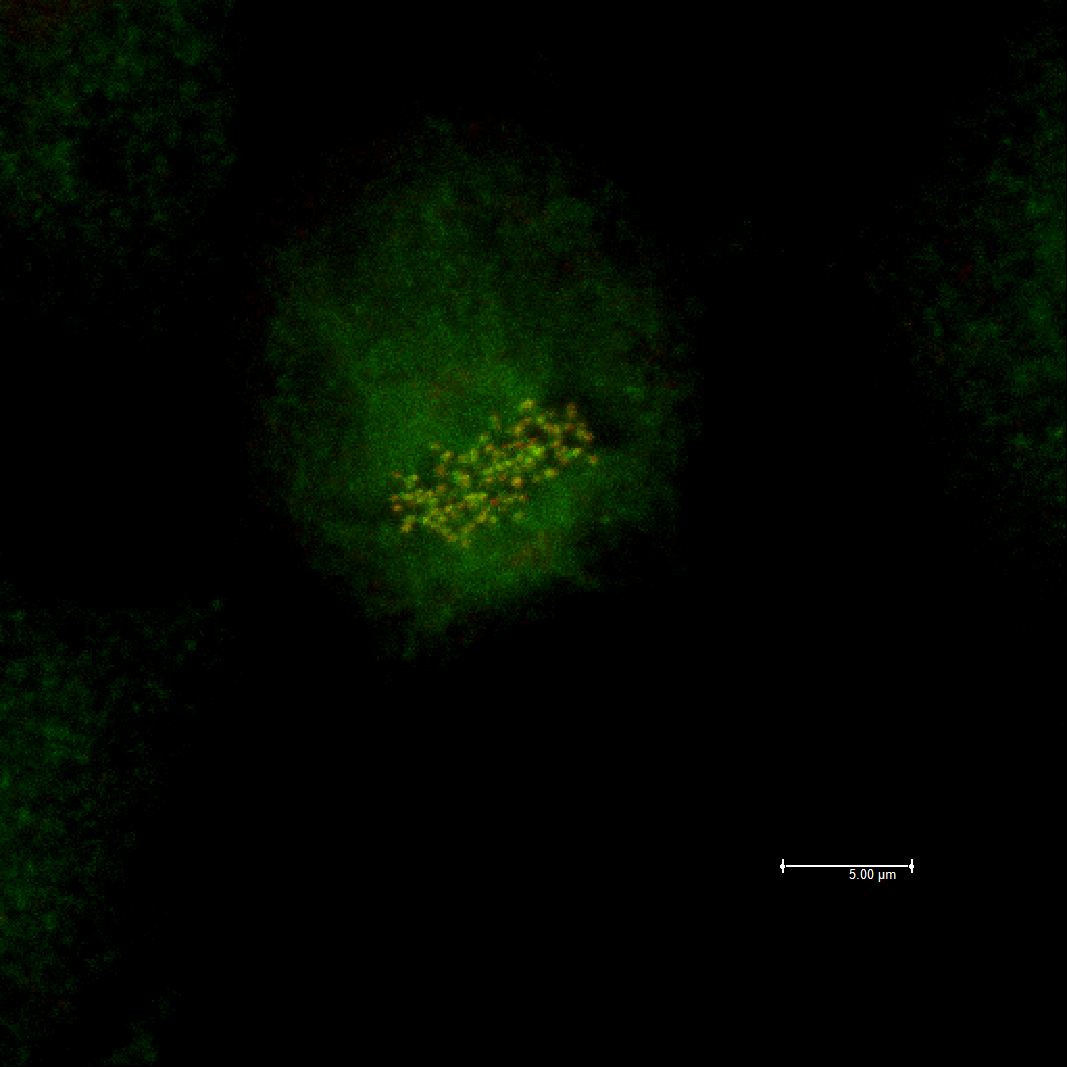

Supplement: Supplementary file 5 — Source Data Fig. 2 [file 44319_2024_106_MOESM5_ESM.zip › Figure 2/2C/Control esiRNA/ctrlesi_bubr1cy5_knl1568_n=1_121122.lif_Series010Snapshot4_ch00.tif]

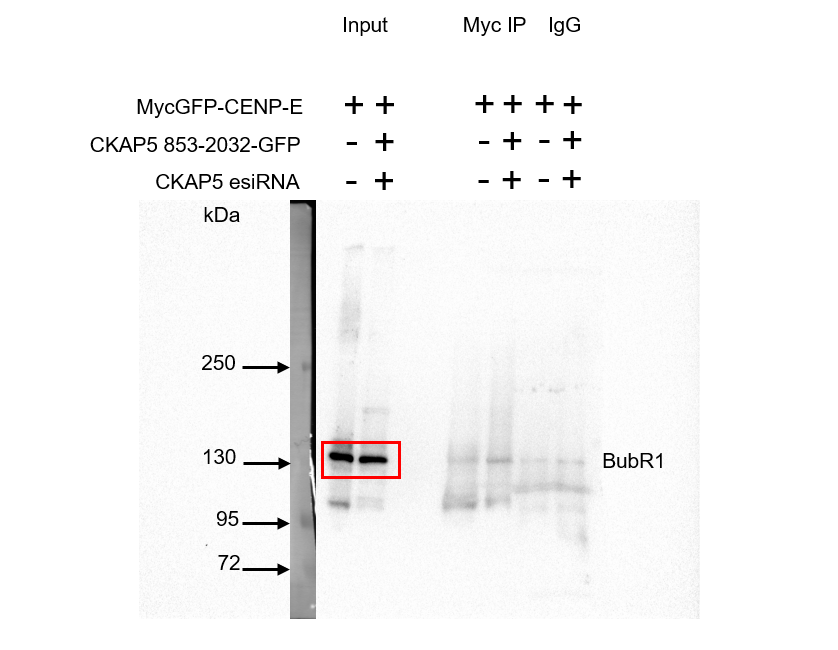

Supplement: Supplementary file 5 — Source Data Fig. 2 [file 44319_2024_106_MOESM5_ESM.zip › Figure 2/2E/BubR1/Input/Annotation.tif]

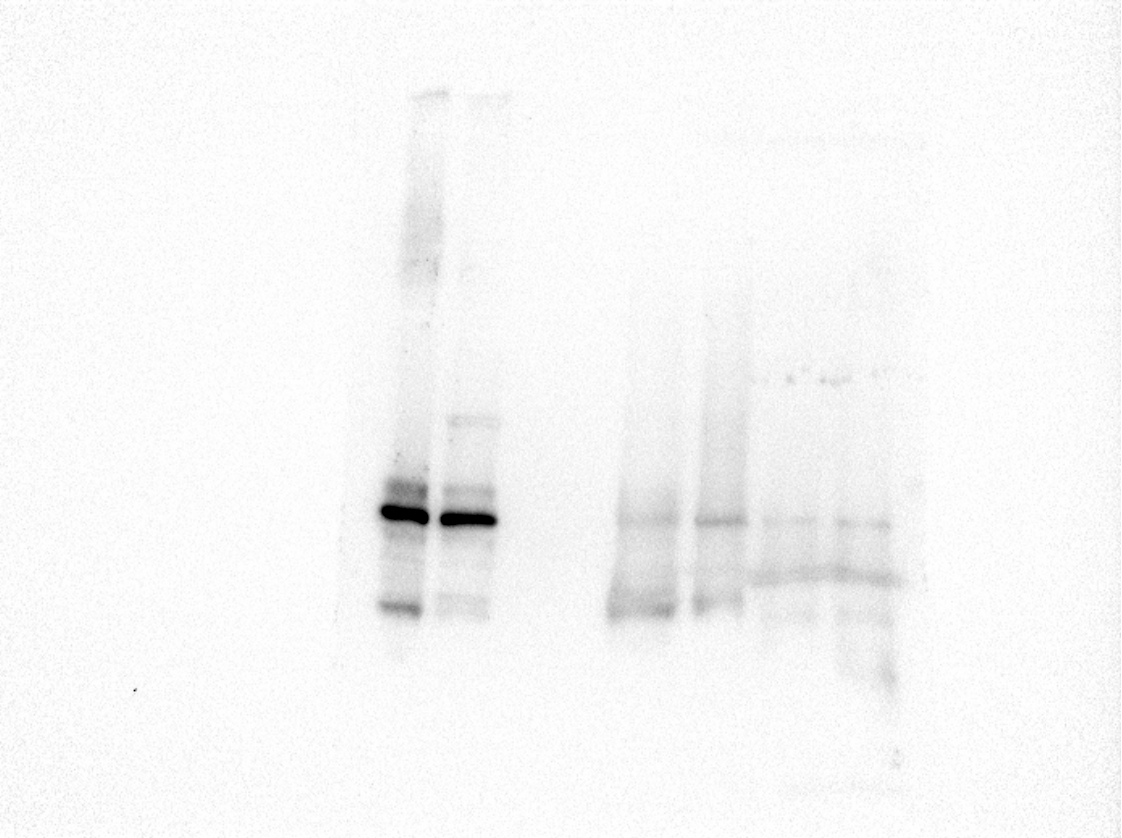

Supplement: Supplementary file 5 — Source Data Fig. 2 [file 44319_2024_106_MOESM5_ESM.zip › Figure 2/2E/BubR1/Input/bubr1 input.tif]

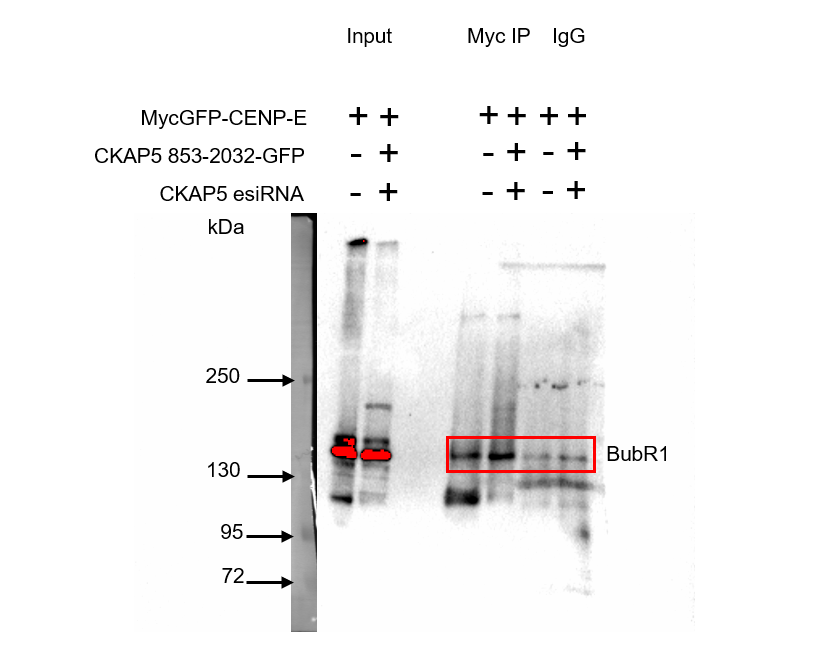

Supplement: Supplementary file 5 — Source Data Fig. 2 [file 44319_2024_106_MOESM5_ESM.zip › Figure 2/2E/BubR1/Myc IP/Annotation.tif]

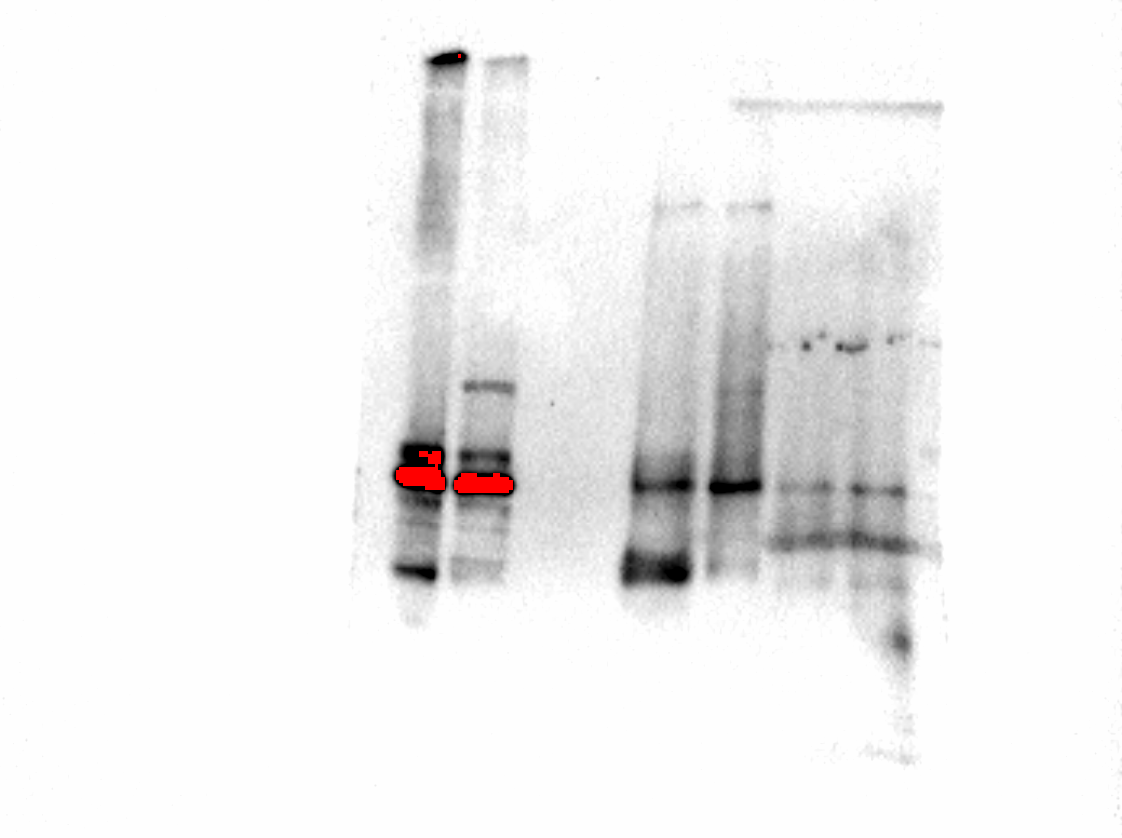

Supplement: Supplementary file 5 — Source Data Fig. 2 [file 44319_2024_106_MOESM5_ESM.zip › Figure 2/2E/BubR1/Myc IP/ip 5%.tif]

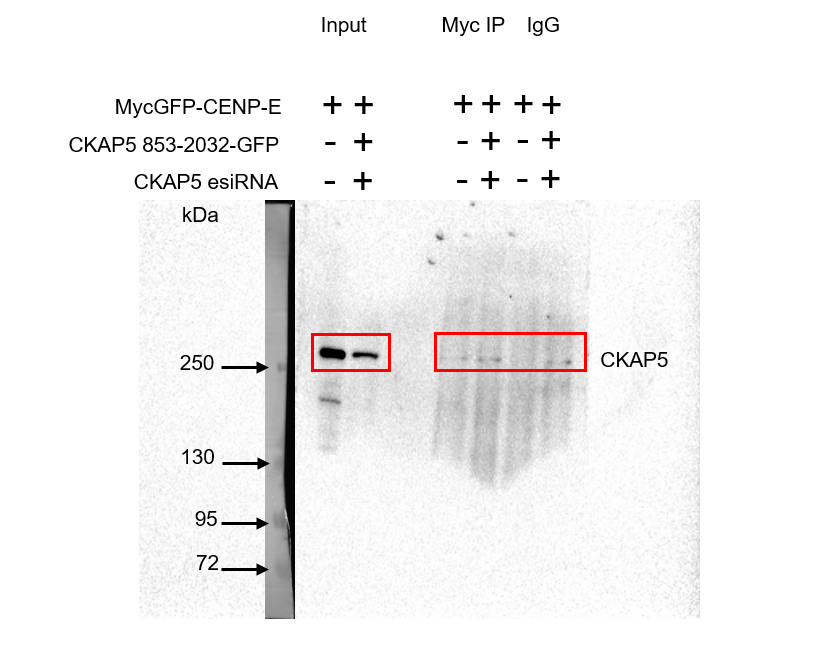

Supplement: Supplementary file 5 — Source Data Fig. 2 [file 44319_2024_106_MOESM5_ESM.zip › Figure 2/2E/CKAP5/Annotation.tif]

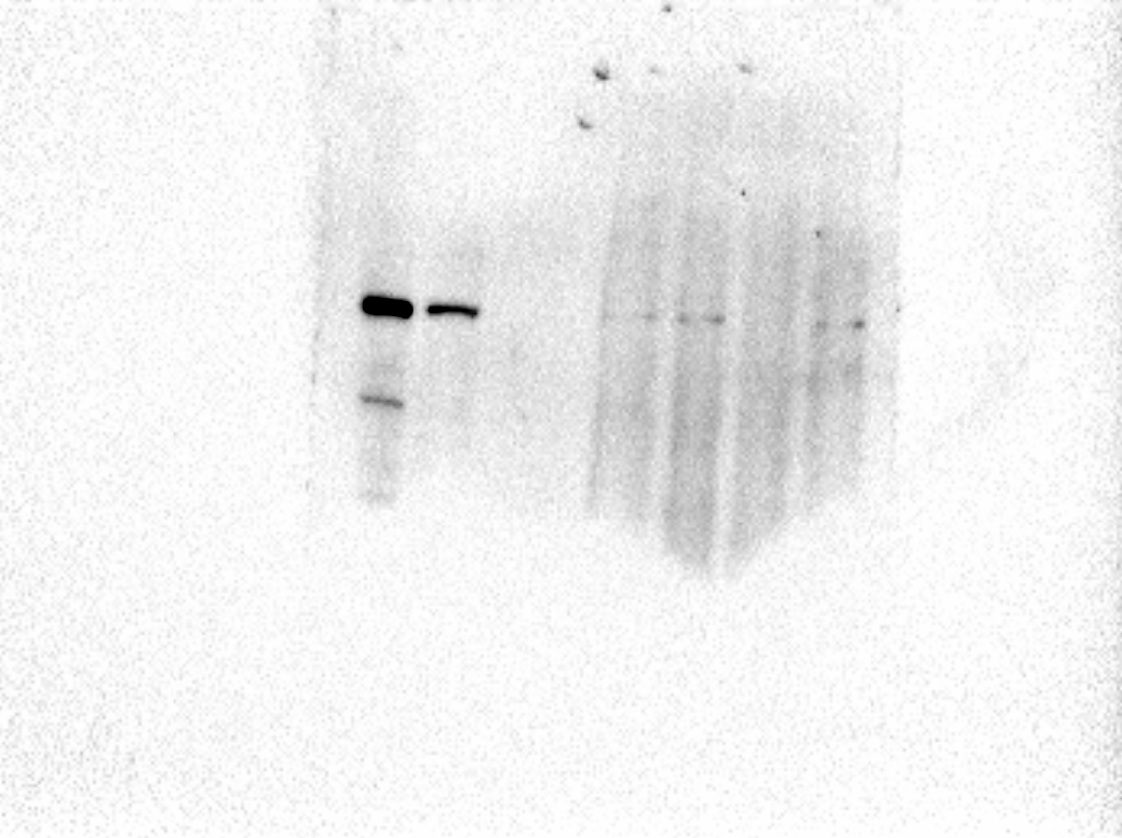

Supplement: Supplementary file 5 — Source Data Fig. 2 [file 44319_2024_106_MOESM5_ESM.zip › Figure 2/2E/CKAP5/INPUT IP.tif]

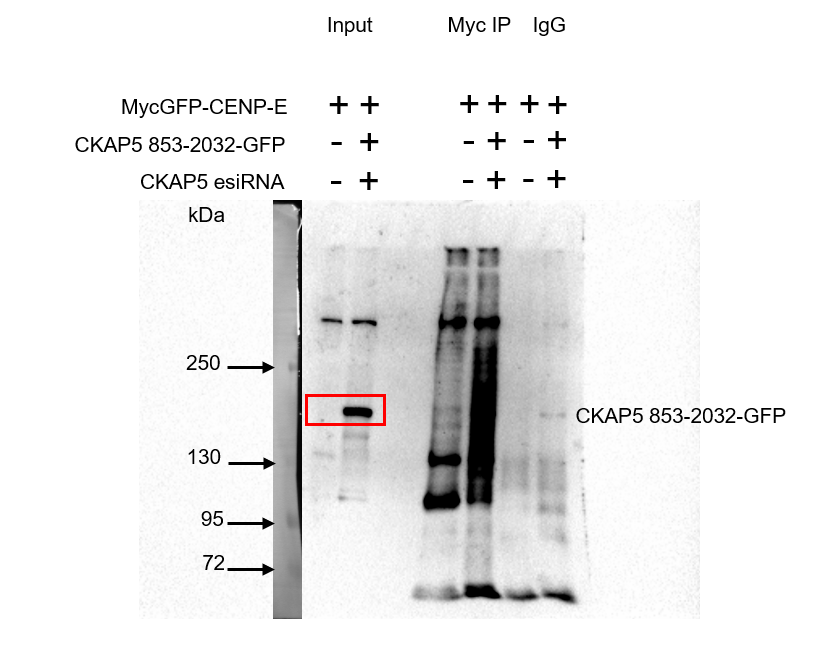

Supplement: Supplementary file 5 — Source Data Fig. 2 [file 44319_2024_106_MOESM5_ESM.zip › Figure 2/2E/CKAP5 853-2032-GFP/Input/Annotation.tif]

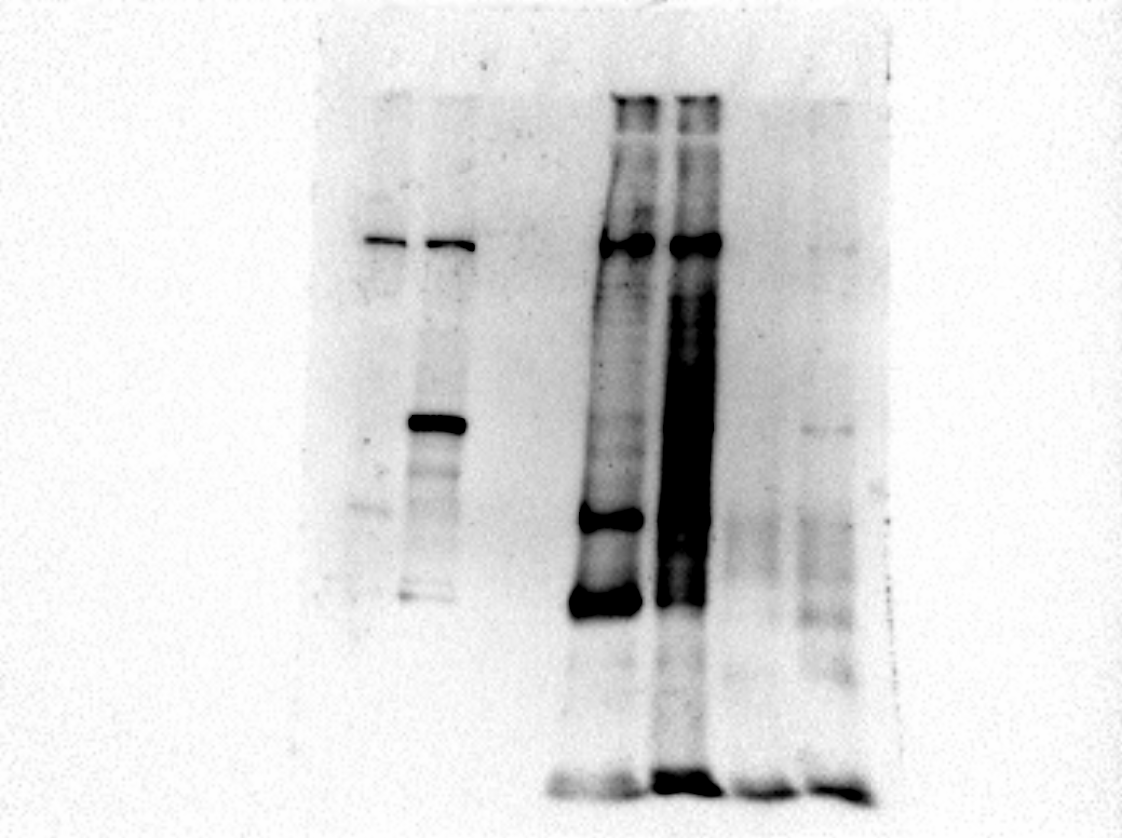

Supplement: Supplementary file 5 — Source Data Fig. 2 [file 44319_2024_106_MOESM5_ESM.zip › Figure 2/2E/CKAP5 853-2032-GFP/Input/Input_45ctd.tif]

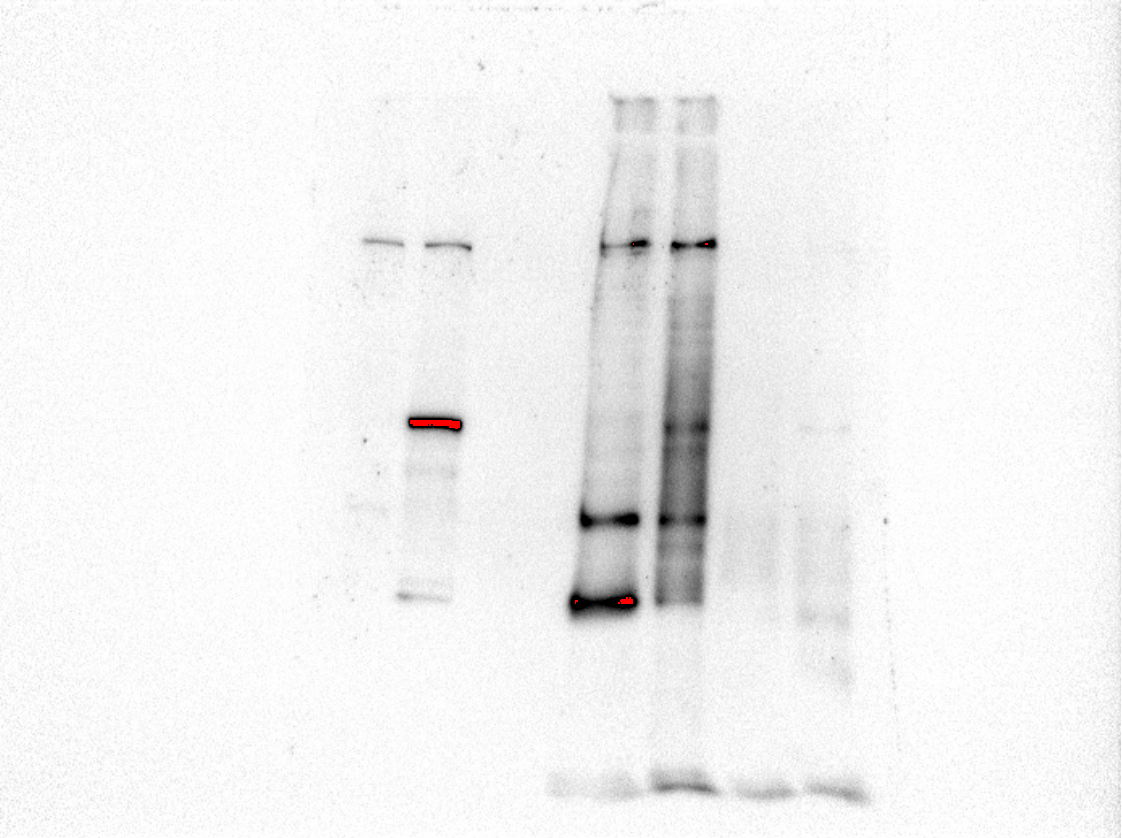

Supplement: Supplementary file 5 — Source Data Fig. 2 [file 44319_2024_106_MOESM5_ESM.zip › Figure 2/2E/CKAP5 853-2032-GFP/Myc IP/45CTD.tif]

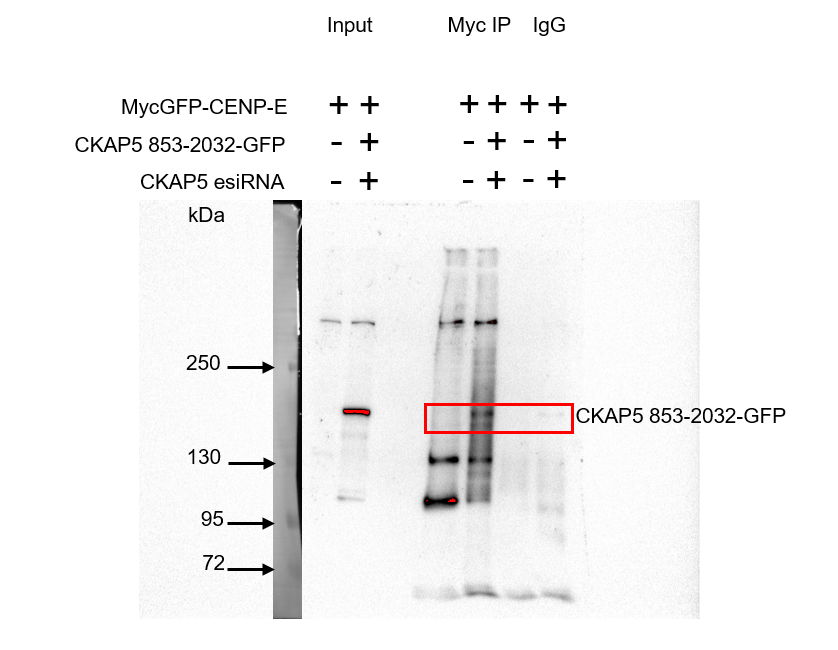

Supplement: Supplementary file 5 — Source Data Fig. 2 [file 44319_2024_106_MOESM5_ESM.zip › Figure 2/2E/CKAP5 853-2032-GFP/Myc IP/Annotation.tif]

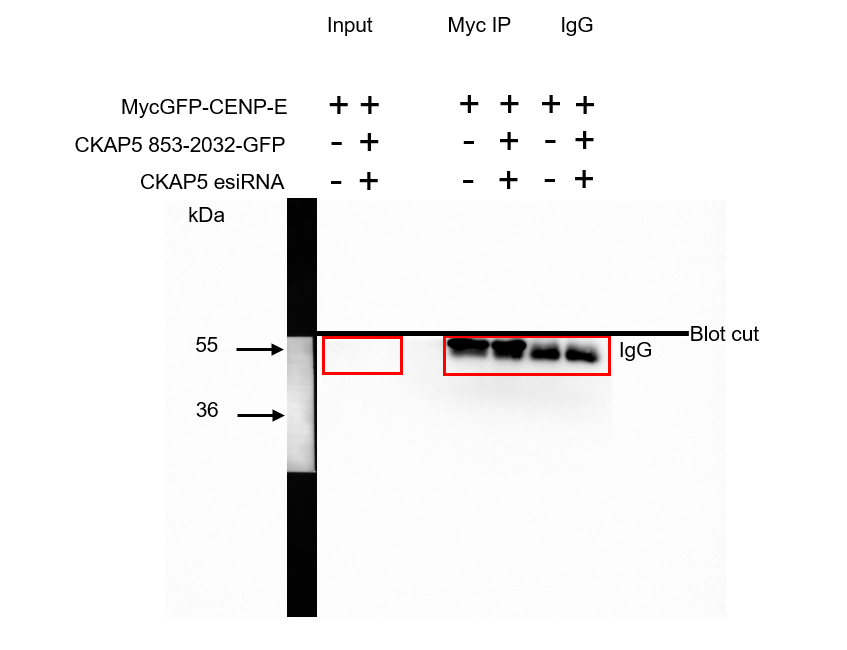

Supplement: Supplementary file 5 — Source Data Fig. 2 [file 44319_2024_106_MOESM5_ESM.zip › Figure 2/2E/IgG/Annotation.tif]

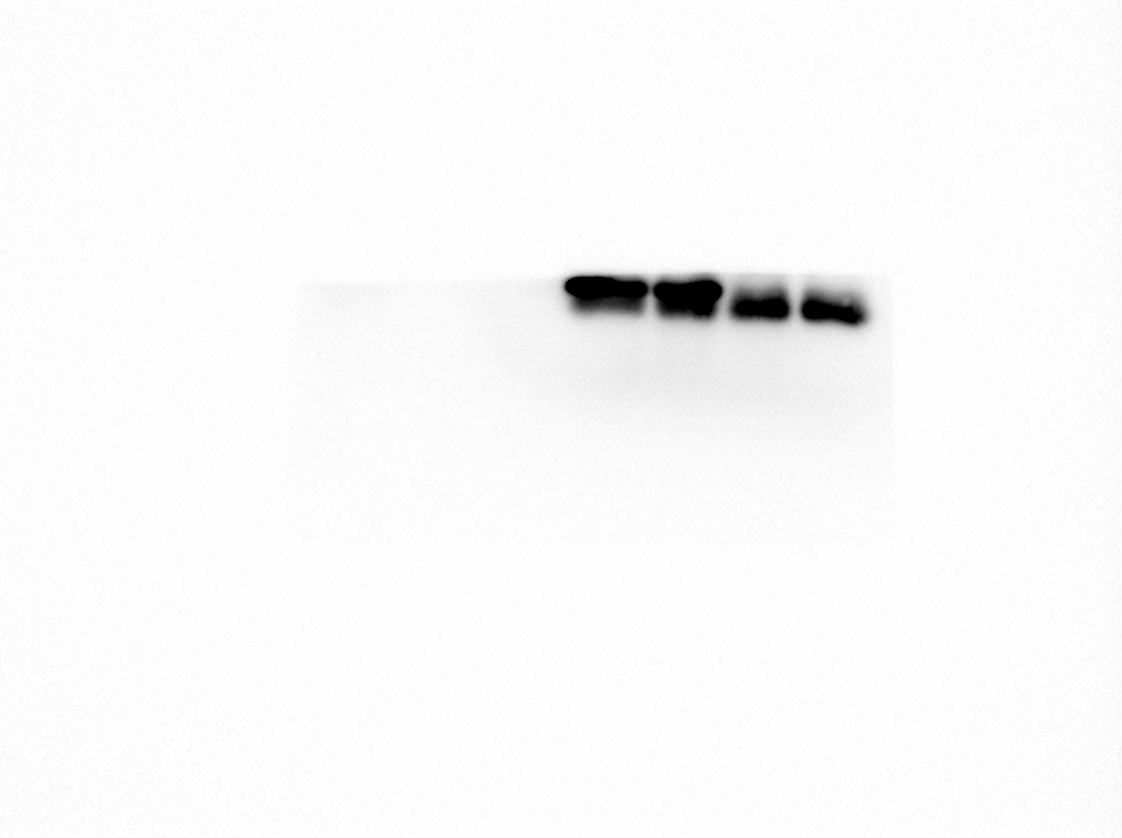

Supplement: Supplementary file 5 — Source Data Fig. 2 [file 44319_2024_106_MOESM5_ESM.zip › Figure 2/2E/IgG/IGG.tif]

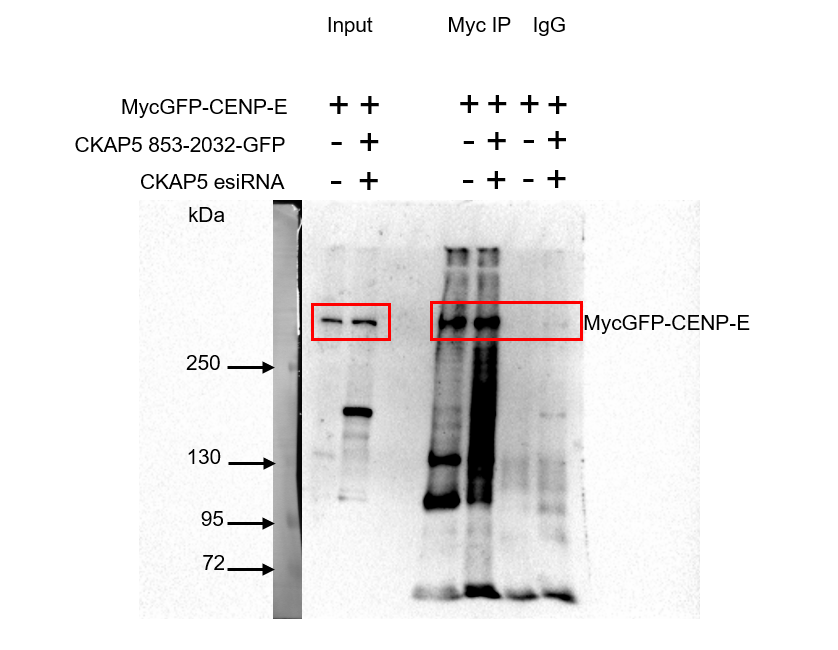

Supplement: Supplementary file 5 — Source Data Fig. 2 [file 44319_2024_106_MOESM5_ESM.zip › Figure 2/2E/MycGFP-CENP-E/Annotation.tif]

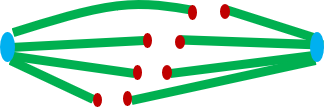

Supplement: Supplementary file 6 — Source Data Fig. 3 [file 44319_2024_106_MOESM6_ESM.zip › Figure 3/3A/CKAP5 esiRNA/Picture2.tif]

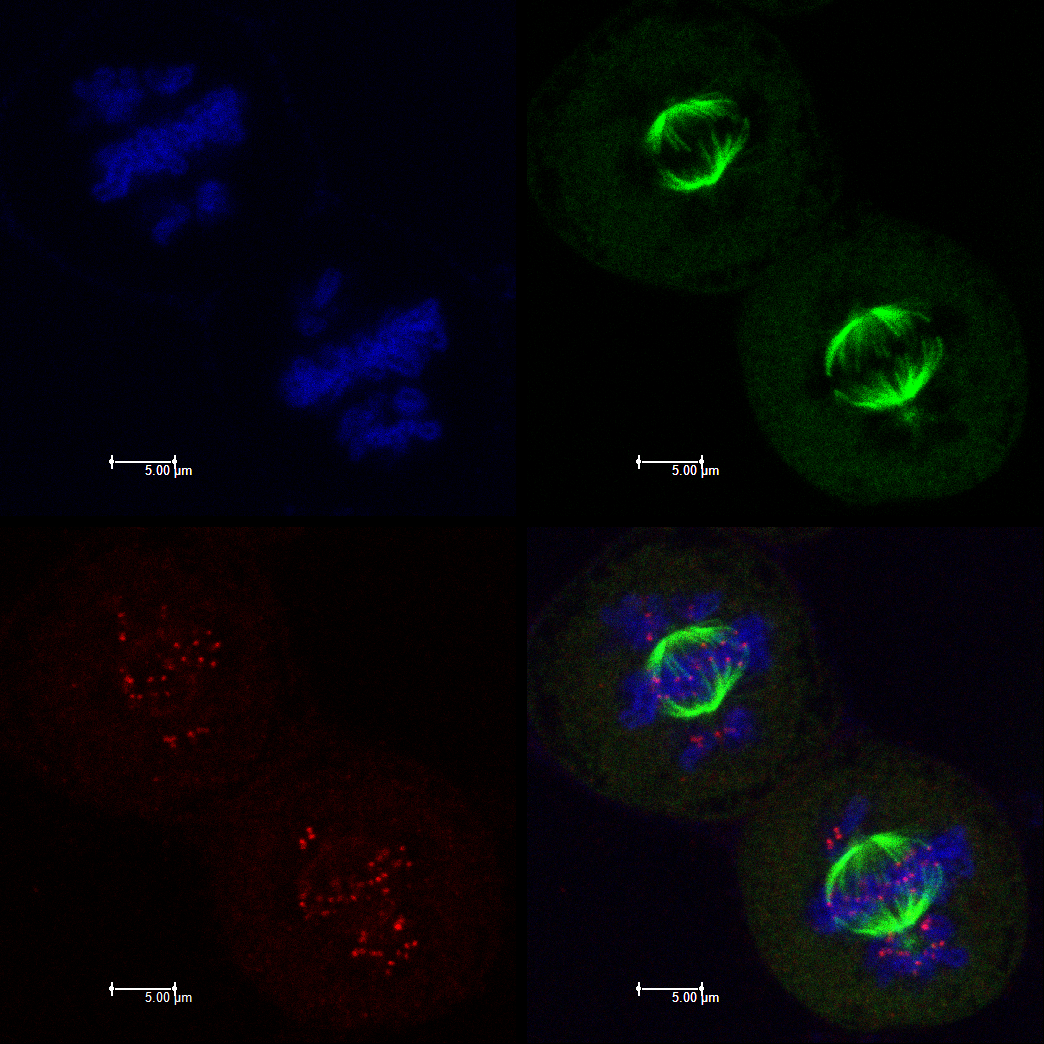

Supplement: Supplementary file 6 — Source Data Fig. 3 [file 44319_2024_106_MOESM6_ESM.zip › Figure 3/3A/CKAP5 esiRNA/togesi_240521.lif_Series012Snapshot All2.tif]

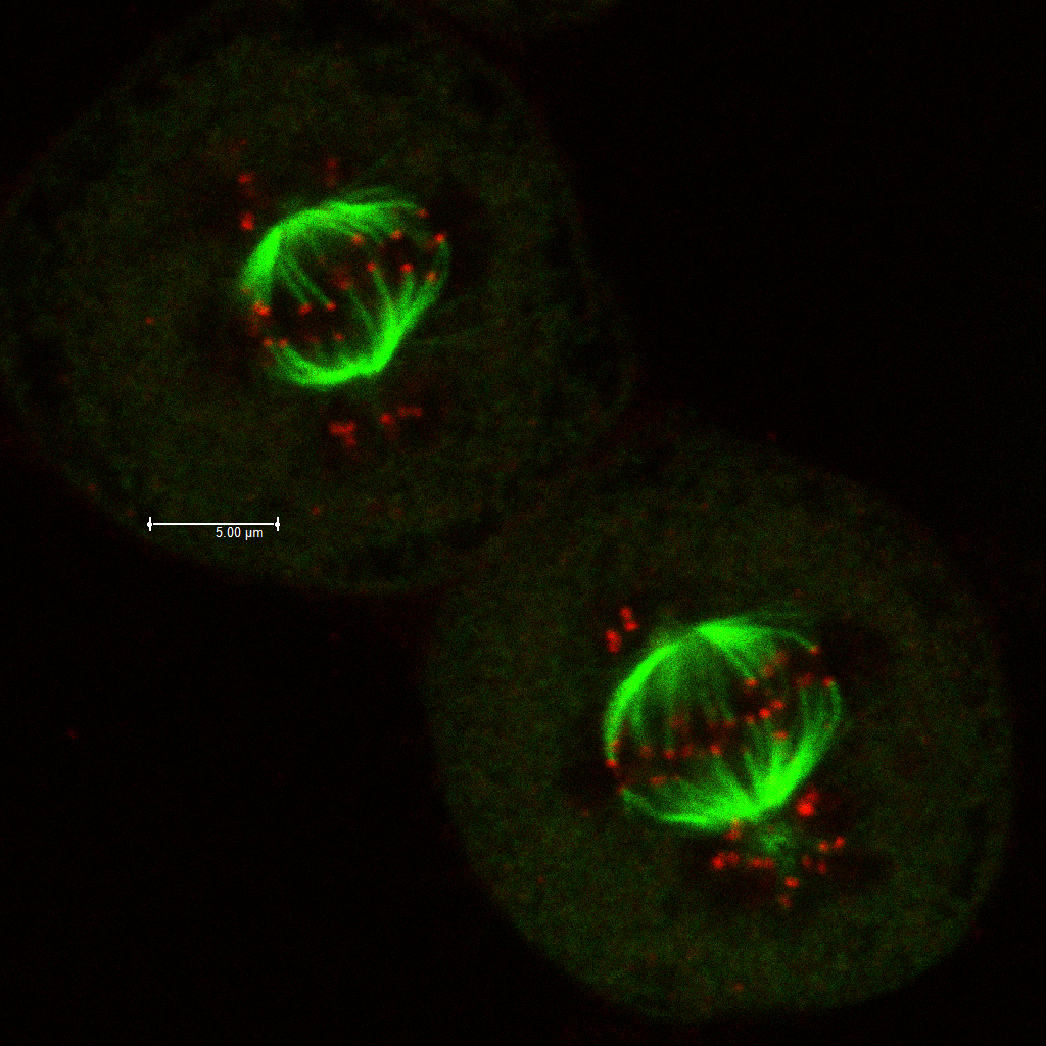

Supplement: Supplementary file 6 — Source Data Fig. 3 [file 44319_2024_106_MOESM6_ESM.zip › Figure 3/3A/CKAP5 esiRNA/togesi_240521.lif_Series012Snapshot4.tif]

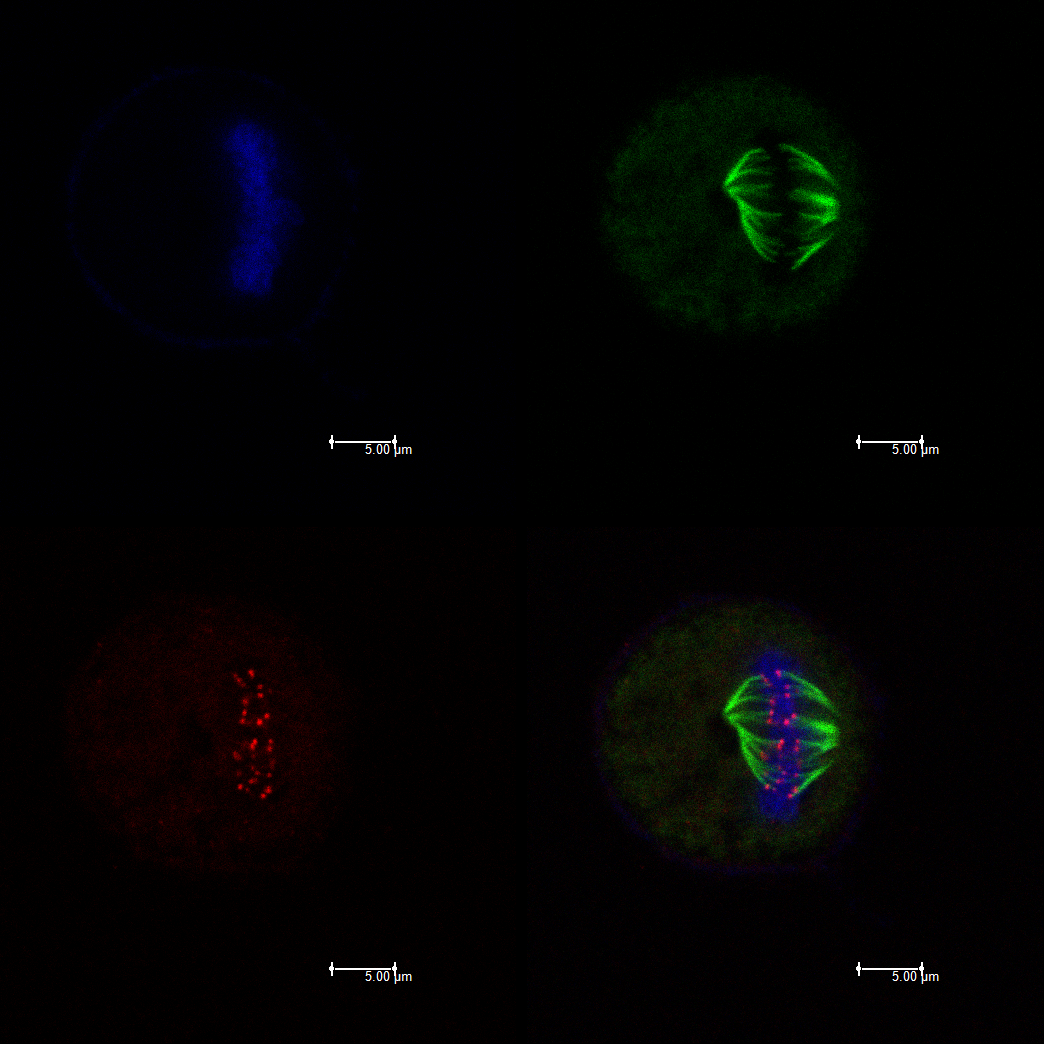

Supplement: Supplementary file 6 — Source Data Fig. 3 [file 44319_2024_106_MOESM6_ESM.zip › Figure 3/3A/Control esiRNA/ctrl_240521.lif_Series022Snapshot All1.tif]

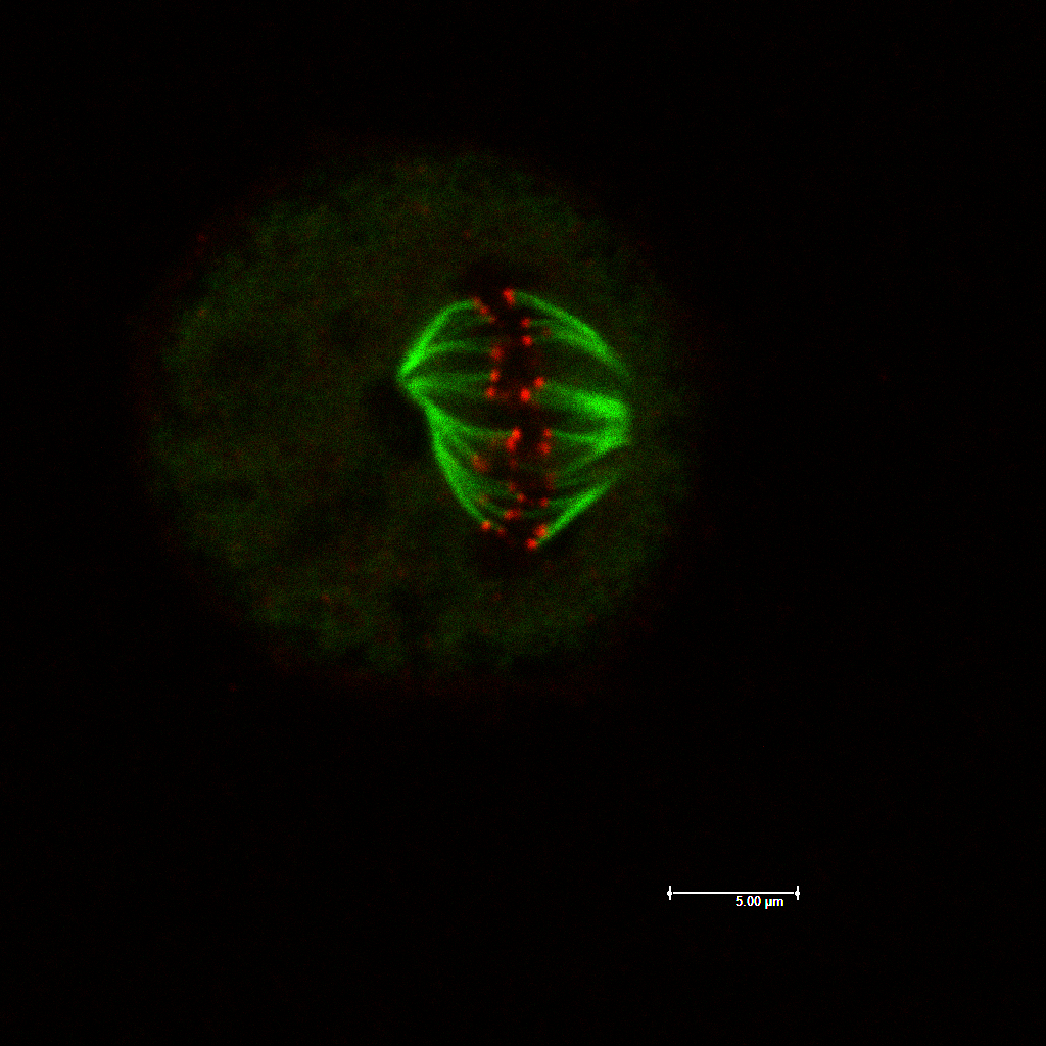

Supplement: Supplementary file 6 — Source Data Fig. 3 [file 44319_2024_106_MOESM6_ESM.zip › Figure 3/3A/Control esiRNA/ctrl_240521.lif_Series022Snapshot1.tif]

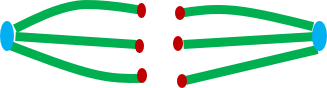

Supplement: Supplementary file 6 — Source Data Fig. 3 [file 44319_2024_106_MOESM6_ESM.zip › Figure 3/3A/Control esiRNA/Picture1.tif]

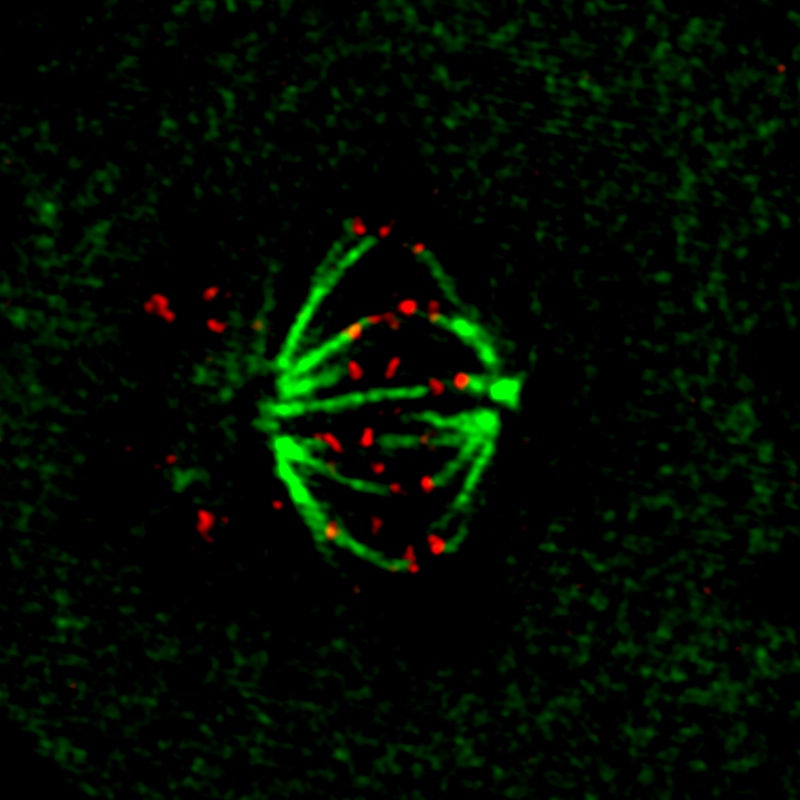

Supplement: Supplementary file 6 — Source Data Fig. 3 [file 44319_2024_106_MOESM6_ESM.zip › Figure 3/3D/Image 18_Structured Illumination.tif]

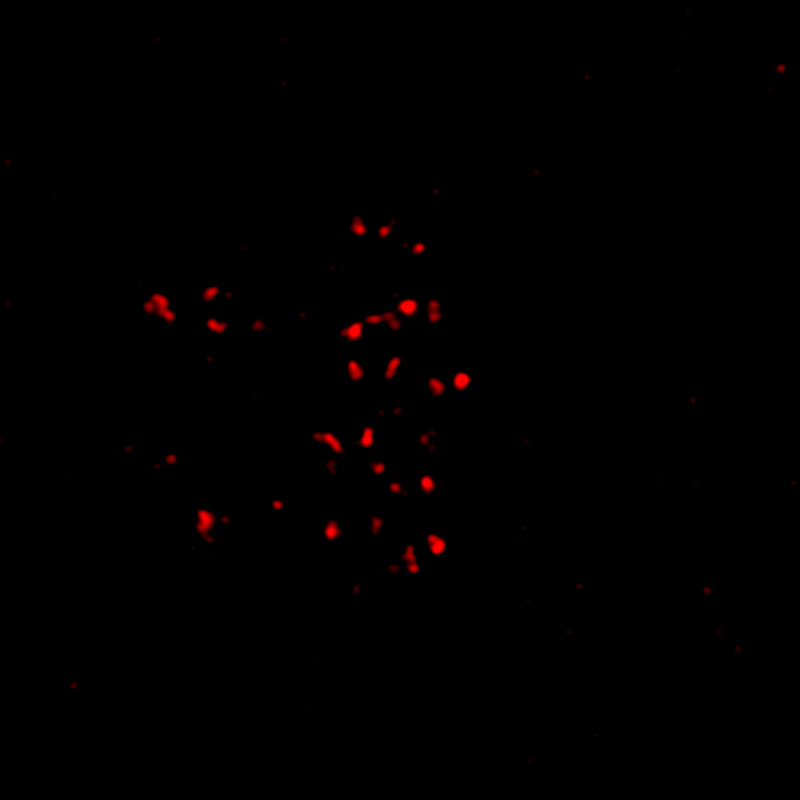

Supplement: Supplementary file 6 — Source Data Fig. 3 [file 44319_2024_106_MOESM6_ESM.zip › Figure 3/3D/Image 18_Structured Illumination.tif1.tif]

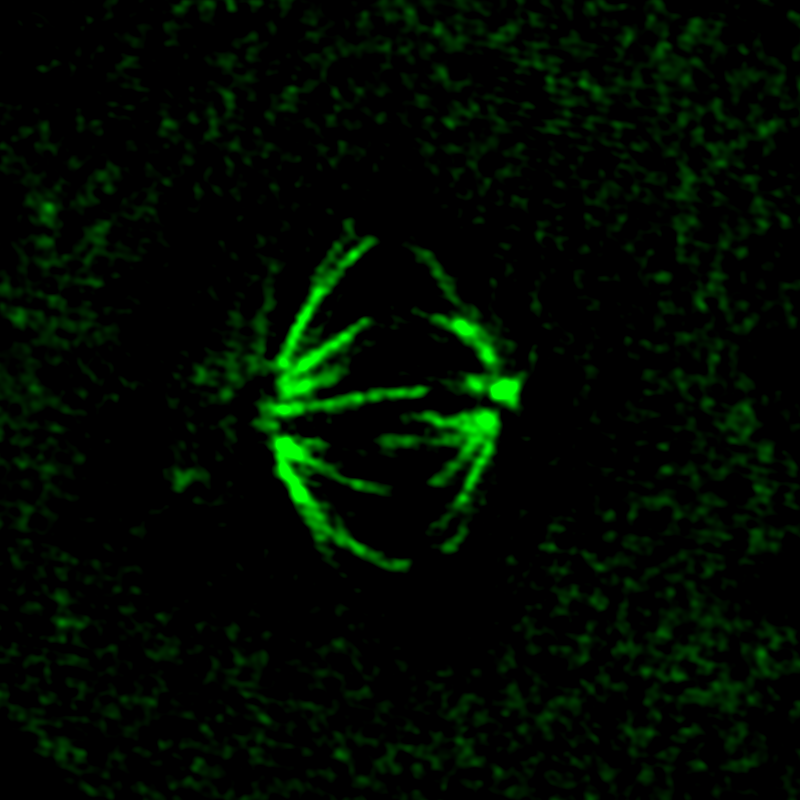

Supplement: Supplementary file 6 — Source Data Fig. 3 [file 44319_2024_106_MOESM6_ESM.zip › Figure 3/3D/Image 18_Structured Illumination.tif2.tif]

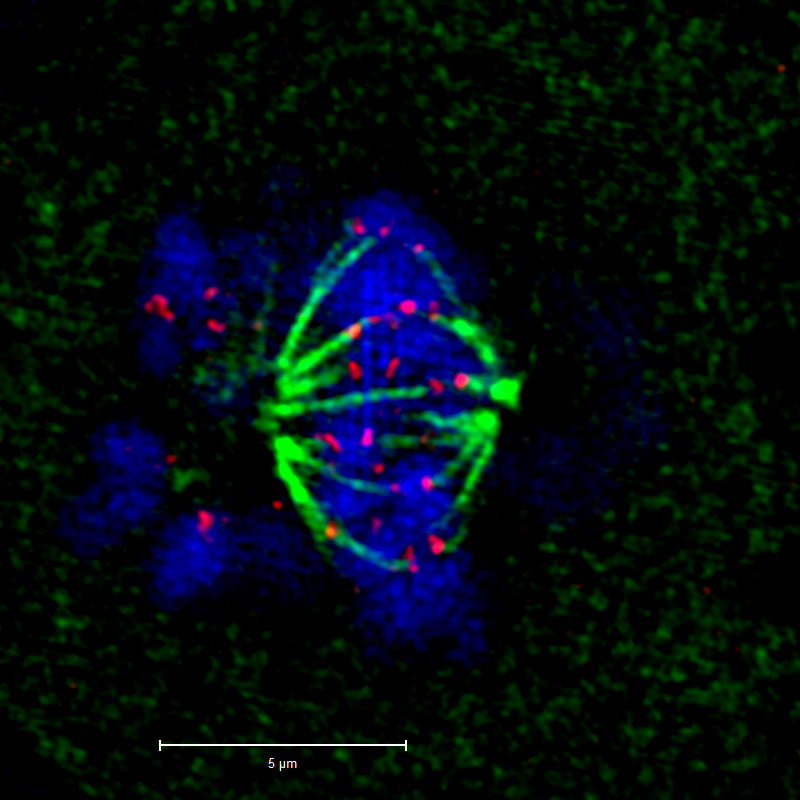

Supplement: Supplementary file 6 — Source Data Fig. 3 [file 44319_2024_106_MOESM6_ESM.zip › Figure 3/3D/Image 18_Structured Illumination.tif3.tif]

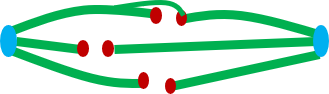

Supplement: Supplementary file 6 — Source Data Fig. 3 [file 44319_2024_106_MOESM6_ESM.zip › Figure 3/3D/MERO CARTOON.tif]

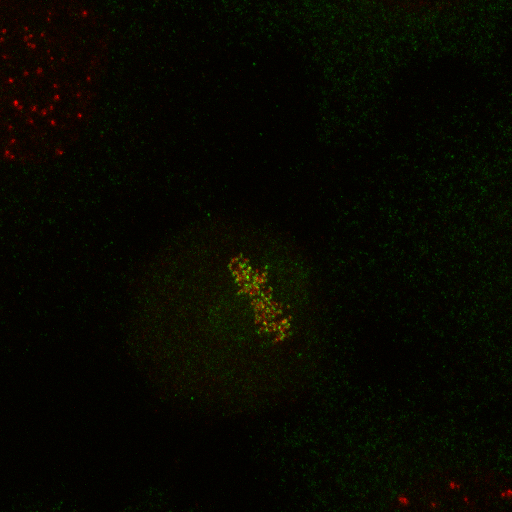

Supplement: Supplementary file 6 — Source Data Fig. 3 [file 44319_2024_106_MOESM6_ESM.zip › Figure 3/3H/Control + DMSO/Composite (RGB).tif]

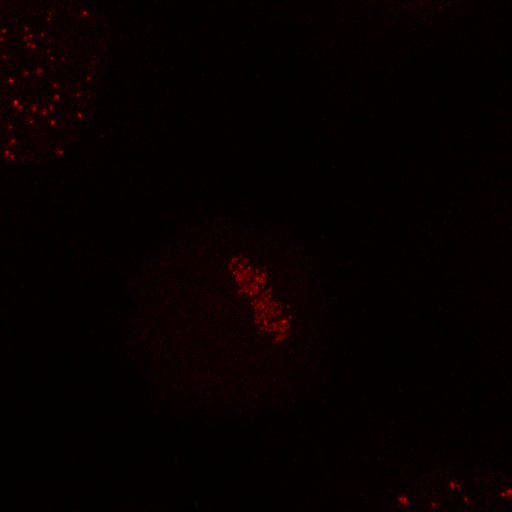

Supplement: Supplementary file 6 — Source Data Fig. 3 [file 44319_2024_106_MOESM6_ESM.zip › Figure 3/3H/Control + DMSO/MAX_Image 51.czi - C=0-1.tif]

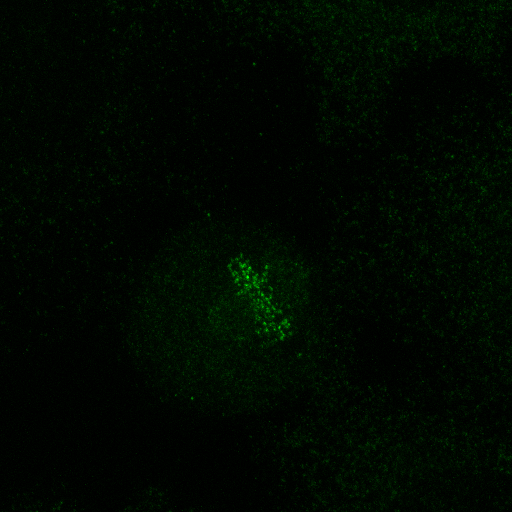

Supplement: Supplementary file 6 — Source Data Fig. 3 [file 44319_2024_106_MOESM6_ESM.zip › Figure 3/3H/Control + DMSO/MAX_Image 51.czi - C=1-1.tif]

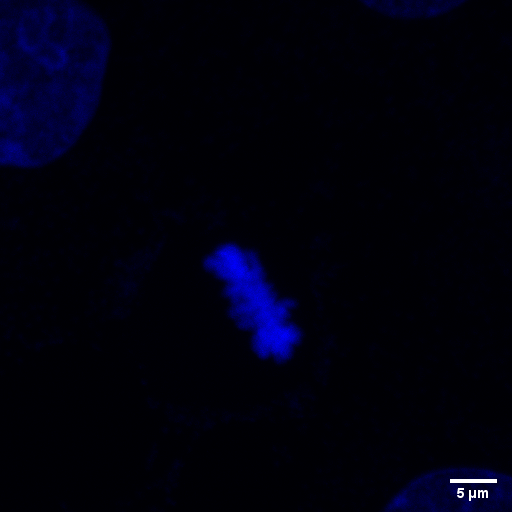

Supplement: Supplementary file 6 — Source Data Fig. 3 [file 44319_2024_106_MOESM6_ESM.zip › Figure 3/3H/Control + DMSO/MAX_Image 51.czi - C=2-1.tif]

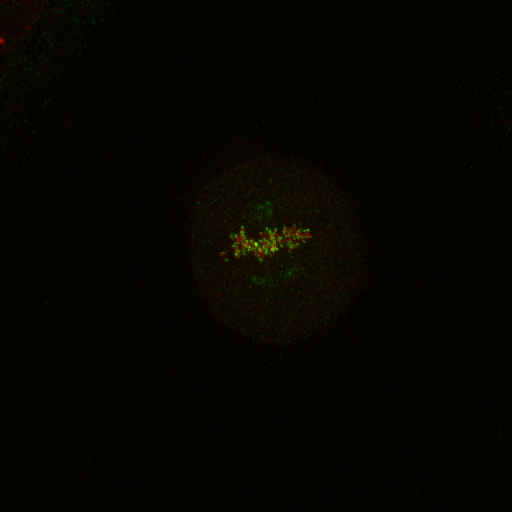

Supplement: Supplementary file 6 — Source Data Fig. 3 [file 44319_2024_106_MOESM6_ESM.zip › Figure 3/3H/Control + Paclitaxel/Composite (RGB).tif]

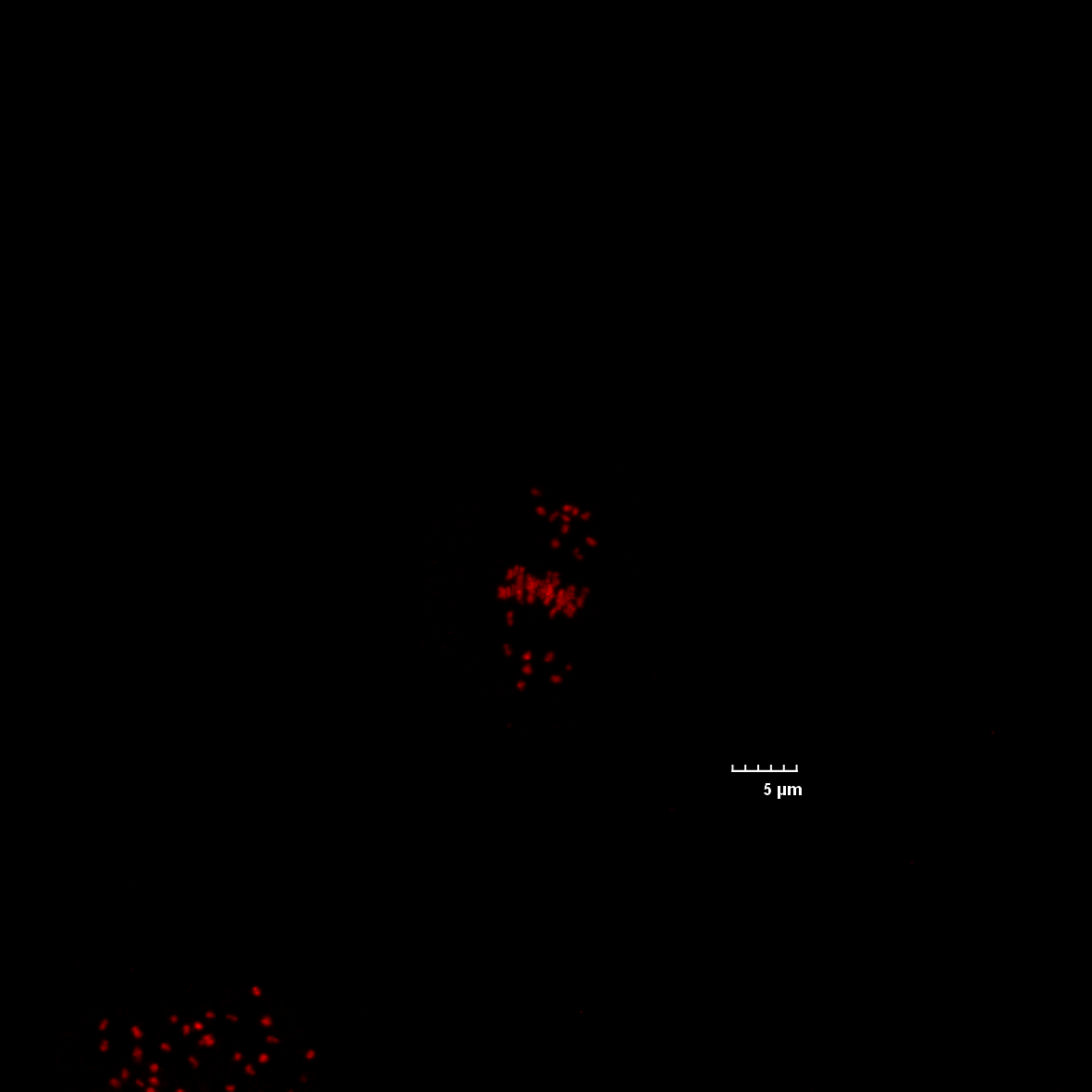

Supplement: Supplementary file 6 — Source Data Fig. 3 [file 44319_2024_106_MOESM6_ESM.zip › Figure 3/3J/CKAP5 esiRNA/aca.tif]

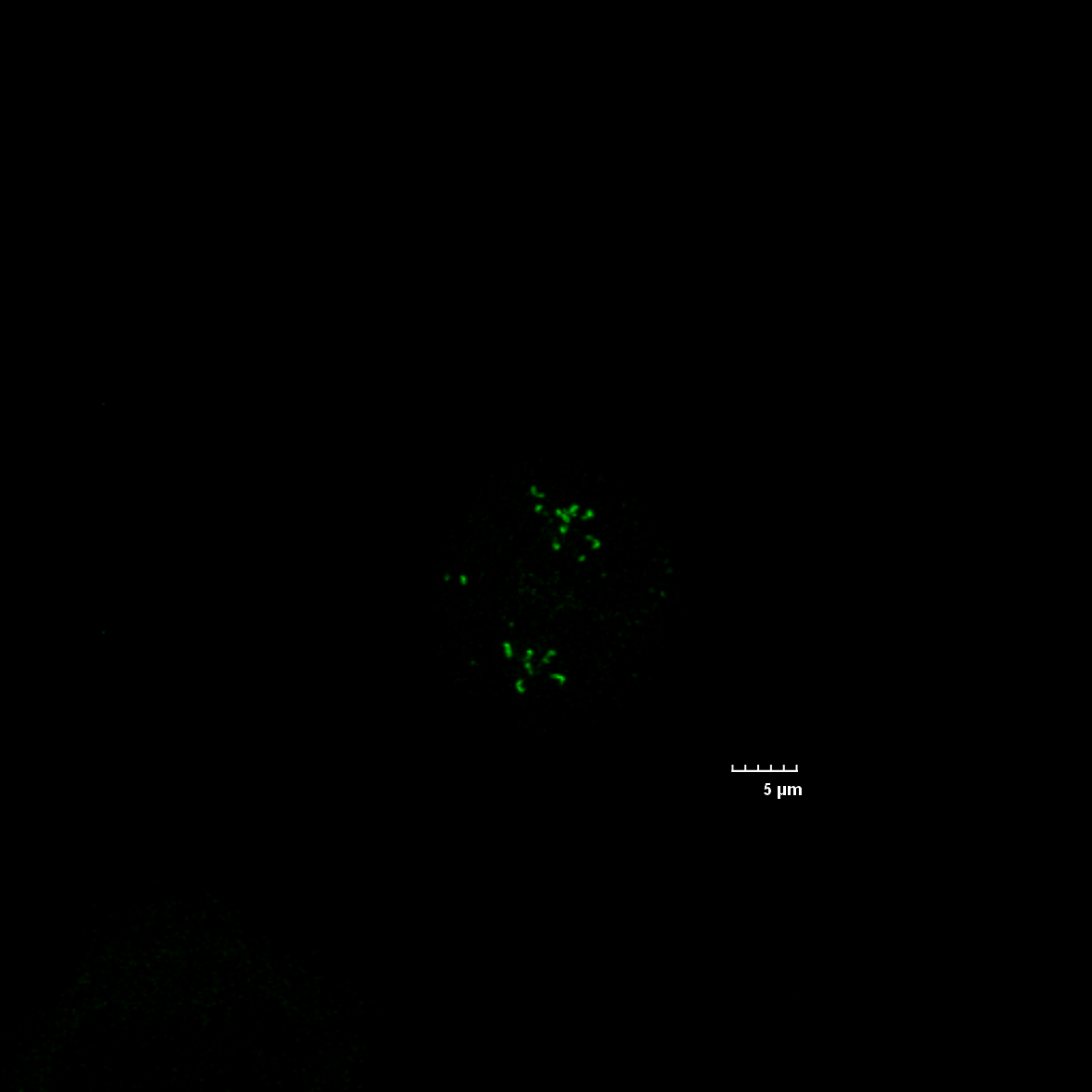

Supplement: Supplementary file 6 — Source Data Fig. 3 [file 44319_2024_106_MOESM6_ESM.zip › Figure 3/3J/CKAP5 esiRNA/cenpe.tif]

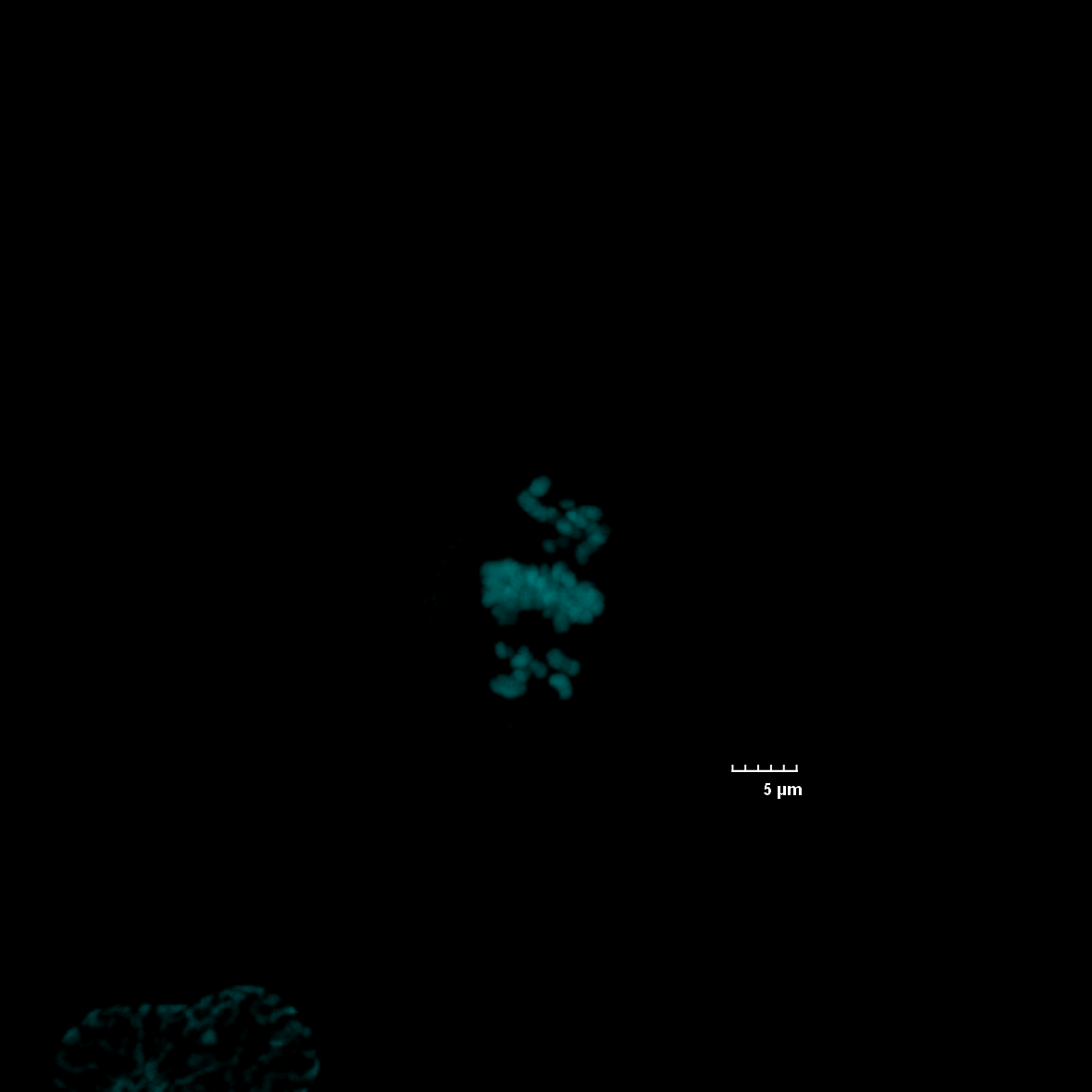

Supplement: Supplementary file 6 — Source Data Fig. 3 [file 44319_2024_106_MOESM6_ESM.zip › Figure 3/3J/CKAP5 esiRNA/dapi.tif]

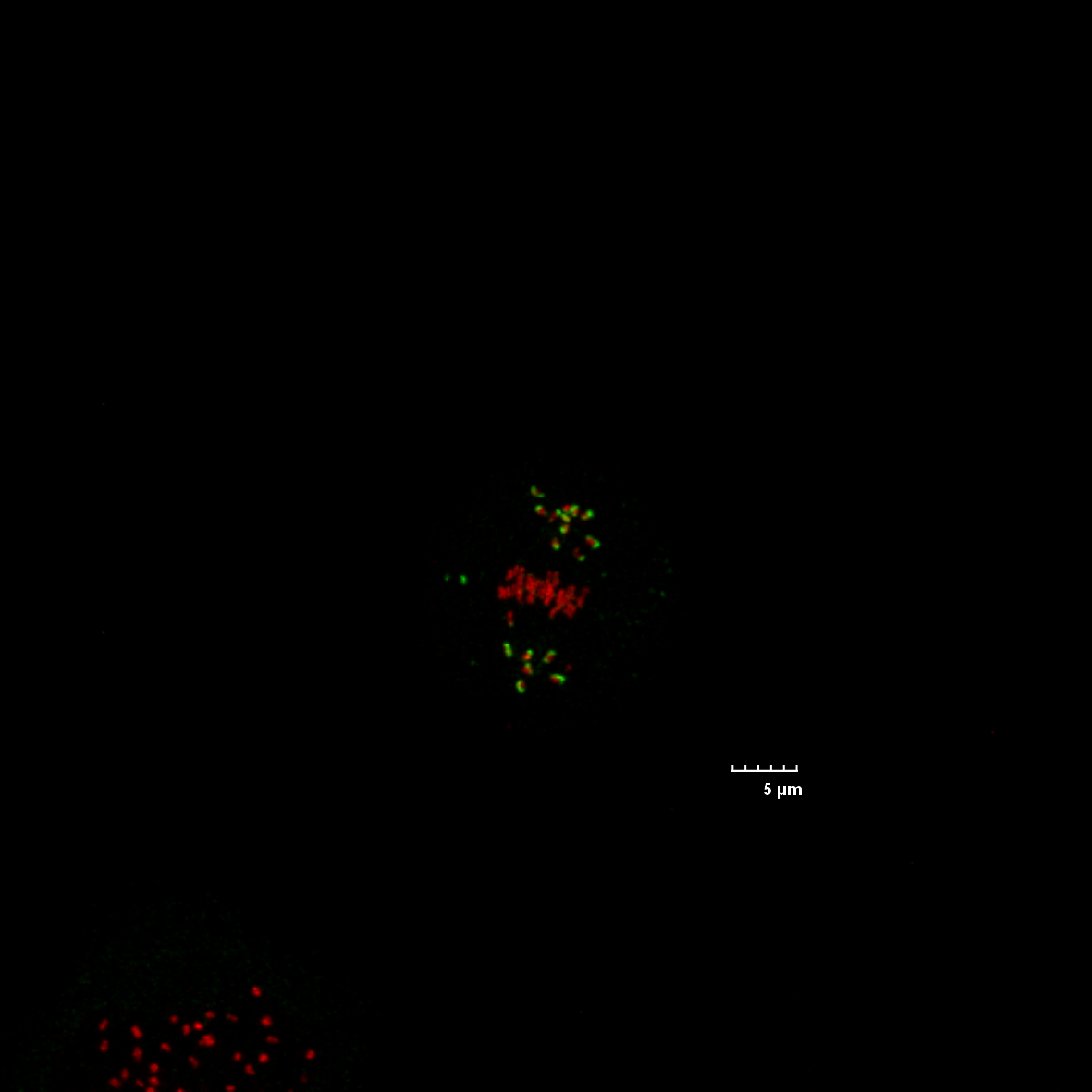

Supplement: Supplementary file 6 — Source Data Fig. 3 [file 44319_2024_106_MOESM6_ESM.zip › Figure 3/3J/CKAP5 esiRNA/merge.tif]

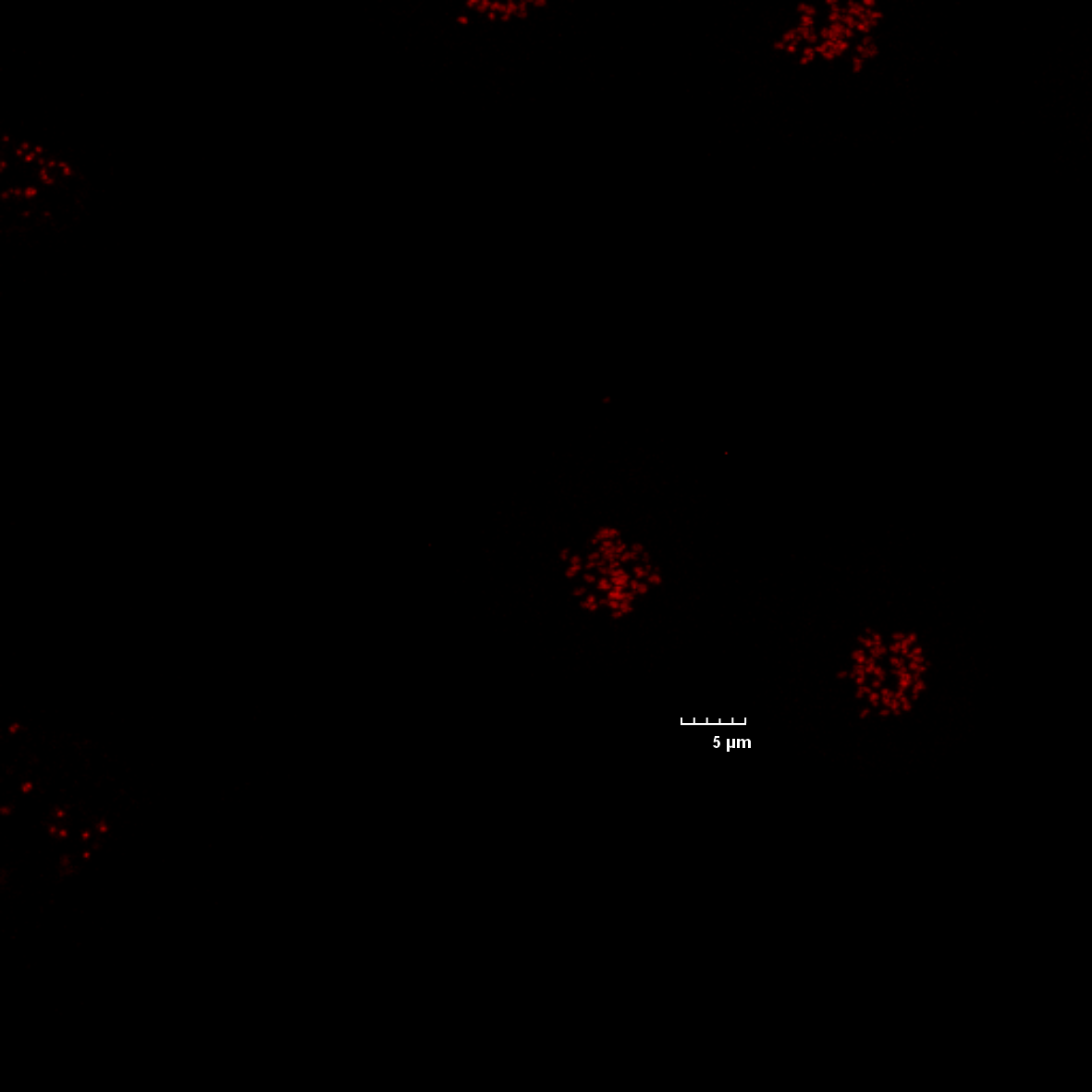

Supplement: Supplementary file 6 — Source Data Fig. 3 [file 44319_2024_106_MOESM6_ESM.zip › Figure 3/3J/CKAP5 esiRNA+partial Noco/aca.tif]

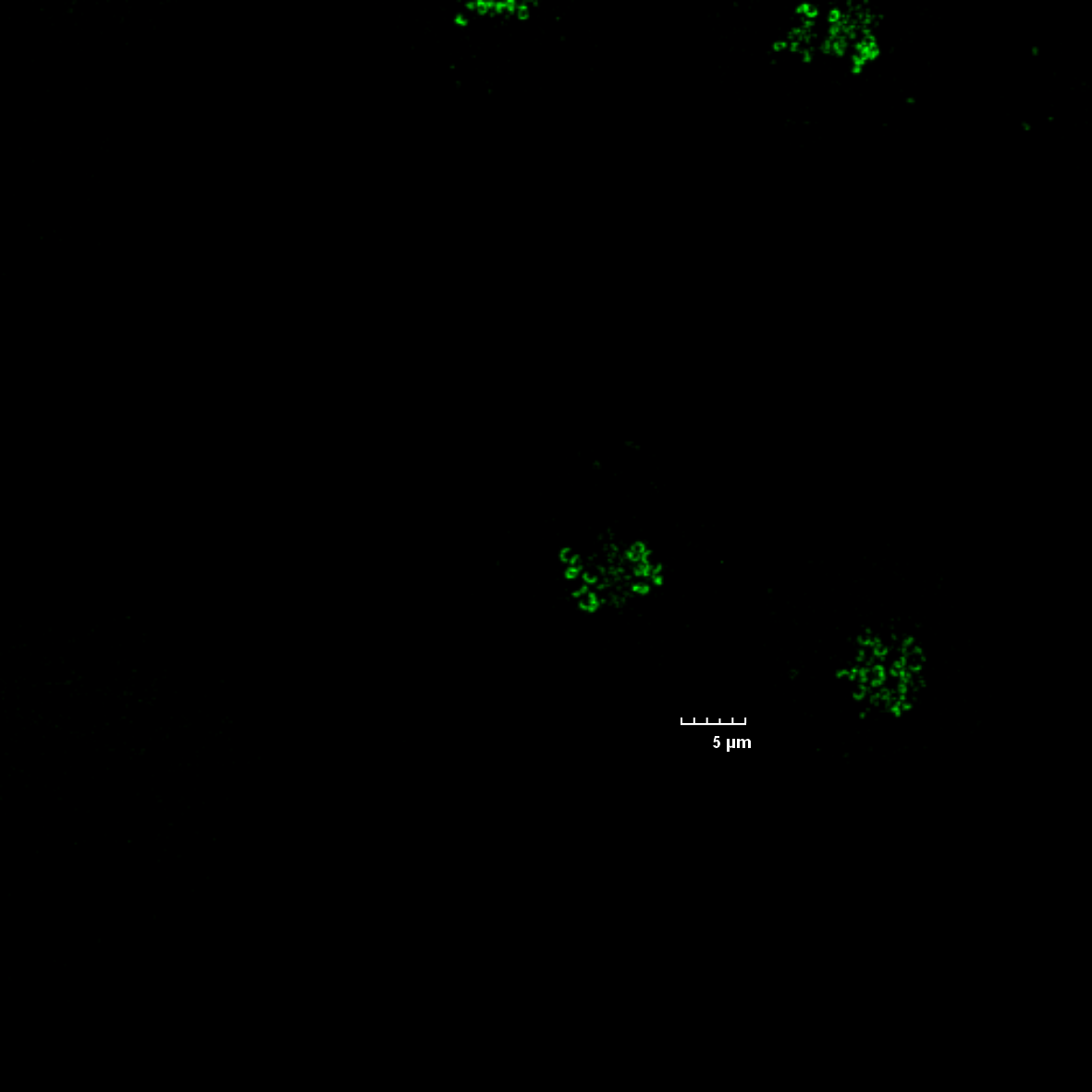

Supplement: Supplementary file 6 — Source Data Fig. 3 [file 44319_2024_106_MOESM6_ESM.zip › Figure 3/3J/CKAP5 esiRNA+partial Noco/cenpe.tif]

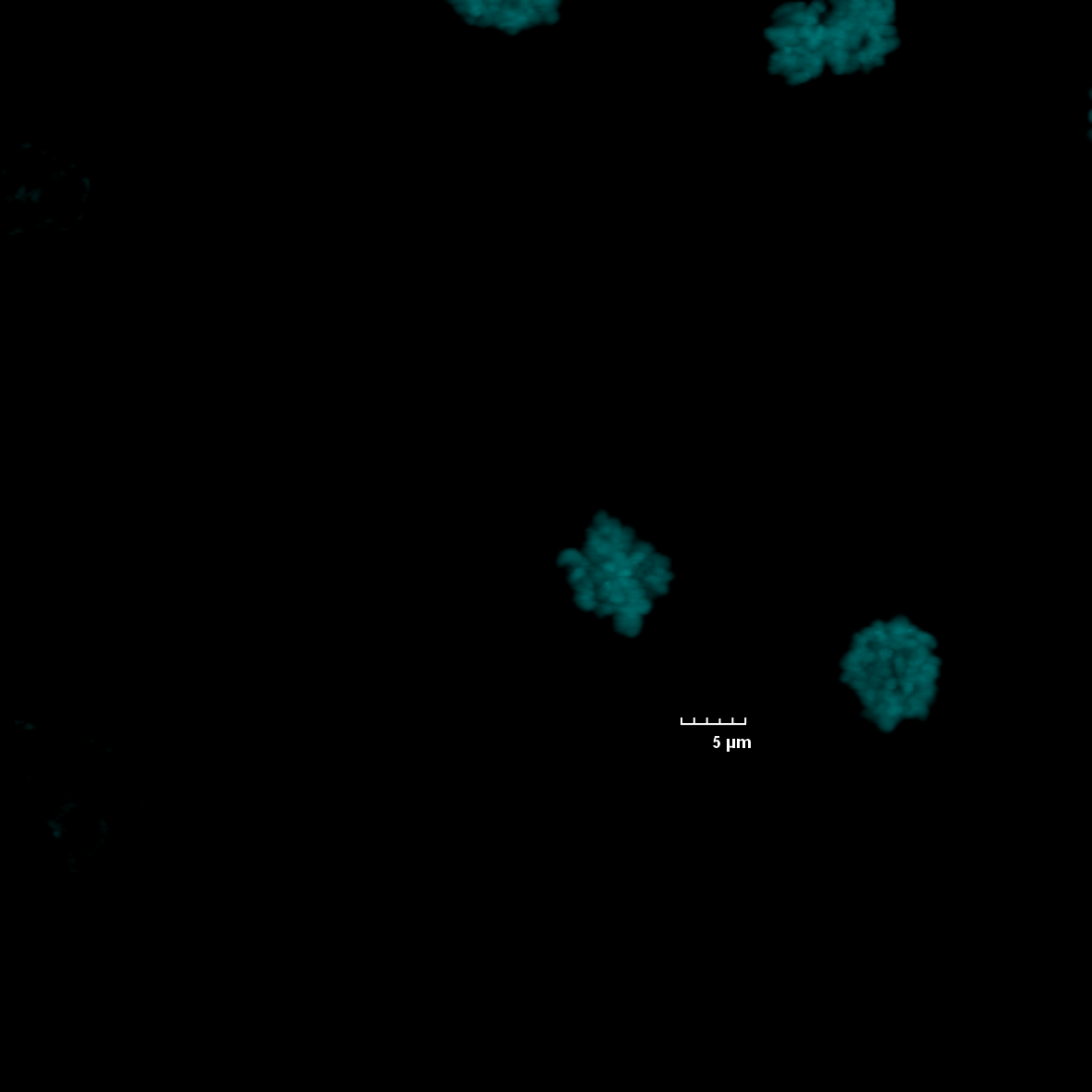

Supplement: Supplementary file 6 — Source Data Fig. 3 [file 44319_2024_106_MOESM6_ESM.zip › Figure 3/3J/CKAP5 esiRNA+partial Noco/dapi.tif]

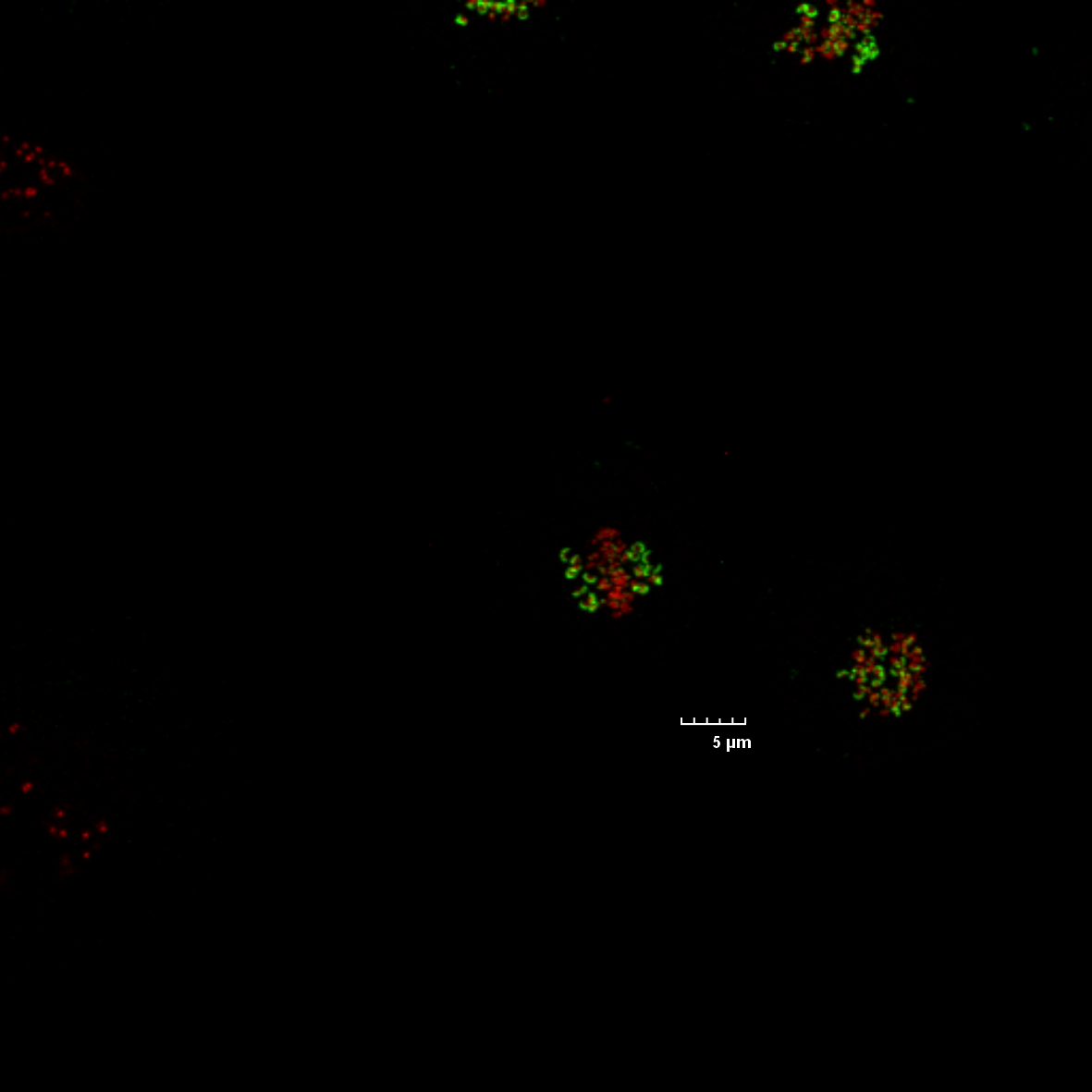

Supplement: Supplementary file 6 — Source Data Fig. 3 [file 44319_2024_106_MOESM6_ESM.zip › Figure 3/3J/CKAP5 esiRNA+partial Noco/merge.tif]

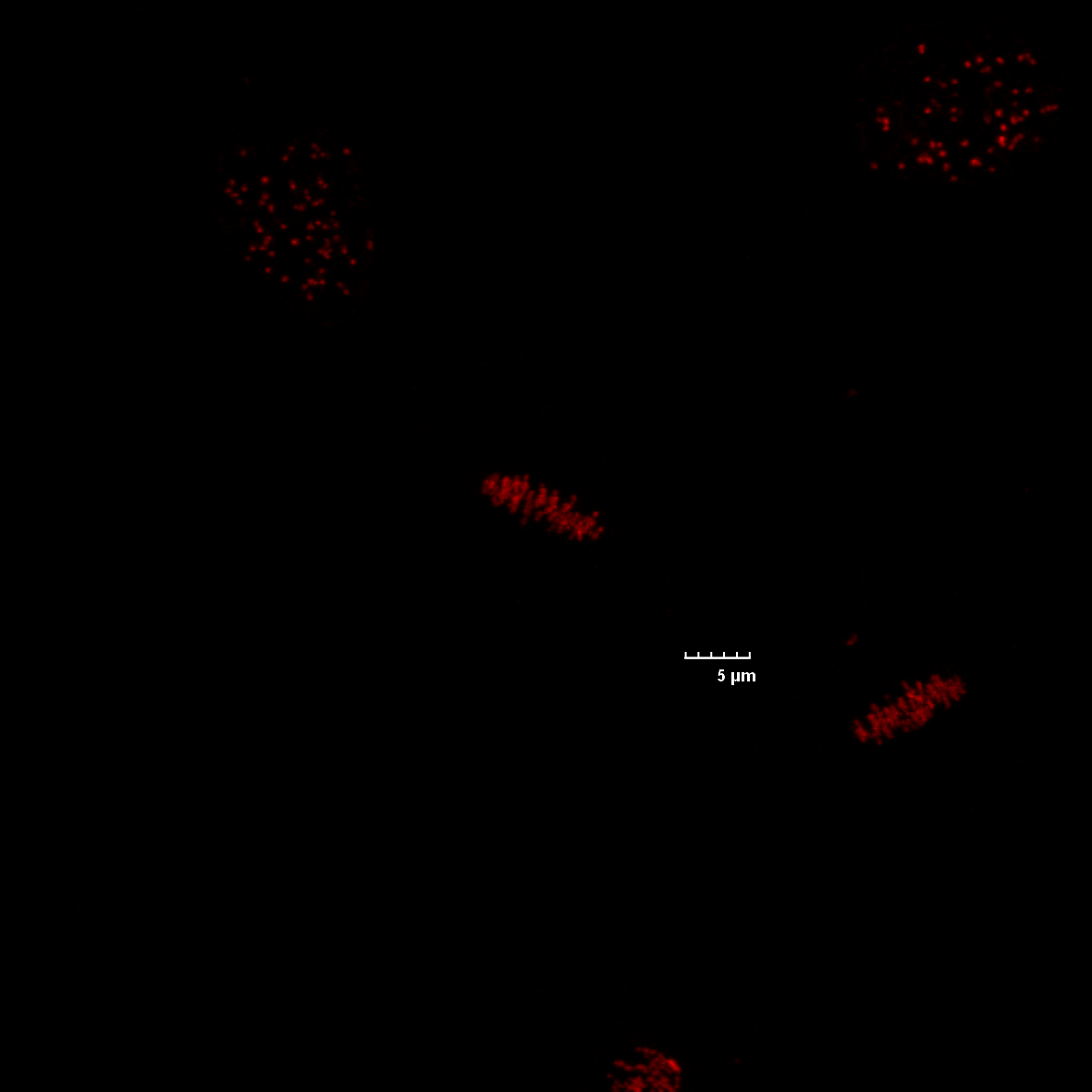

Supplement: Supplementary file 6 — Source Data Fig. 3 [file 44319_2024_106_MOESM6_ESM.zip › Figure 3/3J/Control esiRNA/aca.tif]

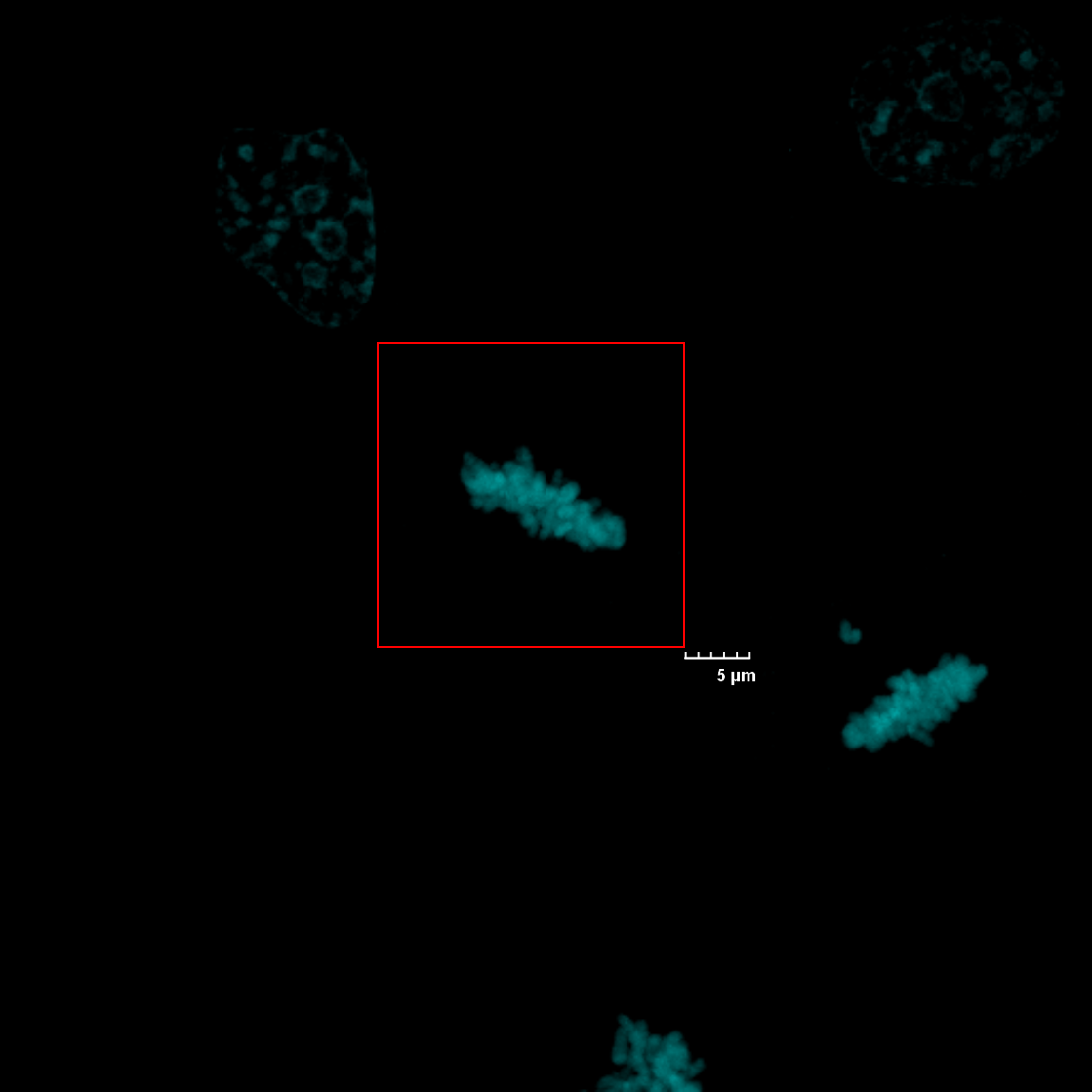

Supplement: Supplementary file 6 — Source Data Fig. 3 [file 44319_2024_106_MOESM6_ESM.zip › Figure 3/3J/Control esiRNA/Annotation.tif]

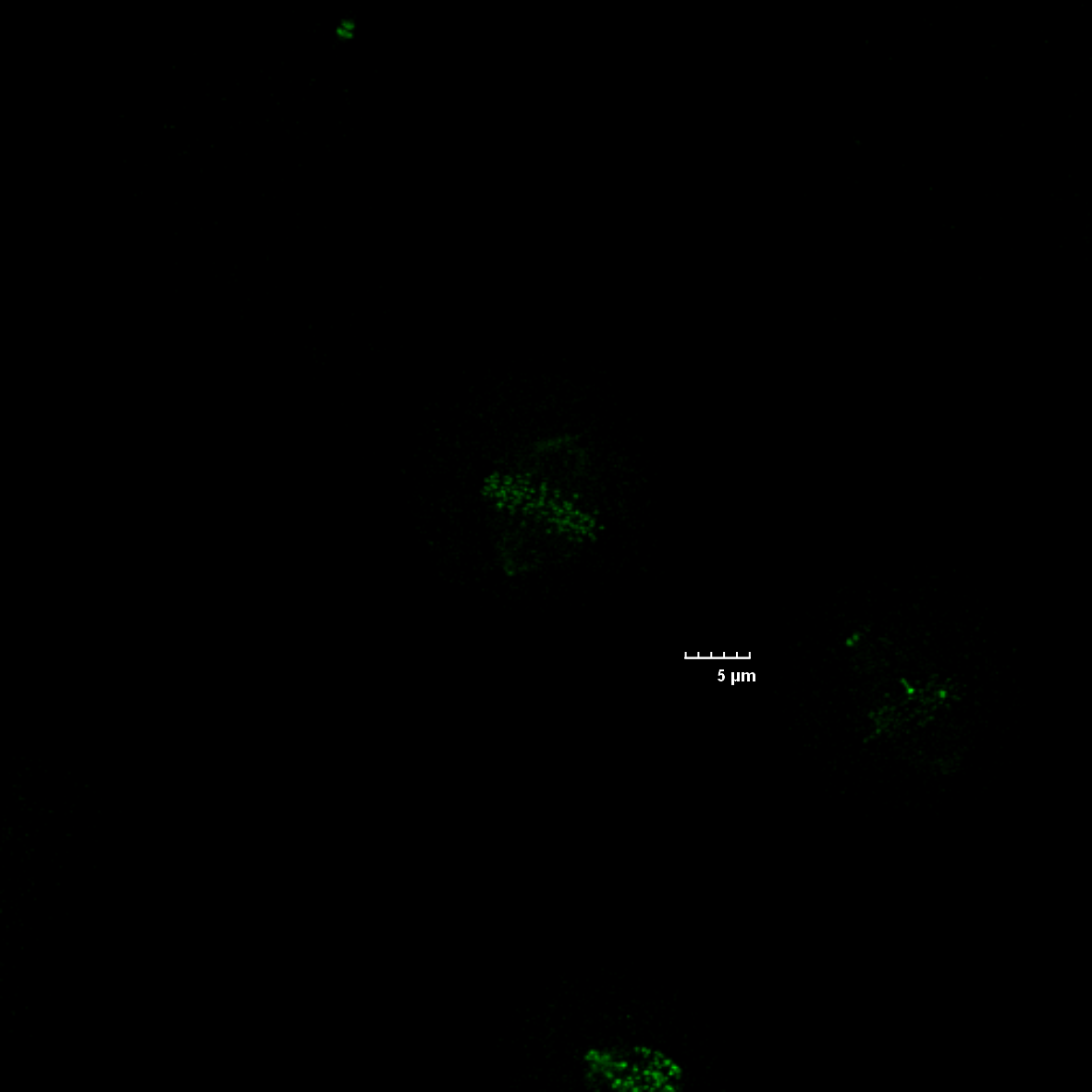

Supplement: Supplementary file 6 — Source Data Fig. 3 [file 44319_2024_106_MOESM6_ESM.zip › Figure 3/3J/Control esiRNA/cenpe.tif]
