# Supplementary figures and images for: CKAP5 stabilizes CENP-E at kinetochores by regulating microtubule-chromosome attachments (part 2 of 2)
Source: EMBO Rep. 2024 Feb 29;25(4):1909–35. doi: 10.1038/s44319-024-00106-9 (PMC11014917; doi:10.1038/s44319-024-00106-9)

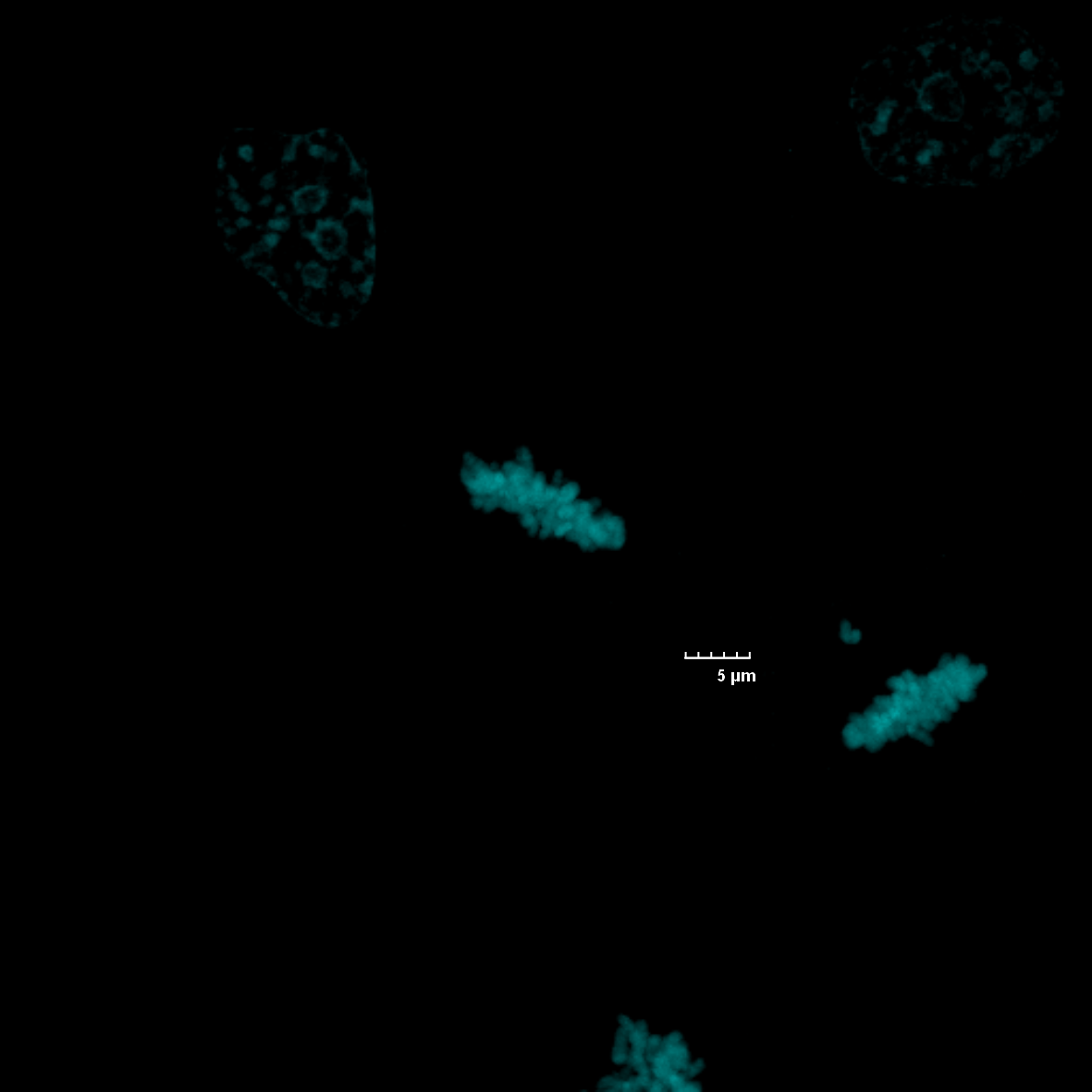

Supplement: Supplementary file 6 — Source Data Fig. 3 [file 44319_2024_106_MOESM6_ESM.zip › Figure 3/3J/Control esiRNA/dapi.tif]

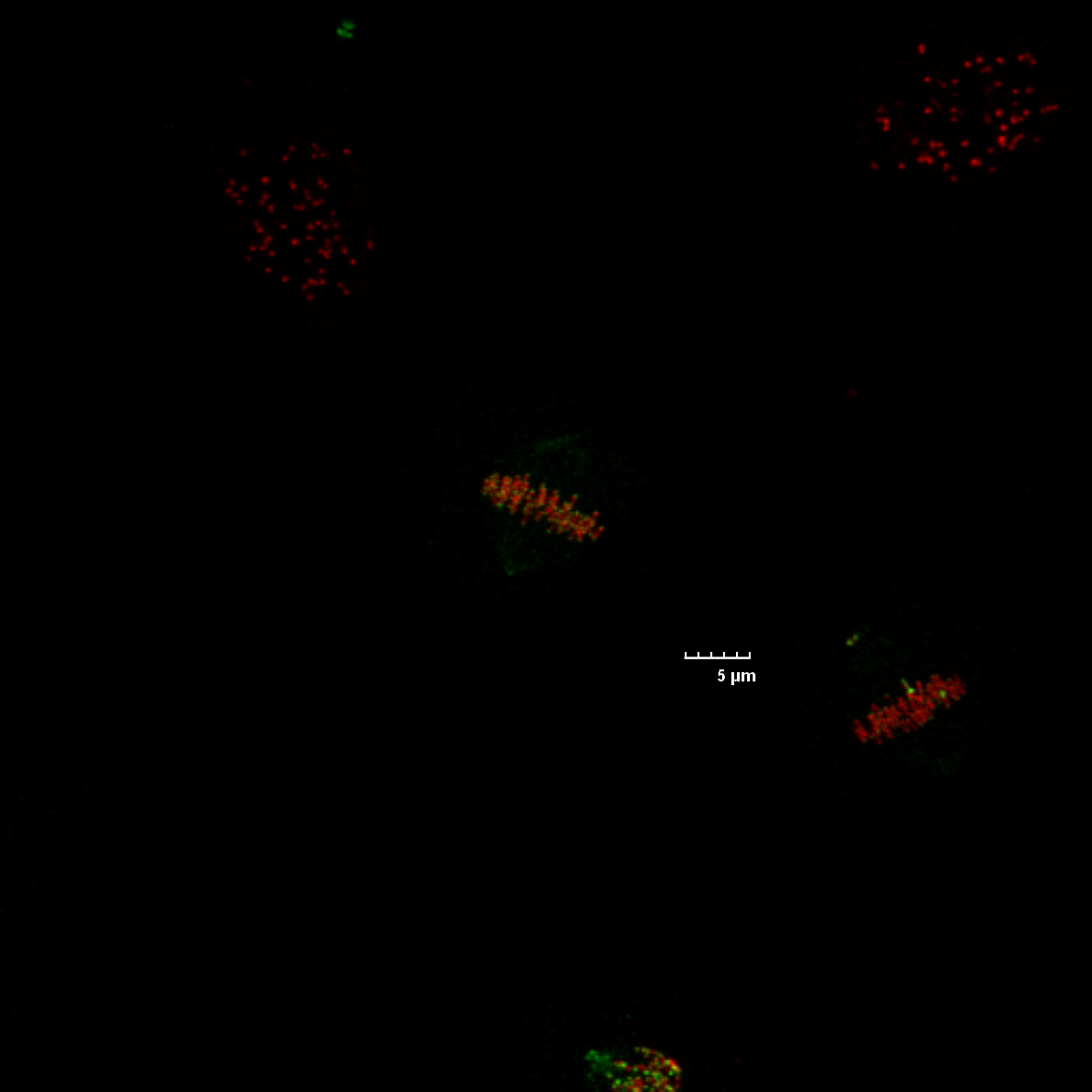

Supplement: Supplementary file 6 — Source Data Fig. 3 [file 44319_2024_106_MOESM6_ESM.zip › Figure 3/3J/Control esiRNA/merge.tif]

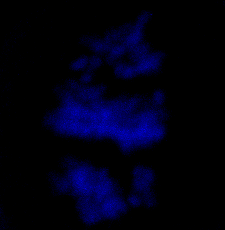

Supplement: Supplementary file 6 — Source Data Fig. 3 [file 44319_2024_106_MOESM6_ESM.zip › Figure 3/3L/KK_AA/MAX_45ctd_kk_aa_saju_n=2_cenpecy5_knl1568_160622.lif - Series027 - C=0-1.tif]

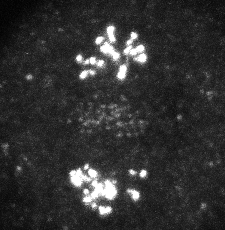

Supplement: Supplementary file 6 — Source Data Fig. 3 [file 44319_2024_106_MOESM6_ESM.zip › Figure 3/3L/KK_AA/MAX_45ctd_kk_aa_saju_n=2_cenpecy5_knl1568_160622.lif - Series027 - C=2.tif]

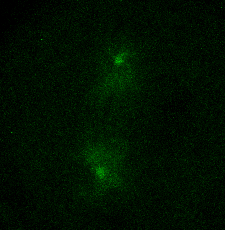

Supplement: Supplementary file 6 — Source Data Fig. 3 [file 44319_2024_106_MOESM6_ESM.zip › Figure 3/3L/KK_AA/MAX_45ctd_kk_aa_saju_n=2_cenpecy5_knl1568_160622.lif - Series027 - C=3-1.tif]

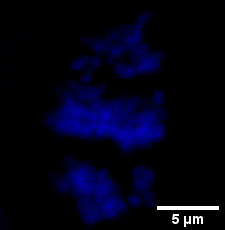

Supplement: Supplementary file 6 — Source Data Fig. 3 [file 44319_2024_106_MOESM6_ESM.zip › Figure 3/3L/KK_AA/scale.tif]

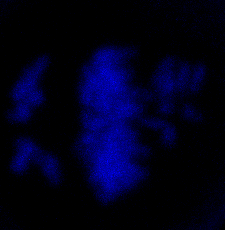

Supplement: Supplementary file 6 — Source Data Fig. 3 [file 44319_2024_106_MOESM6_ESM.zip › Figure 3/3L/WT/MAX_45ctd_saju_n=3_cenpecy5_knl1568_180622.lif - Series026 - C=0-1.tif]

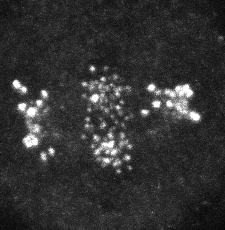

Supplement: Supplementary file 6 — Source Data Fig. 3 [file 44319_2024_106_MOESM6_ESM.zip › Figure 3/3L/WT/MAX_45ctd_saju_n=3_cenpecy5_knl1568_180622.lif - Series026 - C=2.tif]

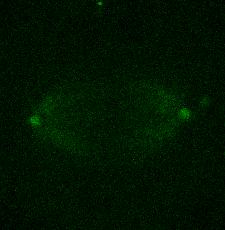

Supplement: Supplementary file 6 — Source Data Fig. 3 [file 44319_2024_106_MOESM6_ESM.zip › Figure 3/3L/WT/MAX_45ctd_saju_n=3_cenpecy5_knl1568_180622.lif - Series026 - C=3-1.tif]

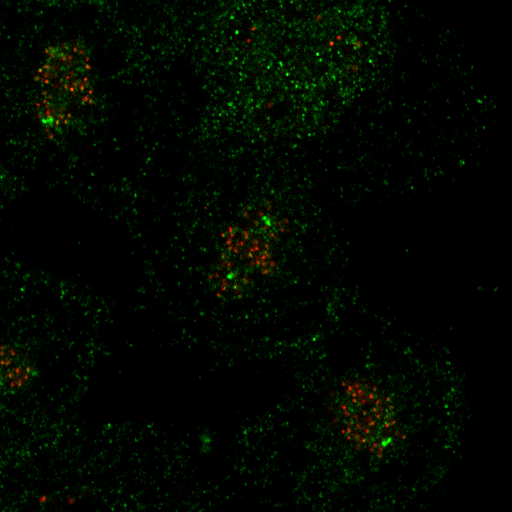

Supplement: Supplementary file 7 — Source Data Fig. 4 [file 44319_2024_106_MOESM7_ESM.zip › Figure 4/4A/CKAP5 esiRNA/14.tif]

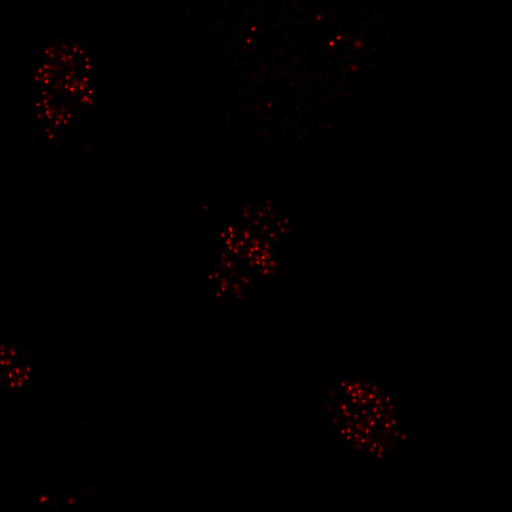

Supplement: Supplementary file 7 — Source Data Fig. 4 [file 44319_2024_106_MOESM7_ESM.zip › Figure 4/4A/CKAP5 esiRNA/14.tif1.tif]

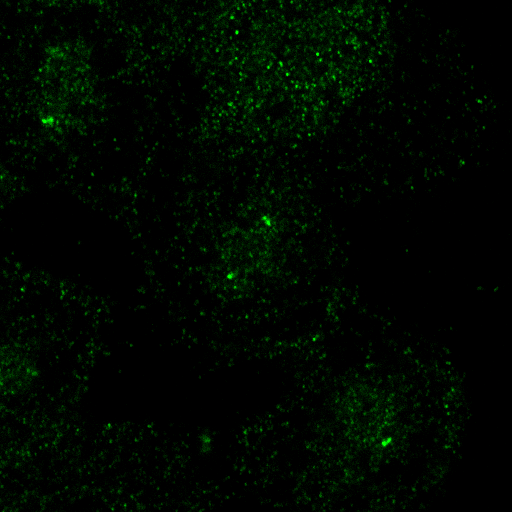

Supplement: Supplementary file 7 — Source Data Fig. 4 [file 44319_2024_106_MOESM7_ESM.zip › Figure 4/4A/CKAP5 esiRNA/14.tif2.tif]

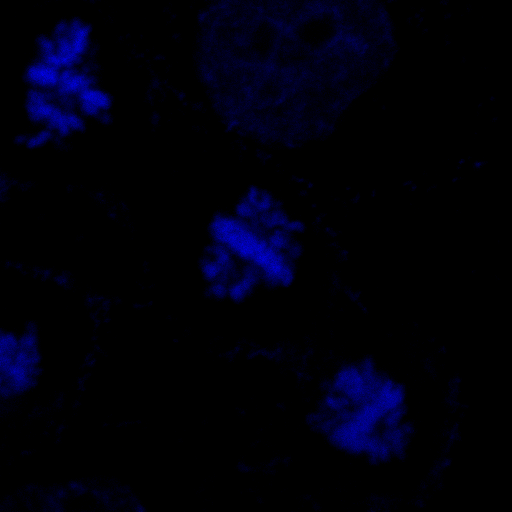

Supplement: Supplementary file 7 — Source Data Fig. 4 [file 44319_2024_106_MOESM7_ESM.zip › Figure 4/4A/CKAP5 esiRNA/14.tif4.tif]

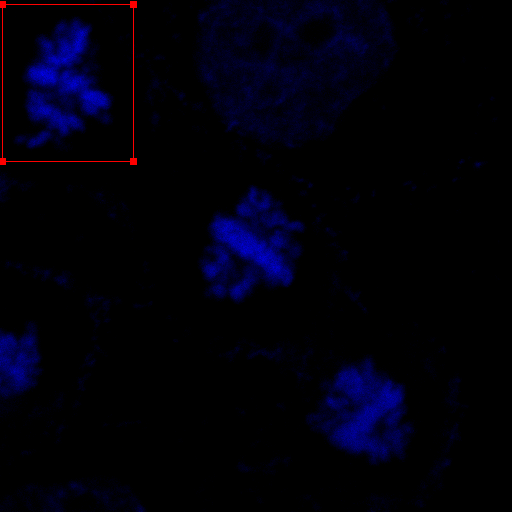

Supplement: Supplementary file 7 — Source Data Fig. 4 [file 44319_2024_106_MOESM7_ESM.zip › Figure 4/4A/CKAP5 esiRNA/Annotation_14.tif]

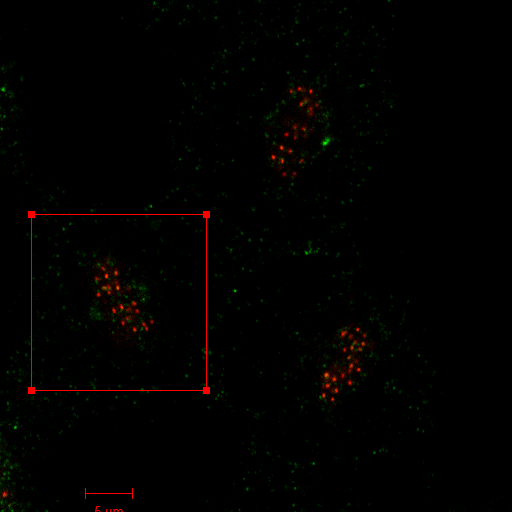

Supplement: Supplementary file 7 — Source Data Fig. 4 [file 44319_2024_106_MOESM7_ESM.zip › Figure 4/4A/Control esiRNA/Annotation_Image 4.tif]

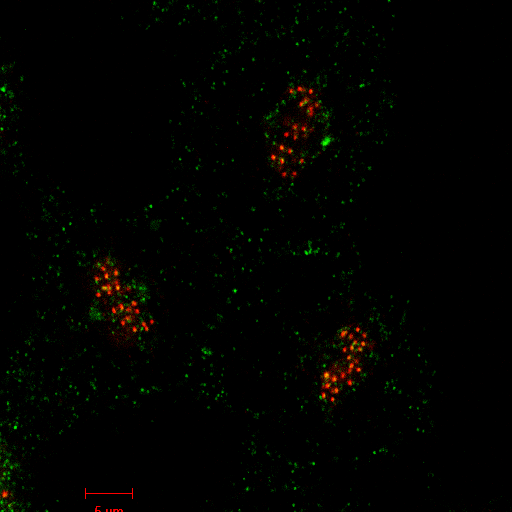

Supplement: Supplementary file 7 — Source Data Fig. 4 [file 44319_2024_106_MOESM7_ESM.zip › Figure 4/4A/Control esiRNA/Image 4.tif]

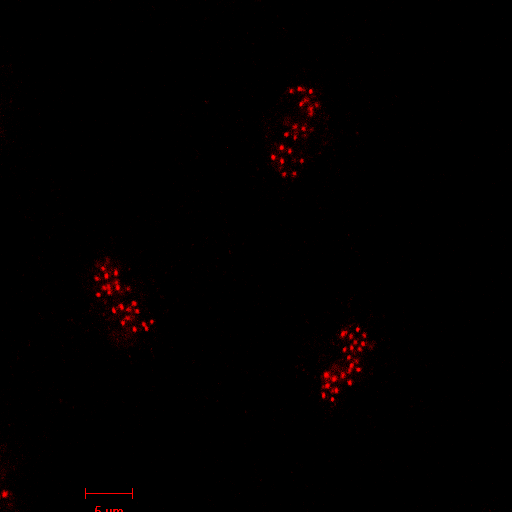

Supplement: Supplementary file 7 — Source Data Fig. 4 [file 44319_2024_106_MOESM7_ESM.zip › Figure 4/4A/Control esiRNA/Image 4.tif1.tif]

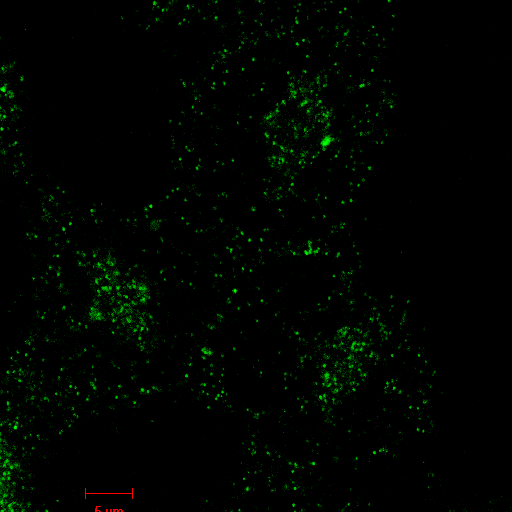

Supplement: Supplementary file 7 — Source Data Fig. 4 [file 44319_2024_106_MOESM7_ESM.zip › Figure 4/4A/Control esiRNA/Image 4.tif2.tif]

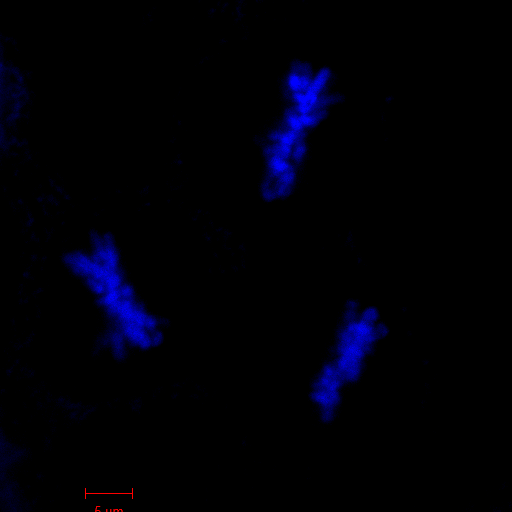

Supplement: Supplementary file 7 — Source Data Fig. 4 [file 44319_2024_106_MOESM7_ESM.zip › Figure 4/4A/Control esiRNA/Image 4.tif4.tif]

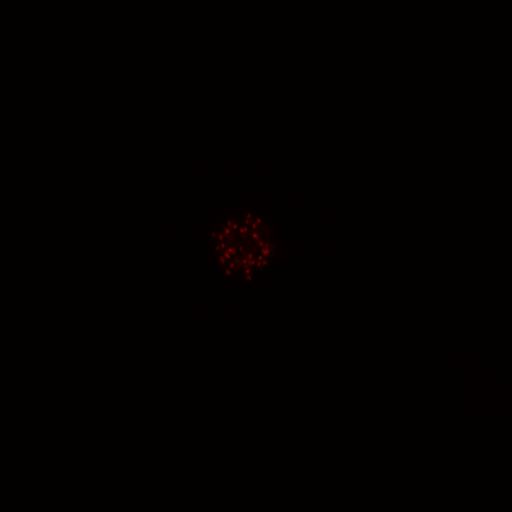

Supplement: Supplementary file 7 — Source Data Fig. 4 [file 44319_2024_106_MOESM7_ESM.zip › Figure 4/4C/CKAP5 esiRNA/Image 18.tif]

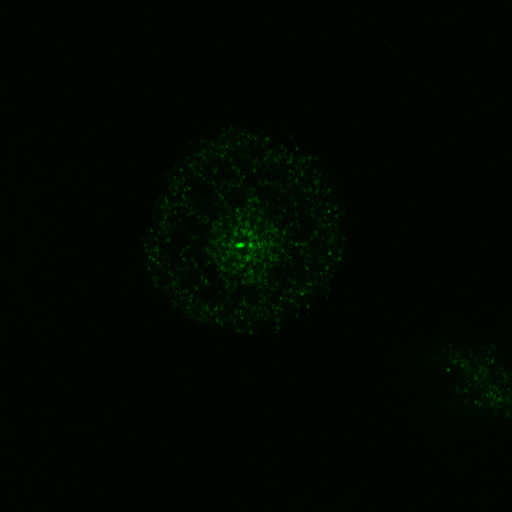

Supplement: Supplementary file 7 — Source Data Fig. 4 [file 44319_2024_106_MOESM7_ESM.zip › Figure 4/4C/CKAP5 esiRNA/Image 18.tif1.tif]

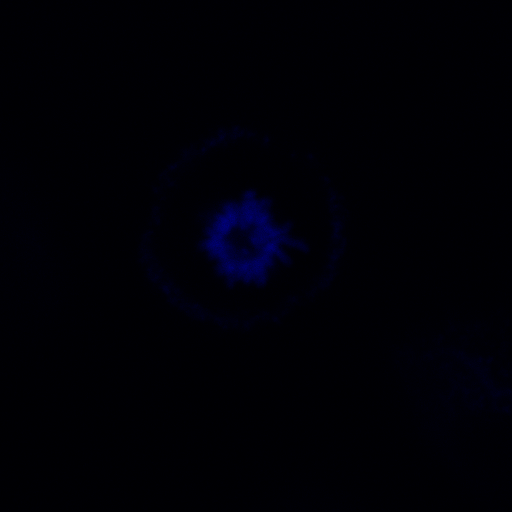

Supplement: Supplementary file 7 — Source Data Fig. 4 [file 44319_2024_106_MOESM7_ESM.zip › Figure 4/4C/CKAP5 esiRNA/Image 18.tif2.tif]

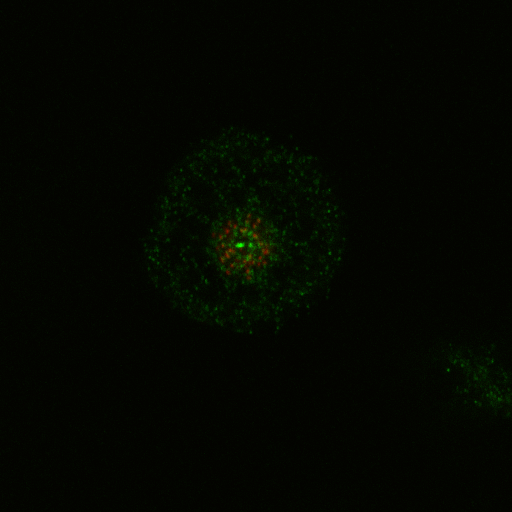

Supplement: Supplementary file 7 — Source Data Fig. 4 [file 44319_2024_106_MOESM7_ESM.zip › Figure 4/4C/CKAP5 esiRNA/Image 18.tif3.tif]

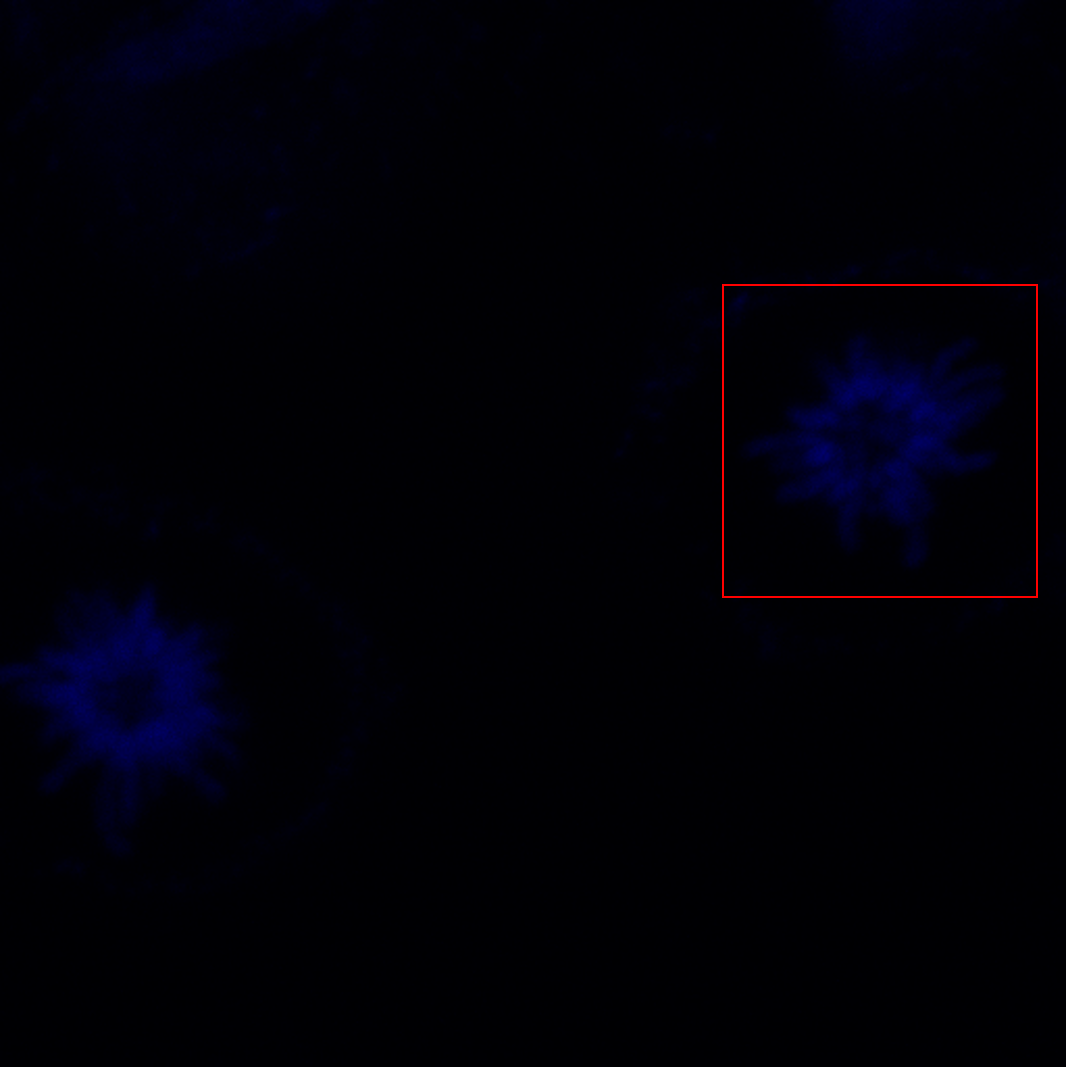

Supplement: Supplementary file 7 — Source Data Fig. 4 [file 44319_2024_106_MOESM7_ESM.zip › Figure 4/4C/Control esiRNA/Annotation.tif]

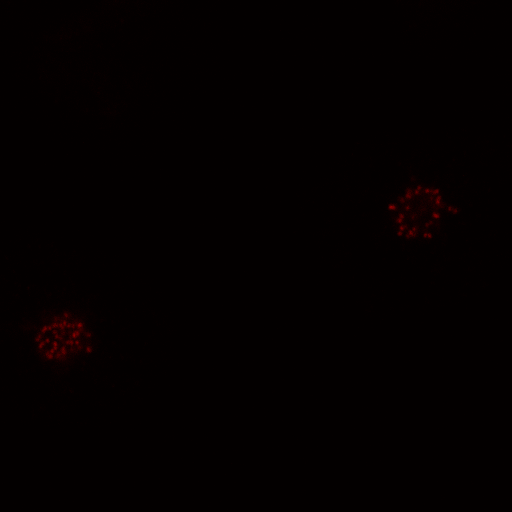

Supplement: Supplementary file 7 — Source Data Fig. 4 [file 44319_2024_106_MOESM7_ESM.zip › Figure 4/4C/Control esiRNA/Image 12.tif]

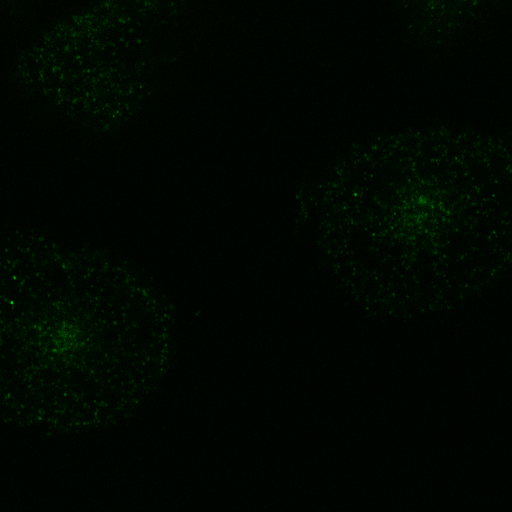

Supplement: Supplementary file 7 — Source Data Fig. 4 [file 44319_2024_106_MOESM7_ESM.zip › Figure 4/4C/Control esiRNA/Image 12.tif1.tif]

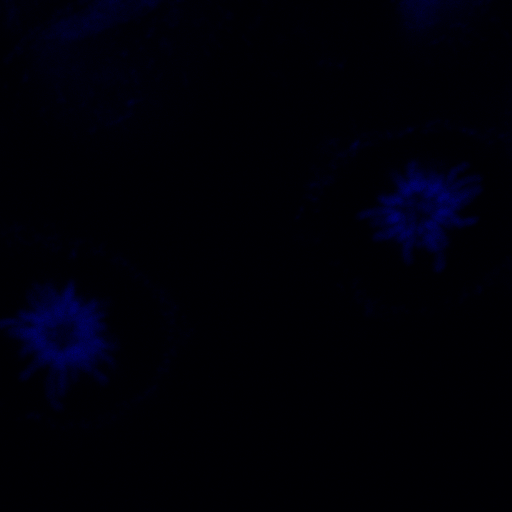

Supplement: Supplementary file 7 — Source Data Fig. 4 [file 44319_2024_106_MOESM7_ESM.zip › Figure 4/4C/Control esiRNA/Image 12.tif2.tif]

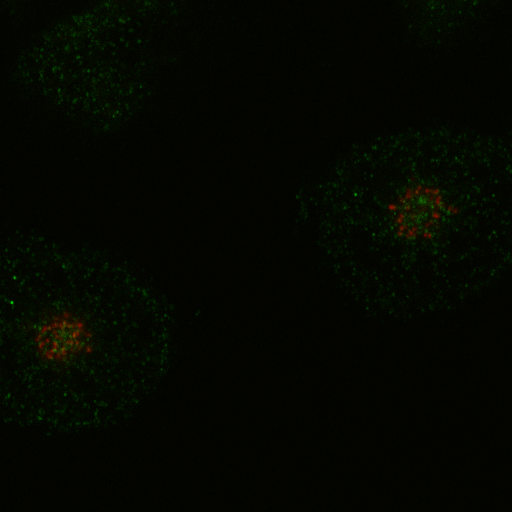

Supplement: Supplementary file 7 — Source Data Fig. 4 [file 44319_2024_106_MOESM7_ESM.zip › Figure 4/4C/Control esiRNA/Image 12.tif3.tif]

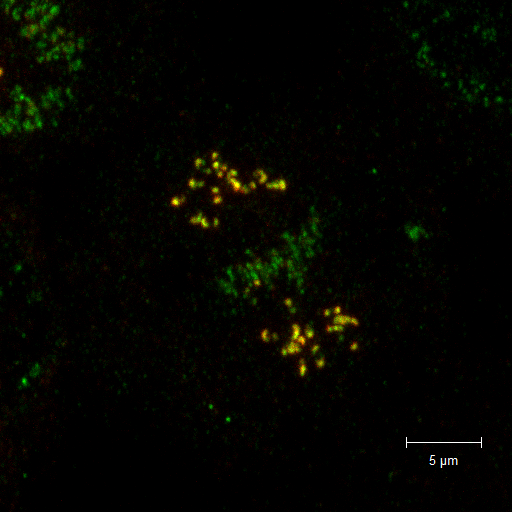

Supplement: Supplementary file 7 — Source Data Fig. 4 [file 44319_2024_106_MOESM7_ESM.zip › Figure 4/4E/CKAP5 esiRNA+ DMSO/Image 38_Maximum intensity projection.tif]

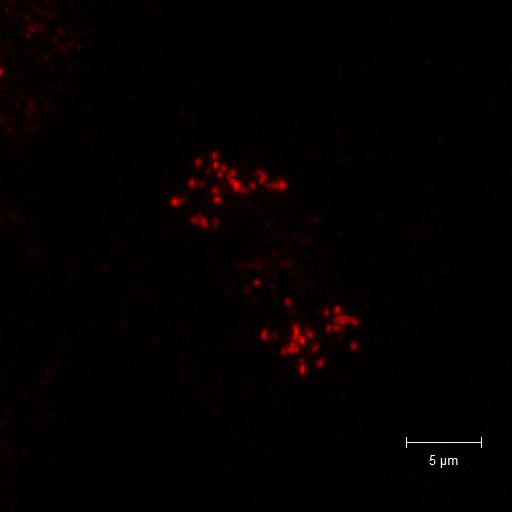

Supplement: Supplementary file 7 — Source Data Fig. 4 [file 44319_2024_106_MOESM7_ESM.zip › Figure 4/4E/CKAP5 esiRNA+ DMSO/Image 38_Maximum intensity projection.tif1.tif]

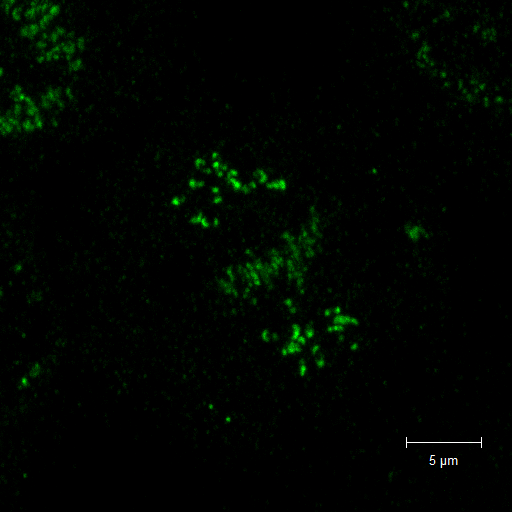

Supplement: Supplementary file 7 — Source Data Fig. 4 [file 44319_2024_106_MOESM7_ESM.zip › Figure 4/4E/CKAP5 esiRNA+ DMSO/Image 38_Maximum intensity projection.tif2.tif]

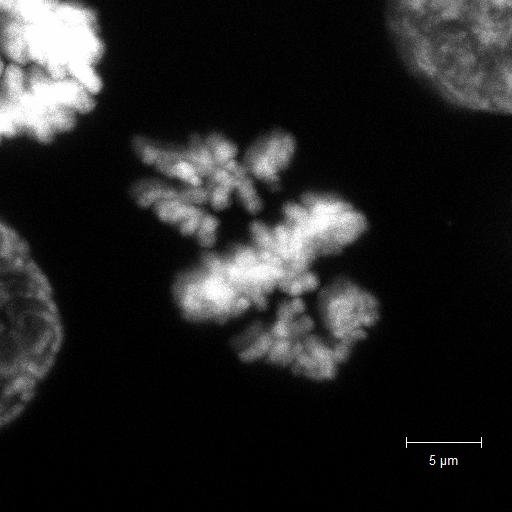

Supplement: Supplementary file 7 — Source Data Fig. 4 [file 44319_2024_106_MOESM7_ESM.zip › Figure 4/4E/CKAP5 esiRNA+ DMSO/Image 38_Maximum intensity projection.tif3.tif]

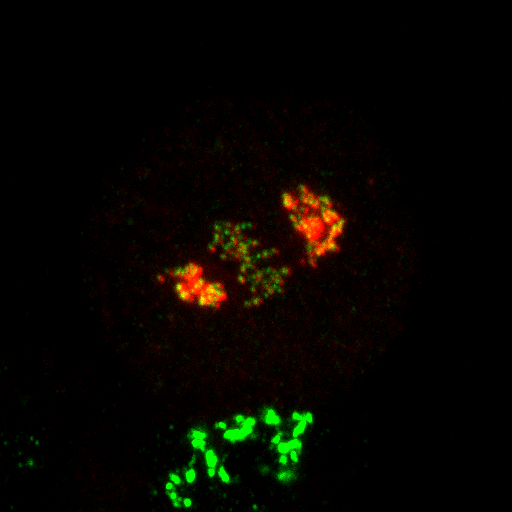

Supplement: Supplementary file 7 — Source Data Fig. 4 [file 44319_2024_106_MOESM7_ESM.zip › Figure 4/4E/CKAP5 esiRNA+ OA/Image 25_Maximum intensity projection.tif]

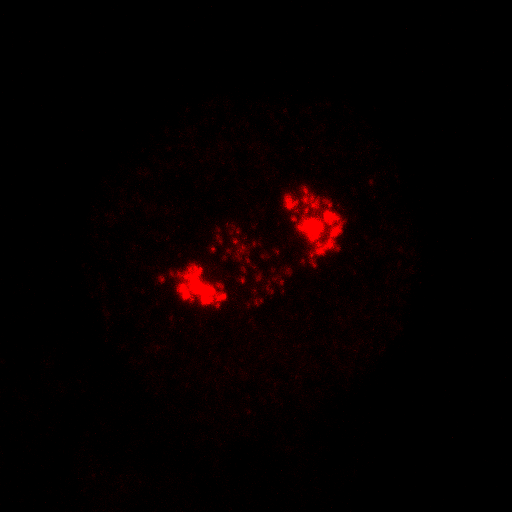

Supplement: Supplementary file 7 — Source Data Fig. 4 [file 44319_2024_106_MOESM7_ESM.zip › Figure 4/4E/CKAP5 esiRNA+ OA/Image 25_Maximum intensity projection.tif1.tif]

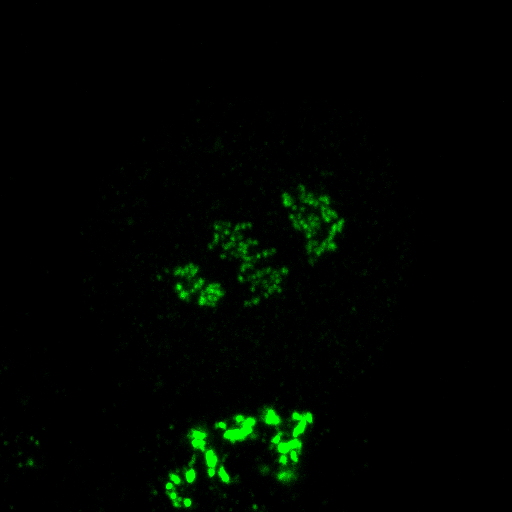

Supplement: Supplementary file 7 — Source Data Fig. 4 [file 44319_2024_106_MOESM7_ESM.zip › Figure 4/4E/CKAP5 esiRNA+ OA/Image 25_Maximum intensity projection.tif2.tif]

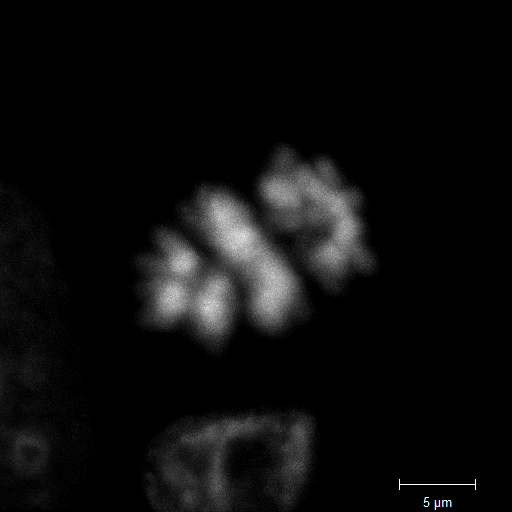

Supplement: Supplementary file 7 — Source Data Fig. 4 [file 44319_2024_106_MOESM7_ESM.zip › Figure 4/4E/CKAP5 esiRNA+ OA/Image 25_Maximum intensity projection.tif3.tif]

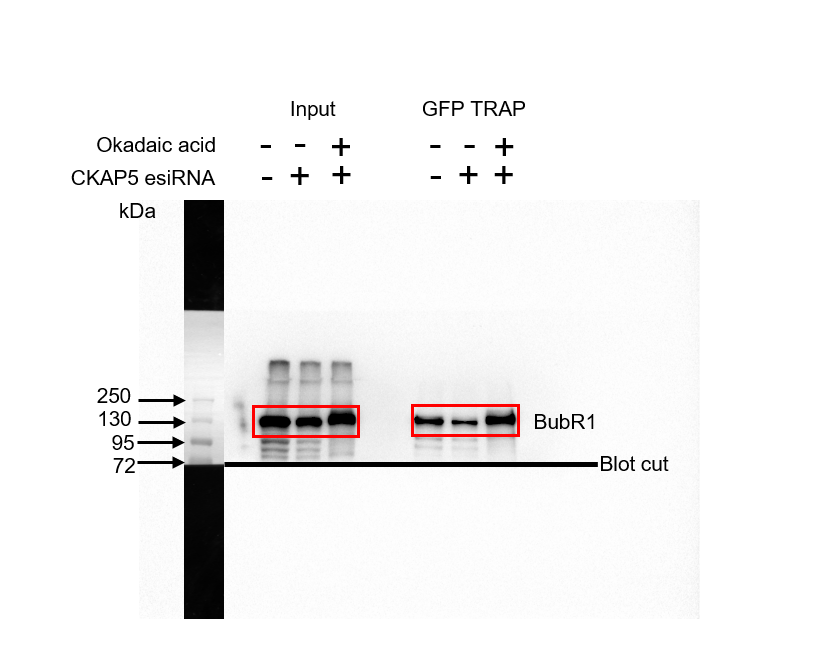

Supplement: Supplementary file 7 — Source Data Fig. 4 [file 44319_2024_106_MOESM7_ESM.zip › Figure 4/4G/BubR1/Annotation.tif]

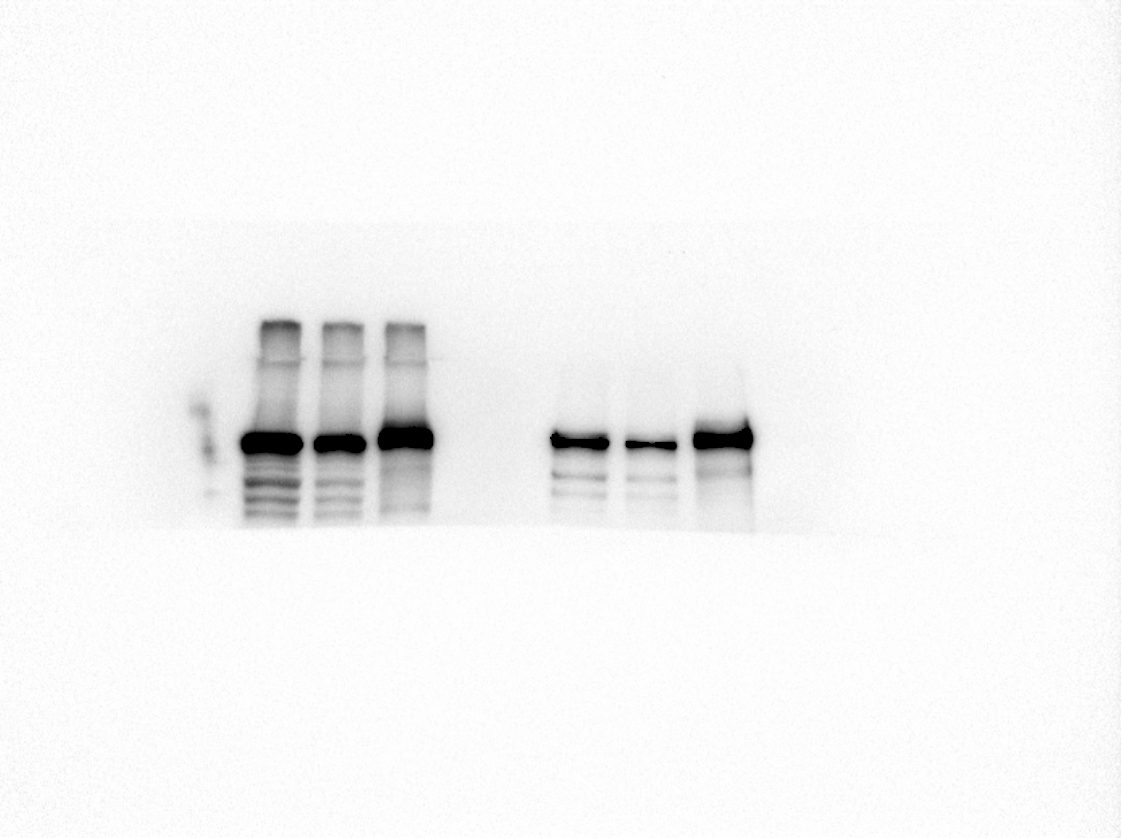

Supplement: Supplementary file 7 — Source Data Fig. 4 [file 44319_2024_106_MOESM7_ESM.zip › Figure 4/4G/BubR1/BIO-RAD 2023-01-04_15h59m59s_Exposure_30.0sec.tif]

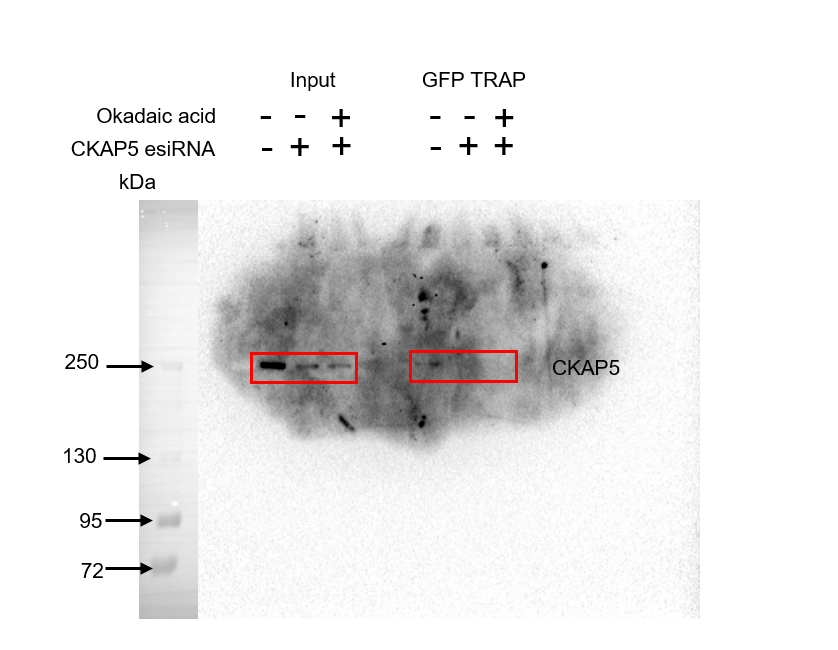

Supplement: Supplementary file 7 — Source Data Fig. 4 [file 44319_2024_106_MOESM7_ESM.zip › Figure 4/4G/CKAP5/Annotation.tif]

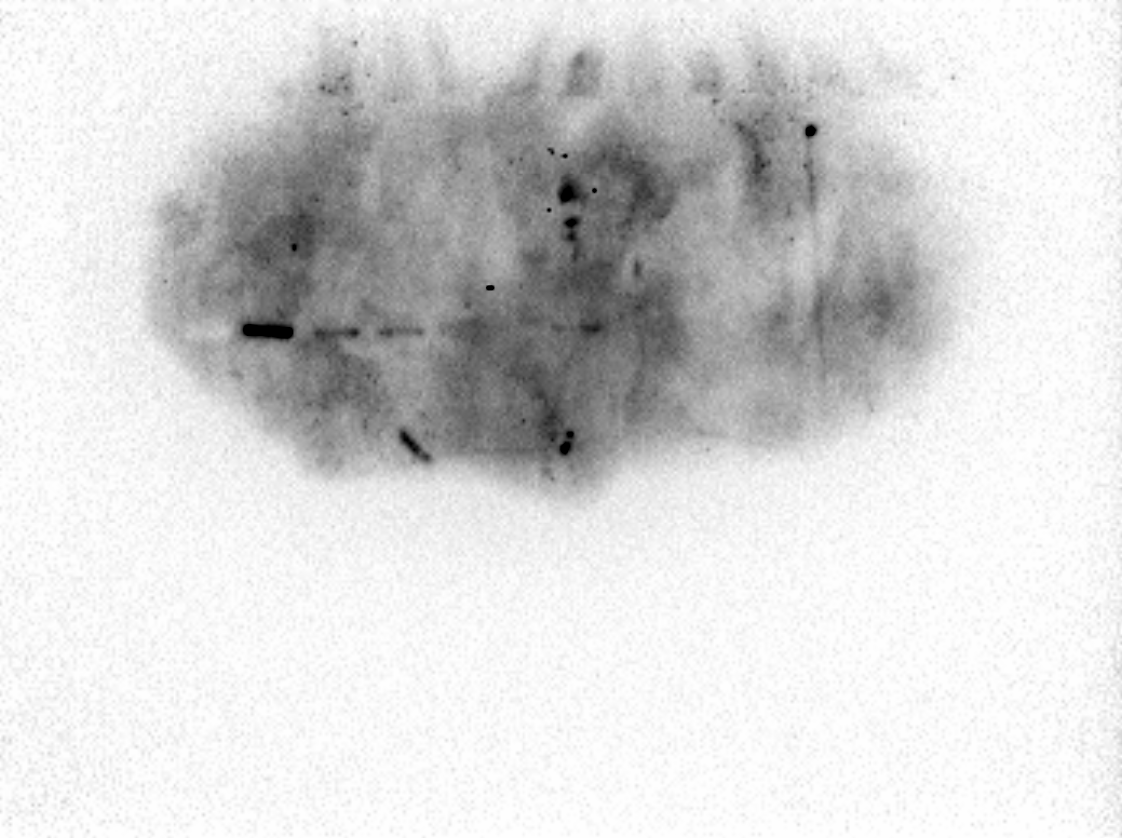

Supplement: Supplementary file 7 — Source Data Fig. 4 [file 44319_2024_106_MOESM7_ESM.zip › Figure 4/4G/CKAP5/BIO-RAD 2023-01-04_15h50m52s_Exposure_107.8sec.tif]

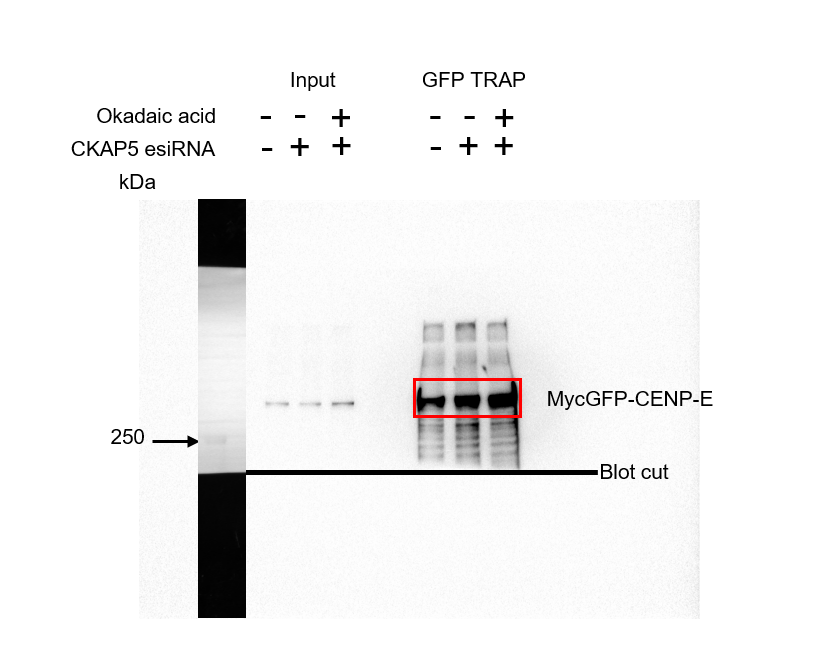

Supplement: Supplementary file 7 — Source Data Fig. 4 [file 44319_2024_106_MOESM7_ESM.zip › Figure 4/4G/MycGFP-CENP-E/GFP TRAP/Annotation.tif]

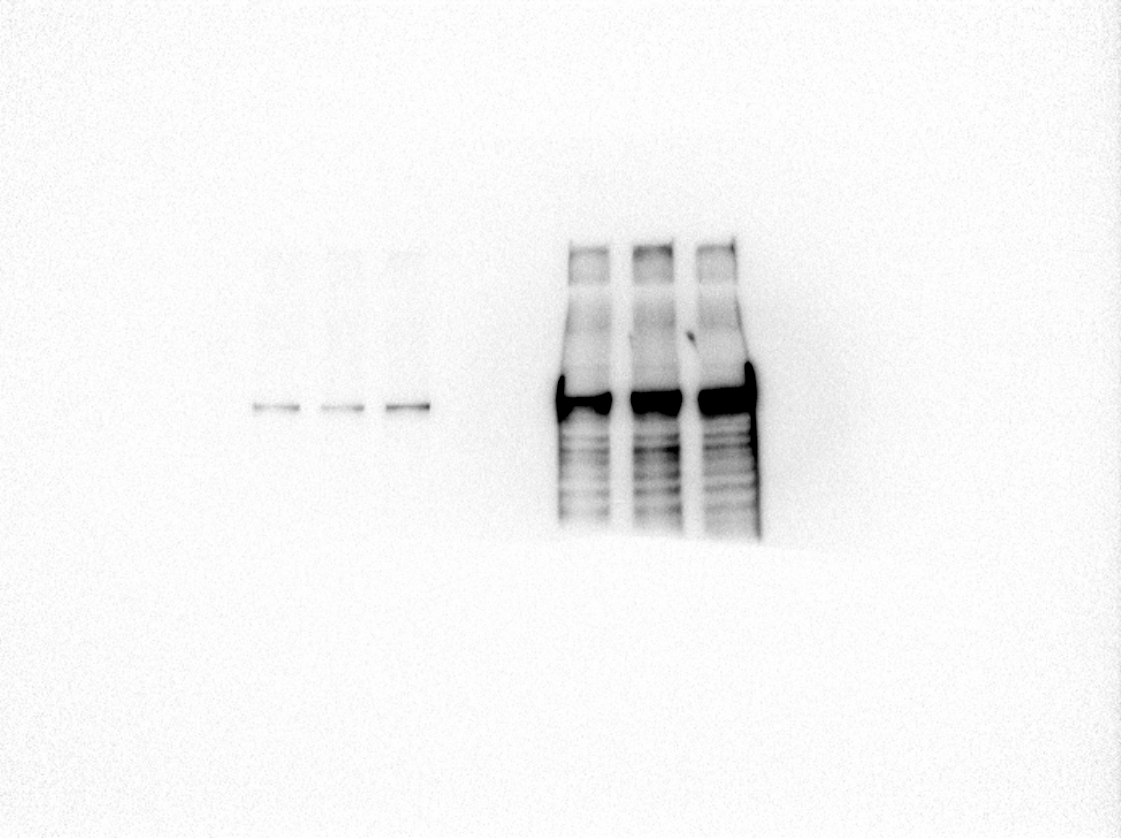

Supplement: Supplementary file 7 — Source Data Fig. 4 [file 44319_2024_106_MOESM7_ESM.zip › Figure 4/4G/MycGFP-CENP-E/GFP TRAP/TRAP.tif]

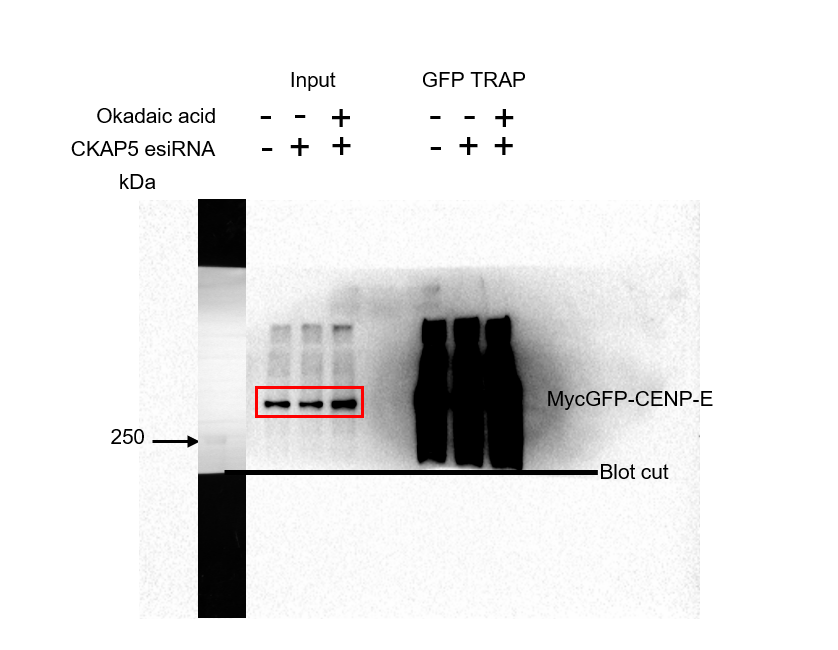

Supplement: Supplementary file 7 — Source Data Fig. 4 [file 44319_2024_106_MOESM7_ESM.zip › Figure 4/4G/MycGFP-CENP-E/Input/Annotation.tif]

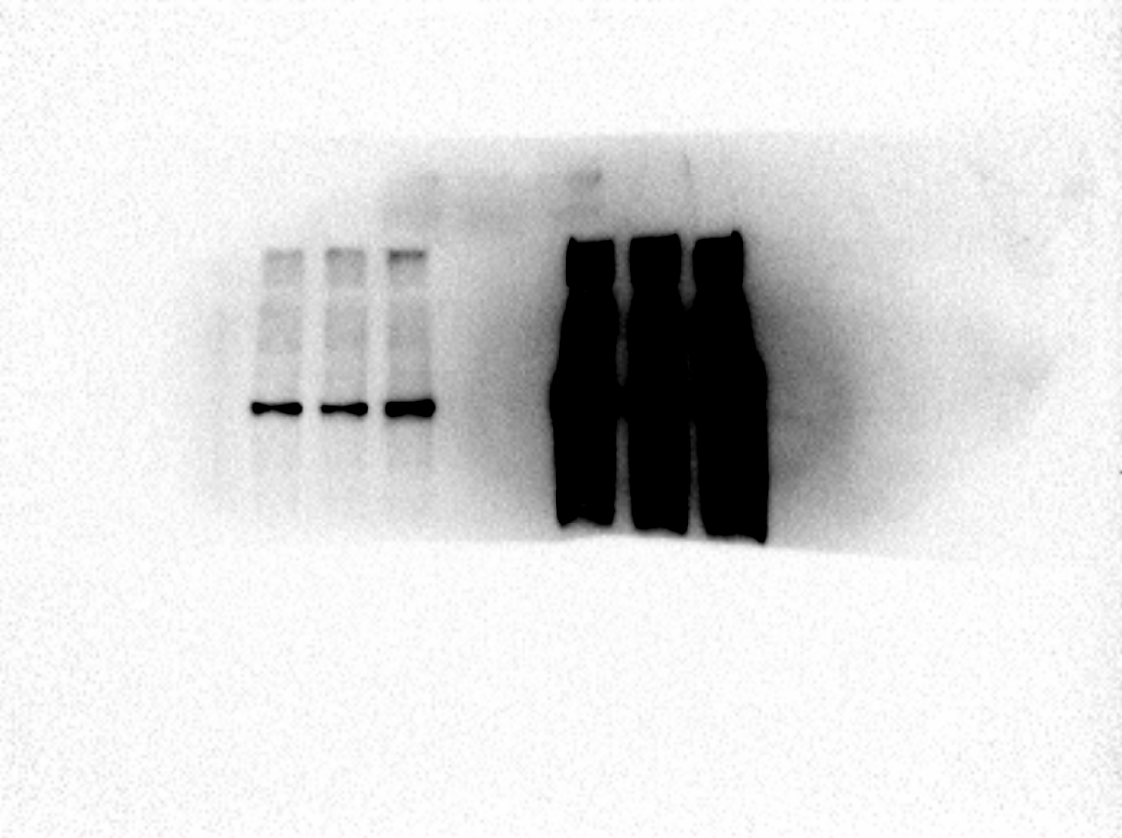

Supplement: Supplementary file 7 — Source Data Fig. 4 [file 44319_2024_106_MOESM7_ESM.zip › Figure 4/4G/MycGFP-CENP-E/Input/INPUT.tif]

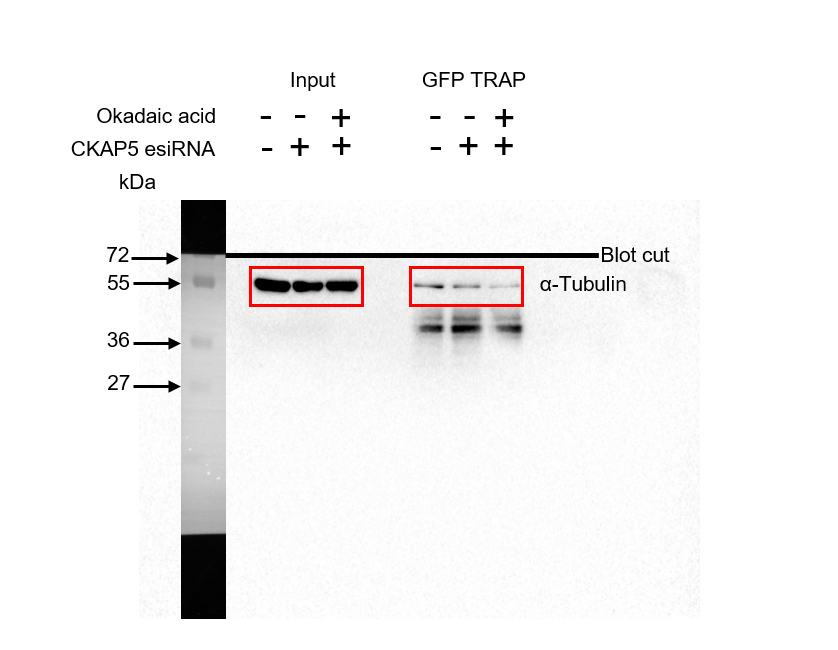

Supplement: Supplementary file 7 — Source Data Fig. 4 [file 44319_2024_106_MOESM7_ESM.zip › Figure 4/4G/Tubulin/Annotation.tif]

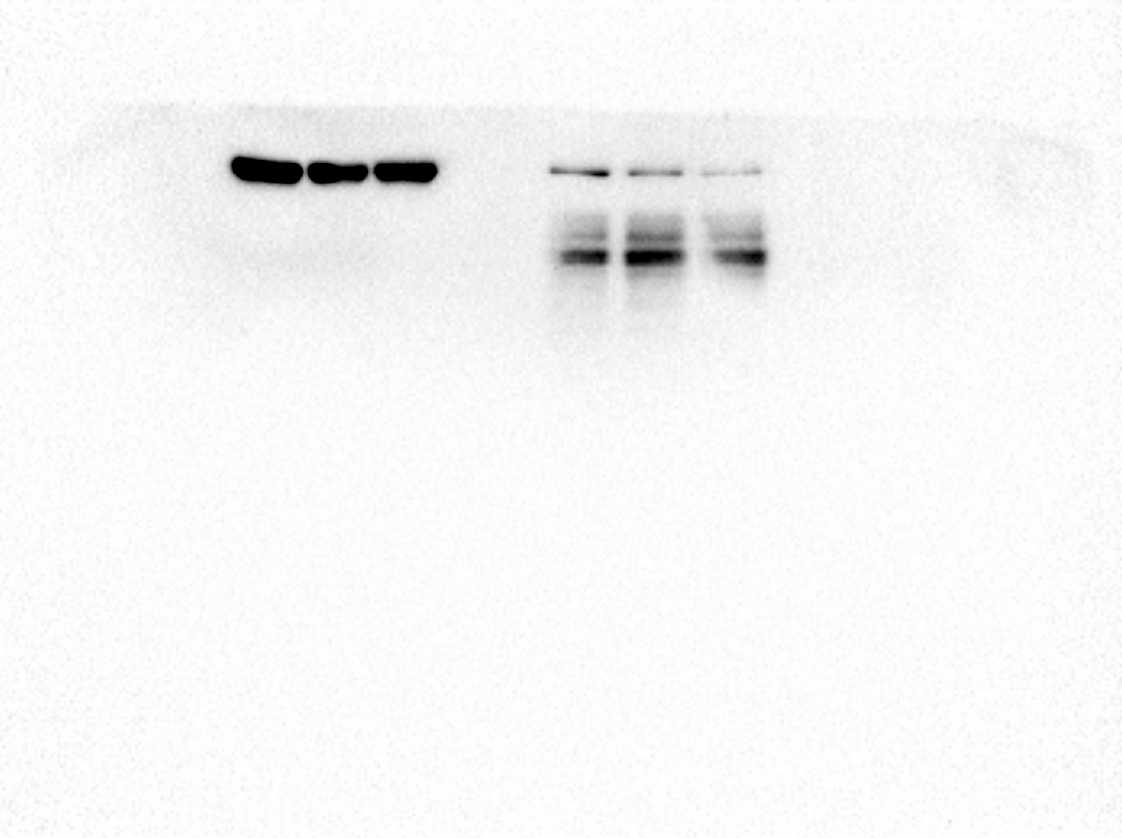

Supplement: Supplementary file 7 — Source Data Fig. 4 [file 44319_2024_106_MOESM7_ESM.zip › Figure 4/4G/Tubulin/BIO-RAD 2023-01-04_15h56m35s_Exposure_10.0sec.tif]

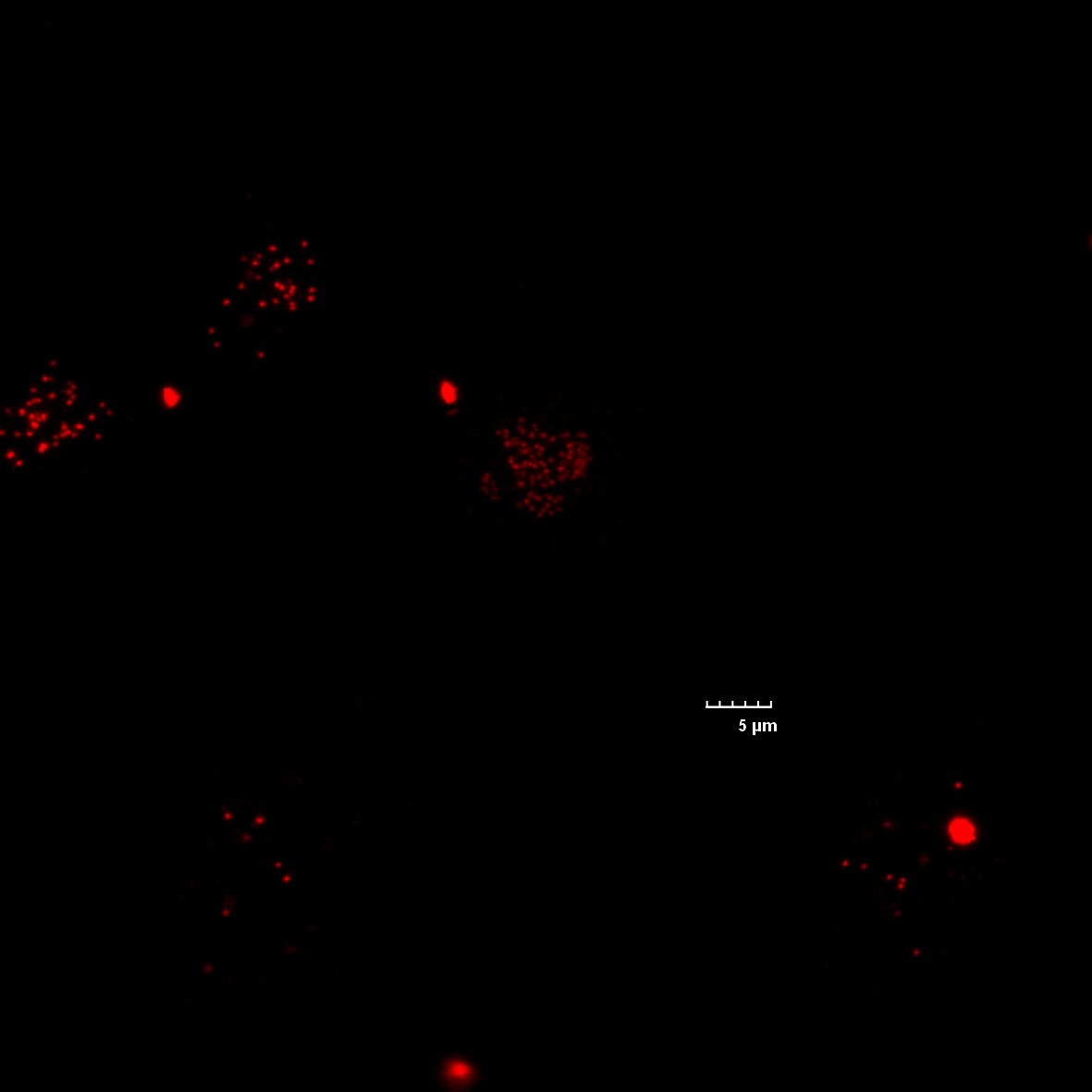

Supplement: Supplementary file 7 — Source Data Fig. 4 [file 44319_2024_106_MOESM7_ESM.zip › Figure 4/4I/+Doxycycline/CENPA.tif]

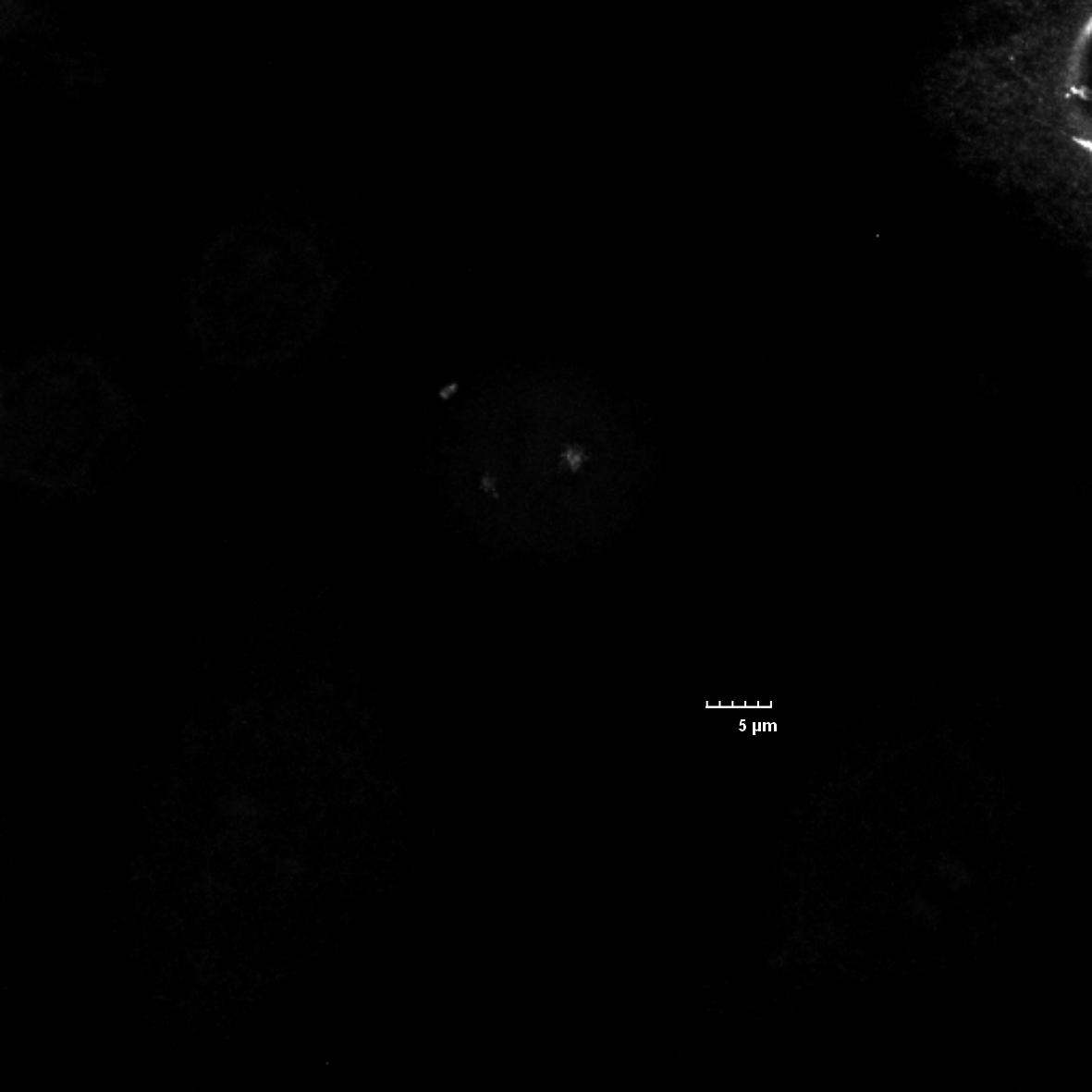

Supplement: Supplementary file 7 — Source Data Fig. 4 [file 44319_2024_106_MOESM7_ESM.zip › Figure 4/4I/+Doxycycline/GFP_GREY.tif]

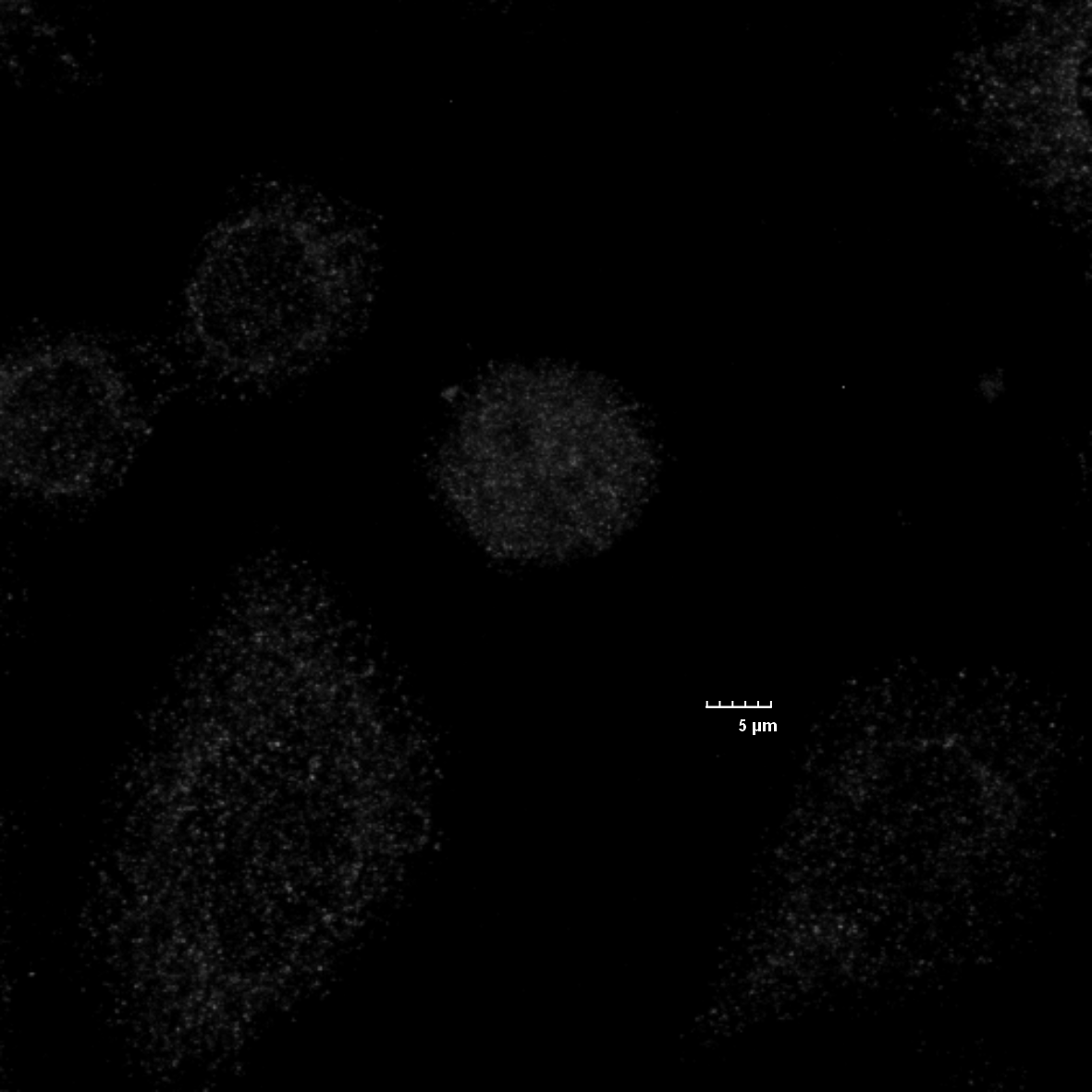

Supplement: Supplementary file 7 — Source Data Fig. 4 [file 44319_2024_106_MOESM7_ESM.zip › Figure 4/4I/+Doxycycline/TOG.tif]

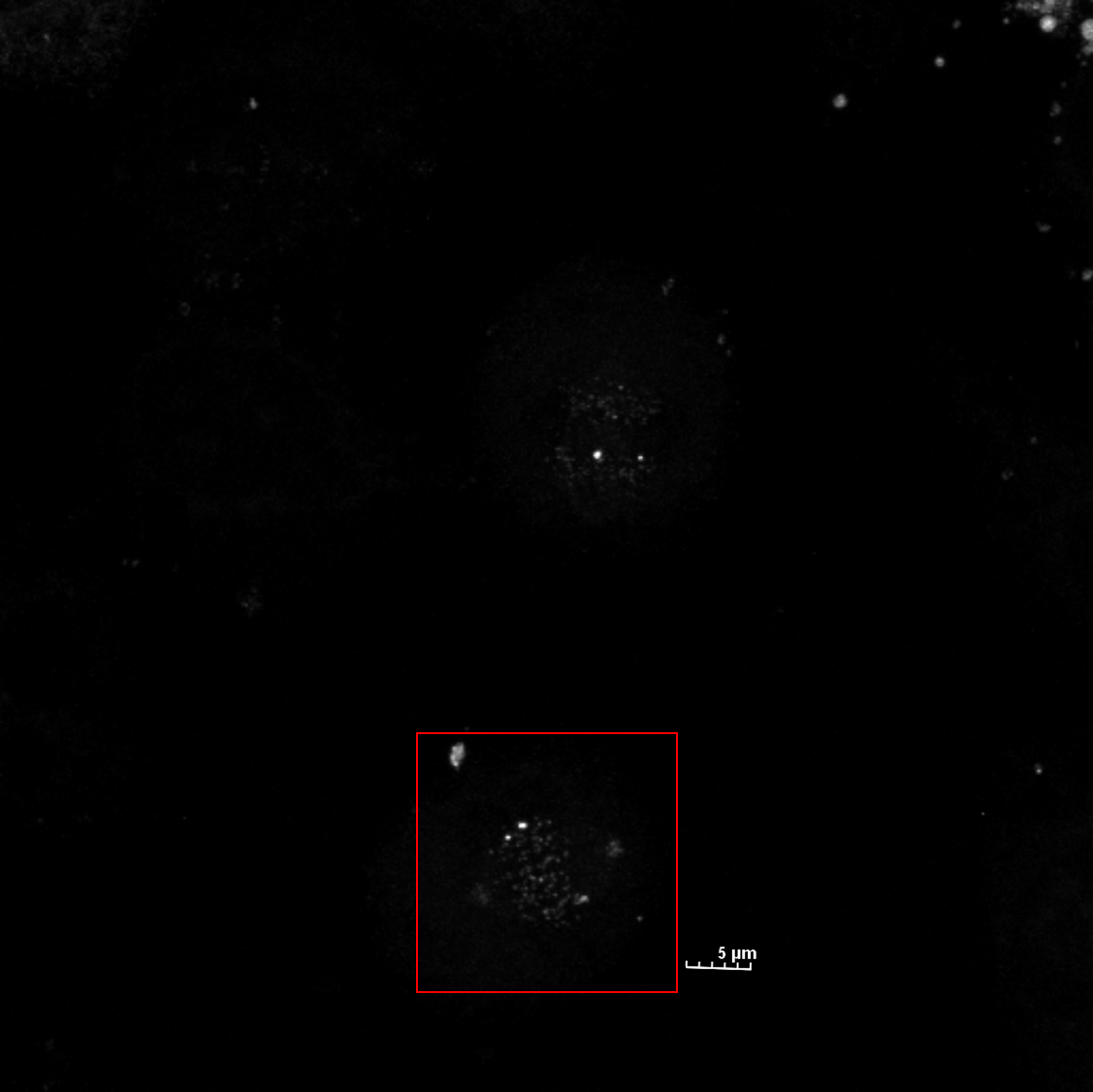

Supplement: Supplementary file 7 — Source Data Fig. 4 [file 44319_2024_106_MOESM7_ESM.zip › Figure 4/4I/- Doxycycline/Annotation.tif]

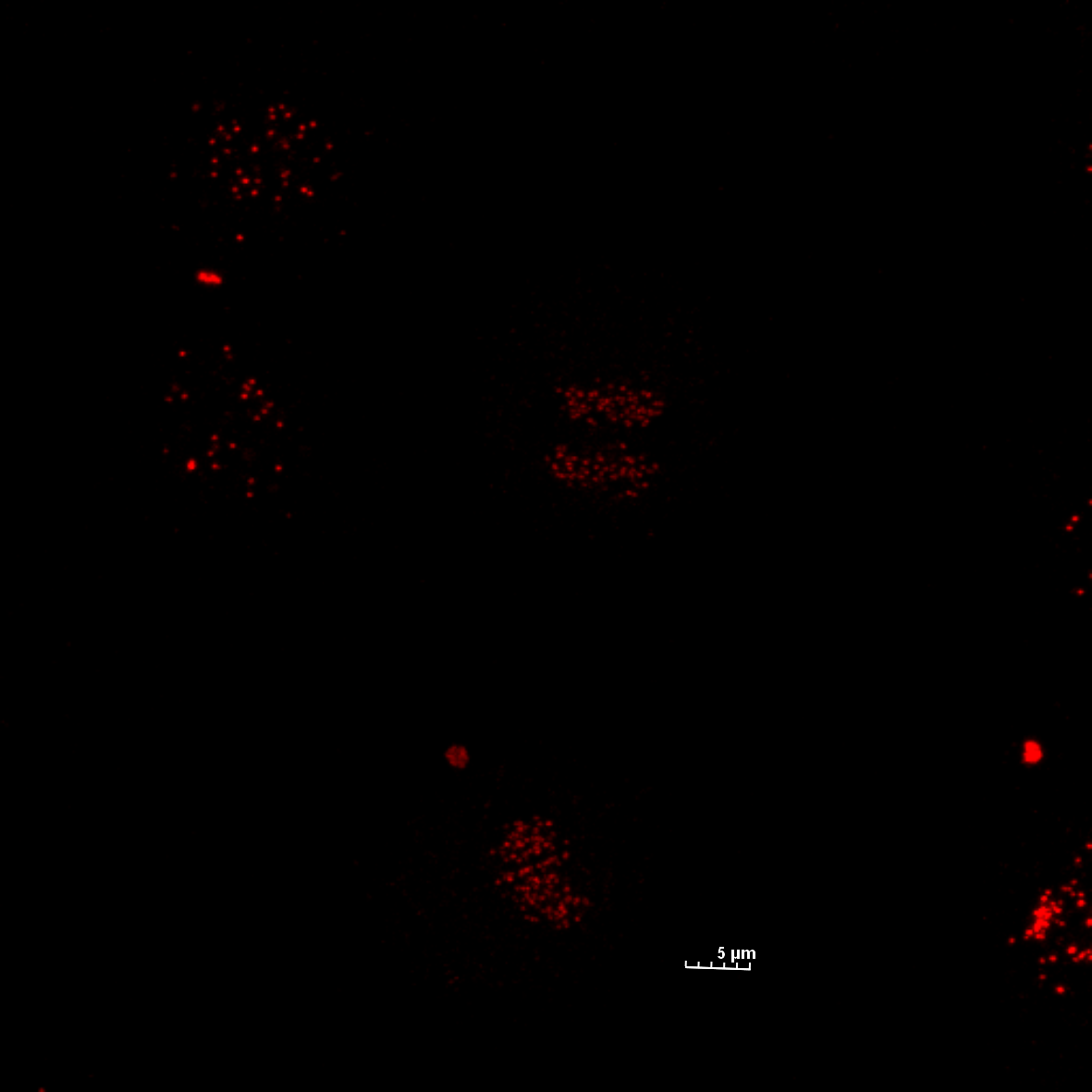

Supplement: Supplementary file 7 — Source Data Fig. 4 [file 44319_2024_106_MOESM7_ESM.zip › Figure 4/4I/- Doxycycline/CENPA.tif]

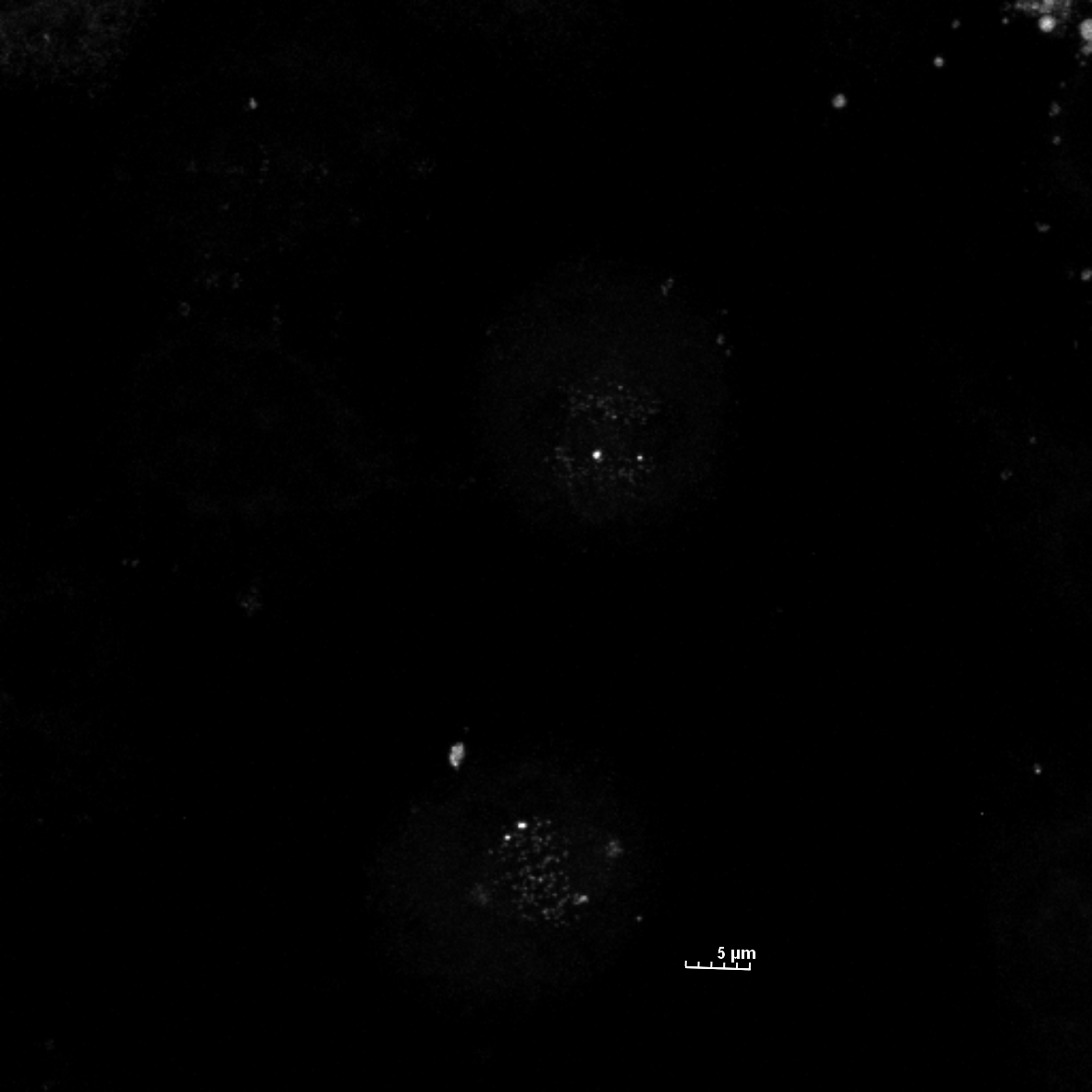

Supplement: Supplementary file 7 — Source Data Fig. 4 [file 44319_2024_106_MOESM7_ESM.zip › Figure 4/4I/- Doxycycline/GFP_GREY.tif]

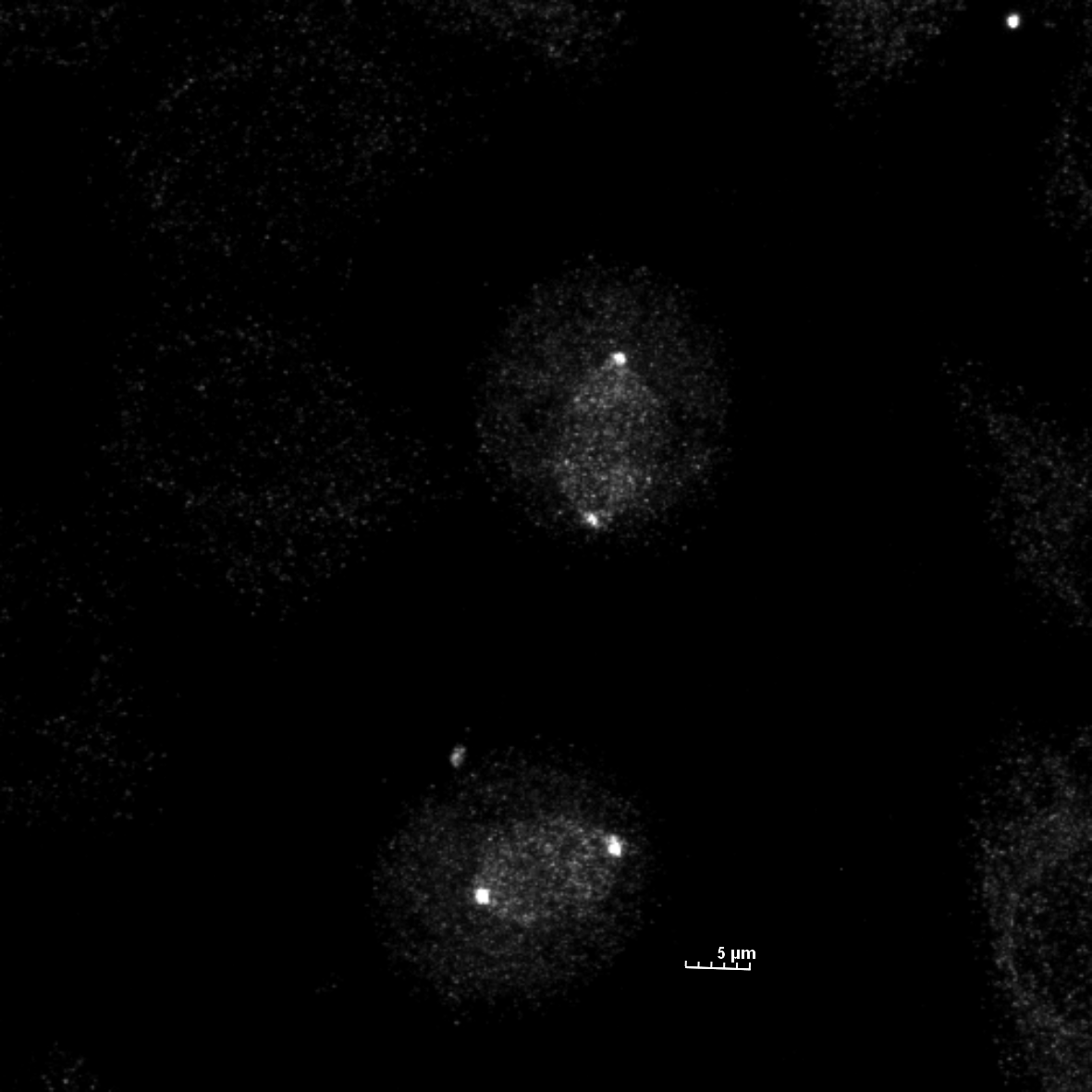

Supplement: Supplementary file 7 — Source Data Fig. 4 [file 44319_2024_106_MOESM7_ESM.zip › Figure 4/4I/- Doxycycline/TOG.tif]

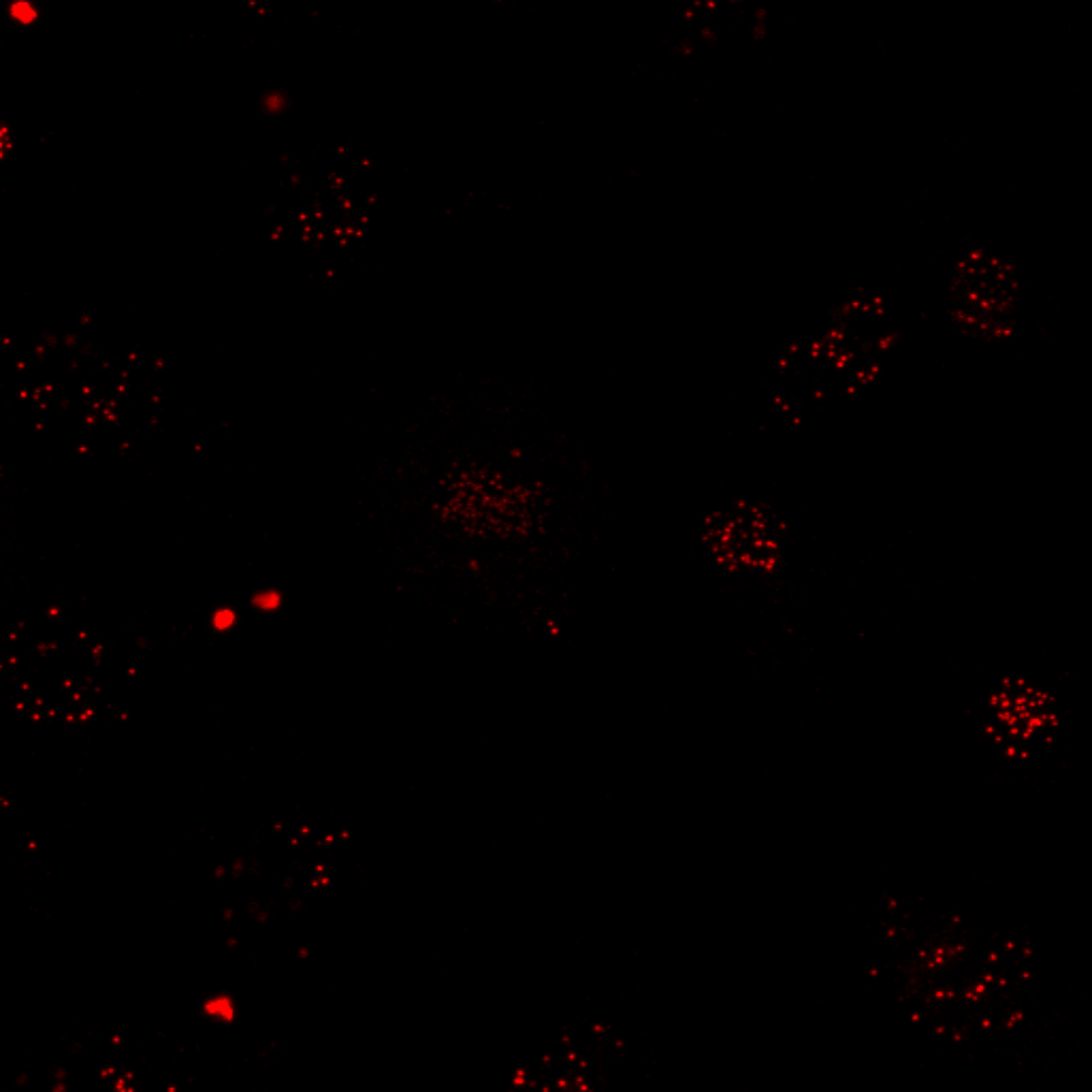

Supplement: Supplementary file 7 — Source Data Fig. 4 [file 44319_2024_106_MOESM7_ESM.zip › Figure 4/4J/+Doxycycline/CENPA.tif]

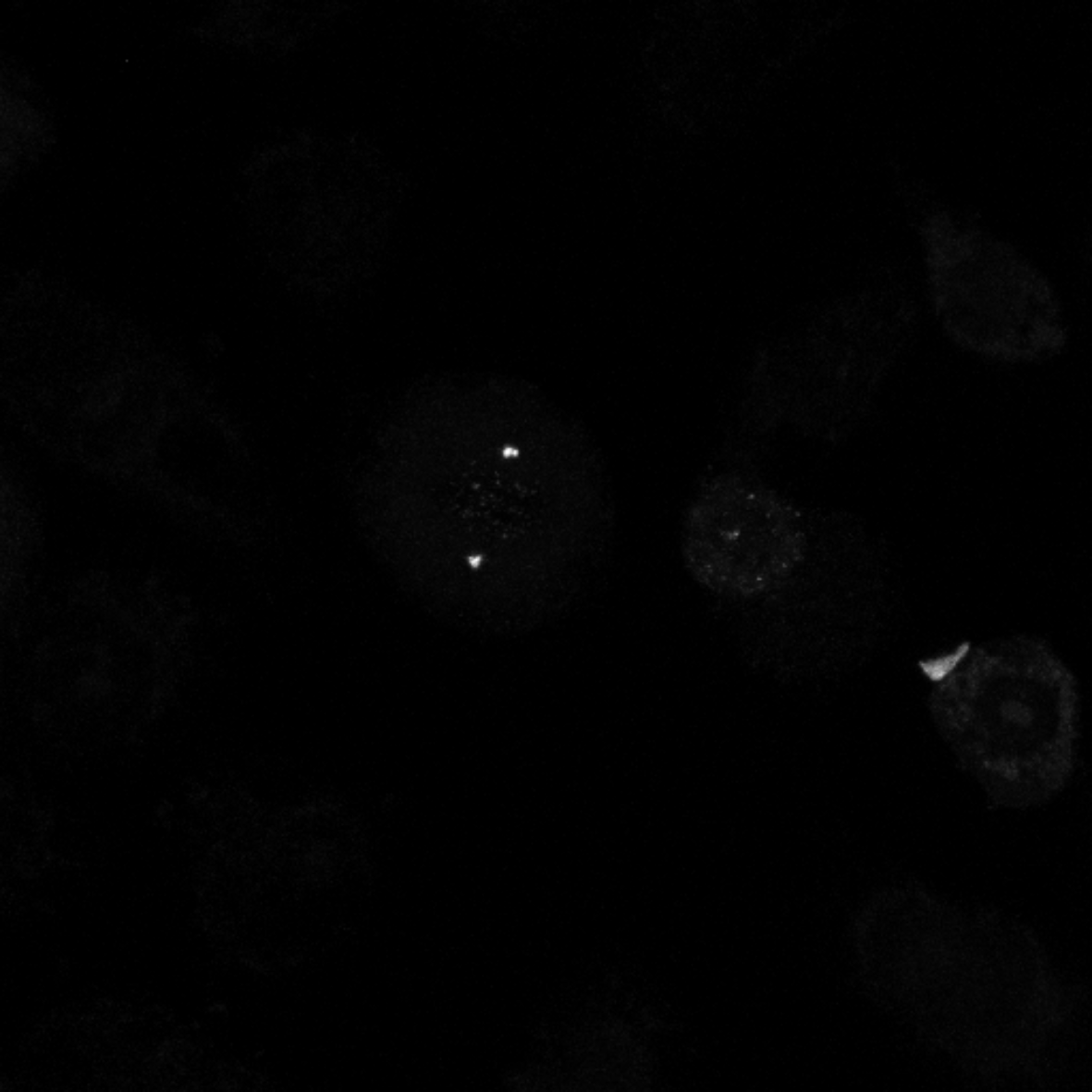

Supplement: Supplementary file 7 — Source Data Fig. 4 [file 44319_2024_106_MOESM7_ESM.zip › Figure 4/4J/+Doxycycline/GFP_GREY.tif]

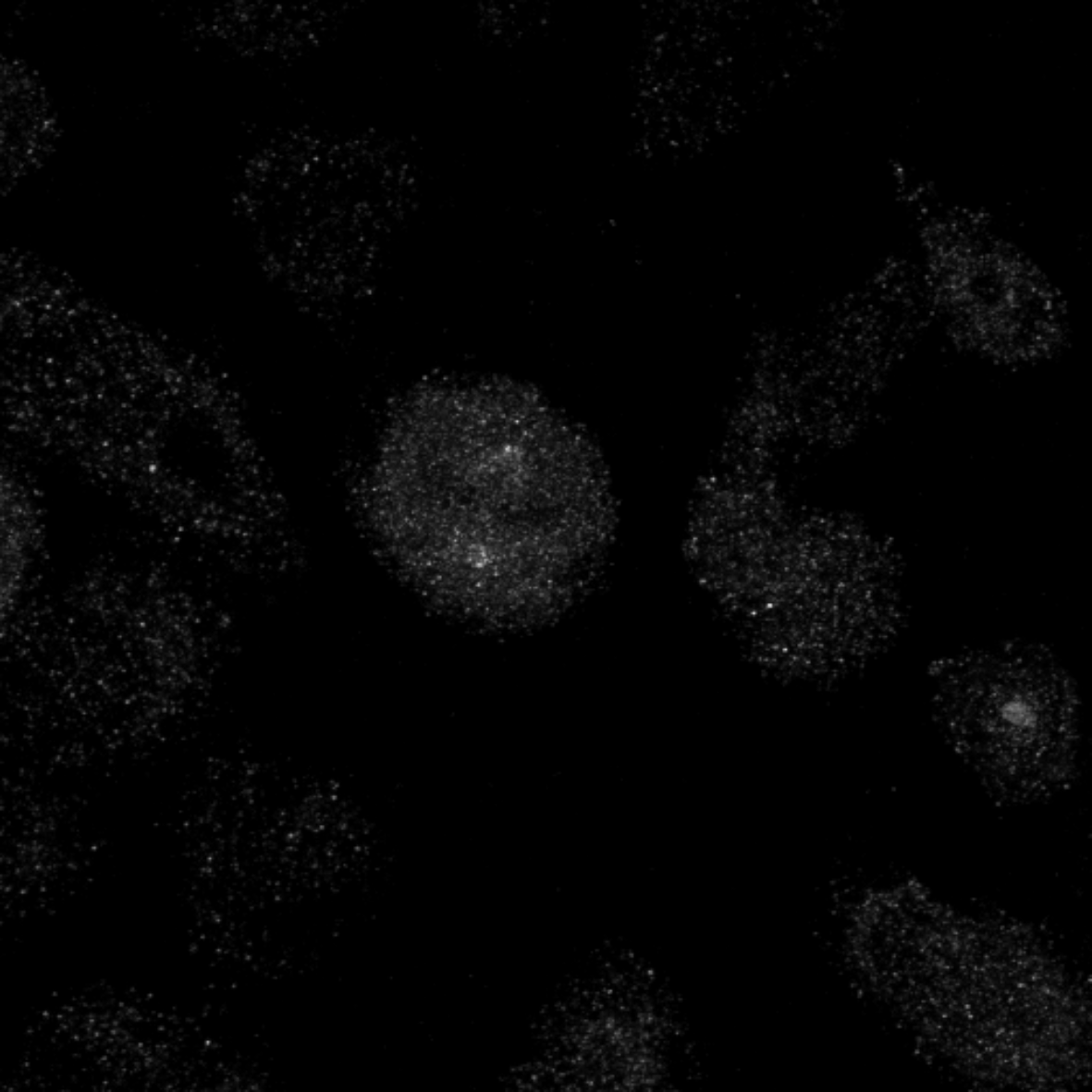

Supplement: Supplementary file 7 — Source Data Fig. 4 [file 44319_2024_106_MOESM7_ESM.zip › Figure 4/4J/+Doxycycline/TOG.tif]

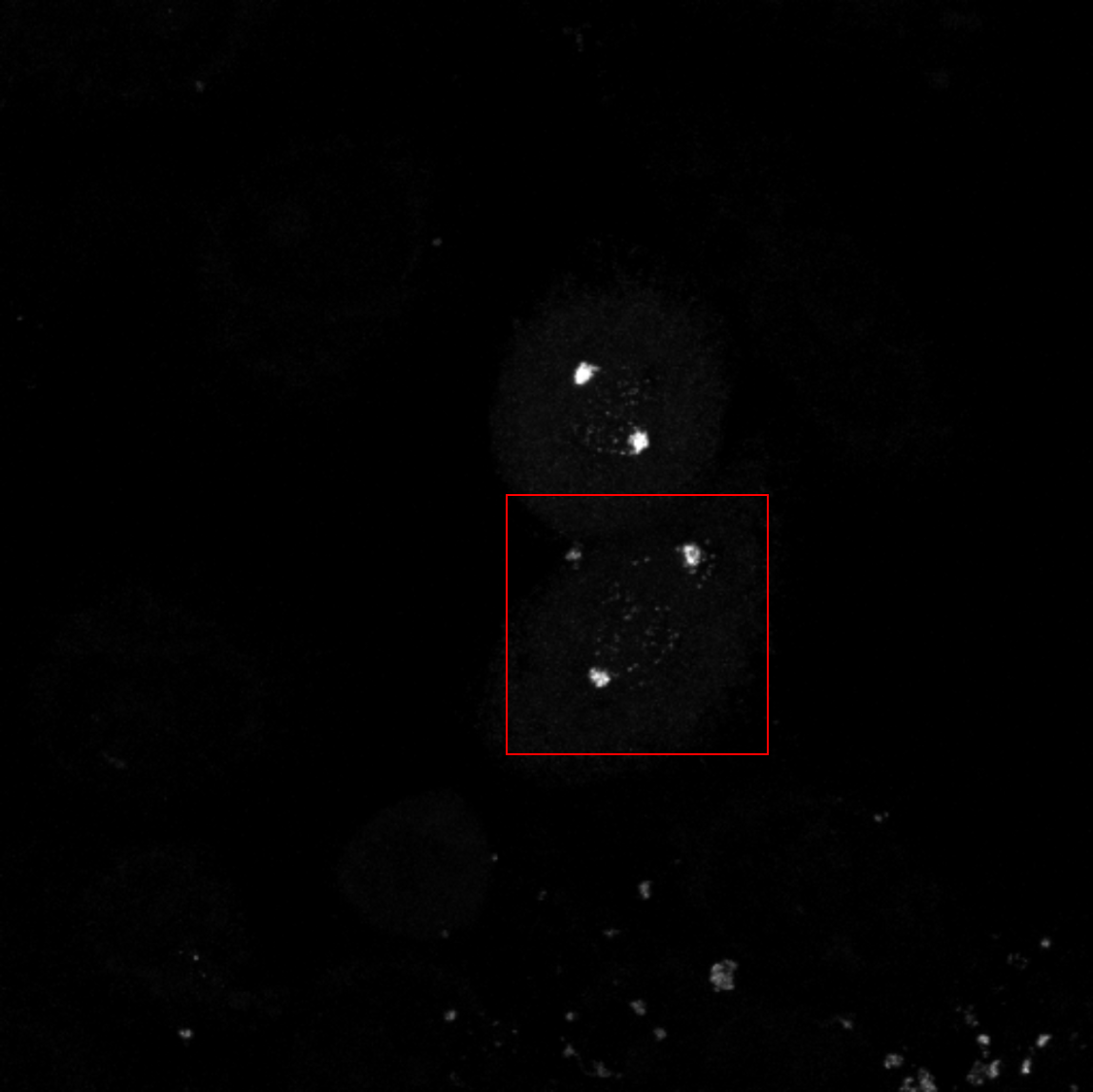

Supplement: Supplementary file 7 — Source Data Fig. 4 [file 44319_2024_106_MOESM7_ESM.zip › Figure 4/4J/-Doxycycline/Annotation.tif]

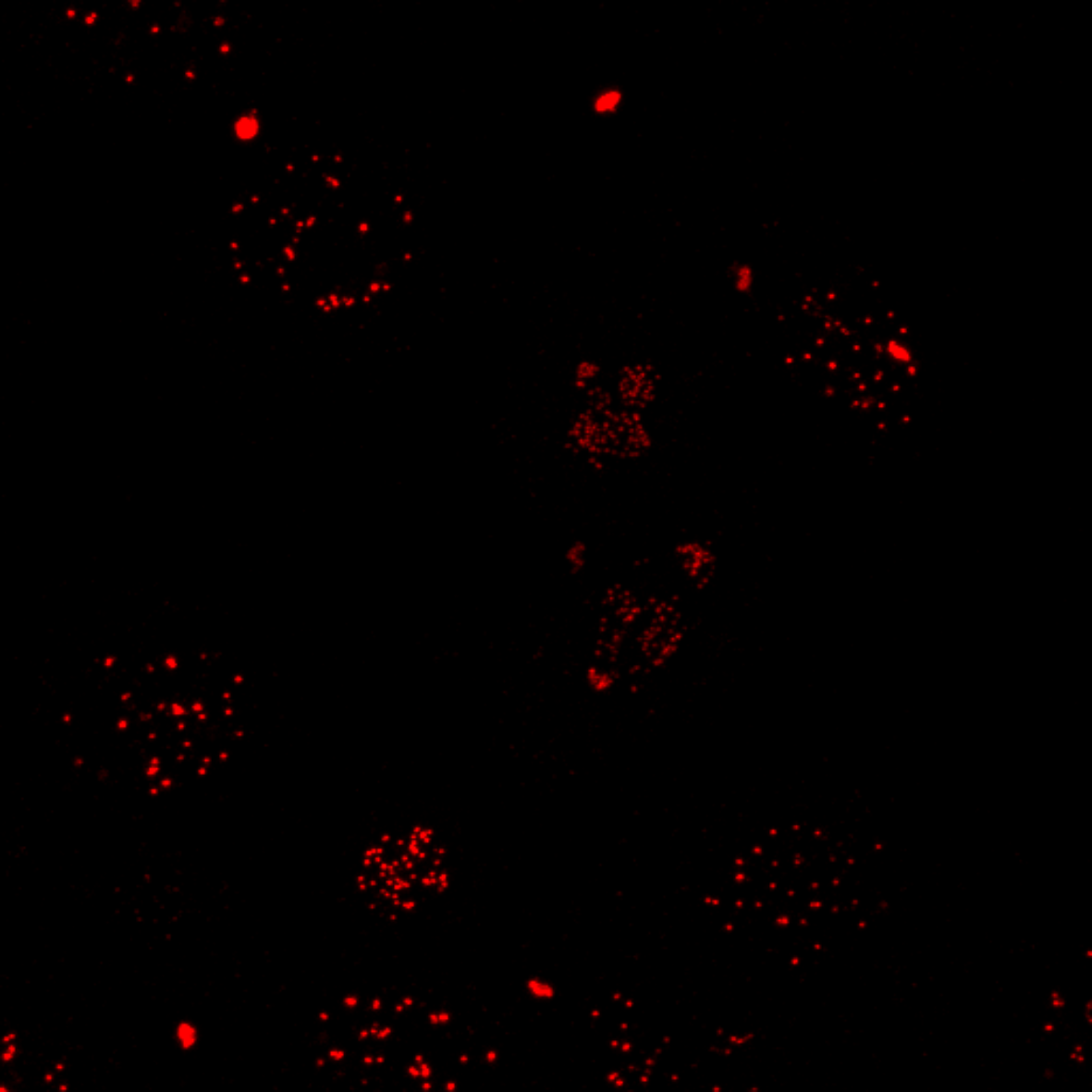

Supplement: Supplementary file 7 — Source Data Fig. 4 [file 44319_2024_106_MOESM7_ESM.zip › Figure 4/4J/-Doxycycline/CENPA.tif]

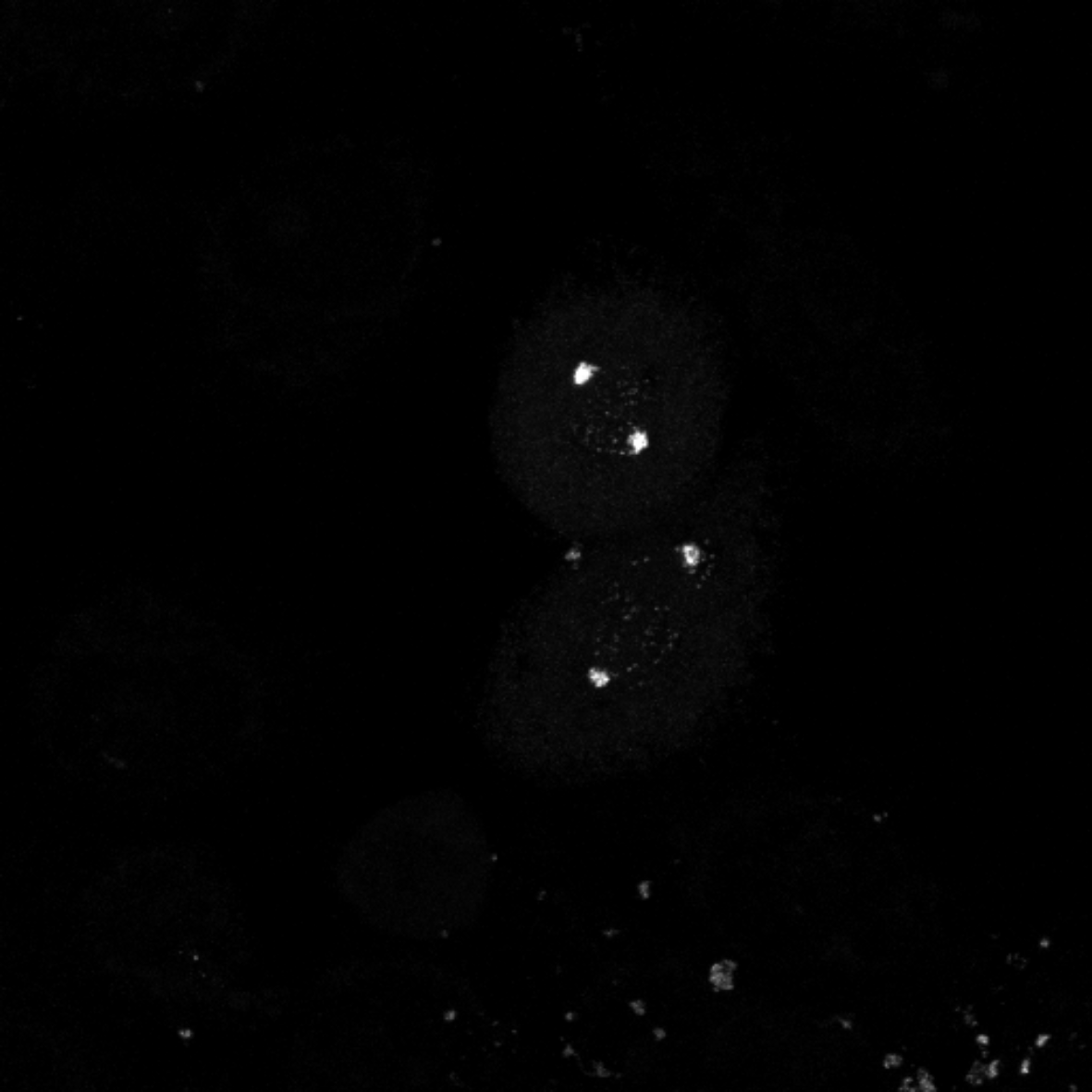

Supplement: Supplementary file 7 — Source Data Fig. 4 [file 44319_2024_106_MOESM7_ESM.zip › Figure 4/4J/-Doxycycline/GFP_GREY.tif]

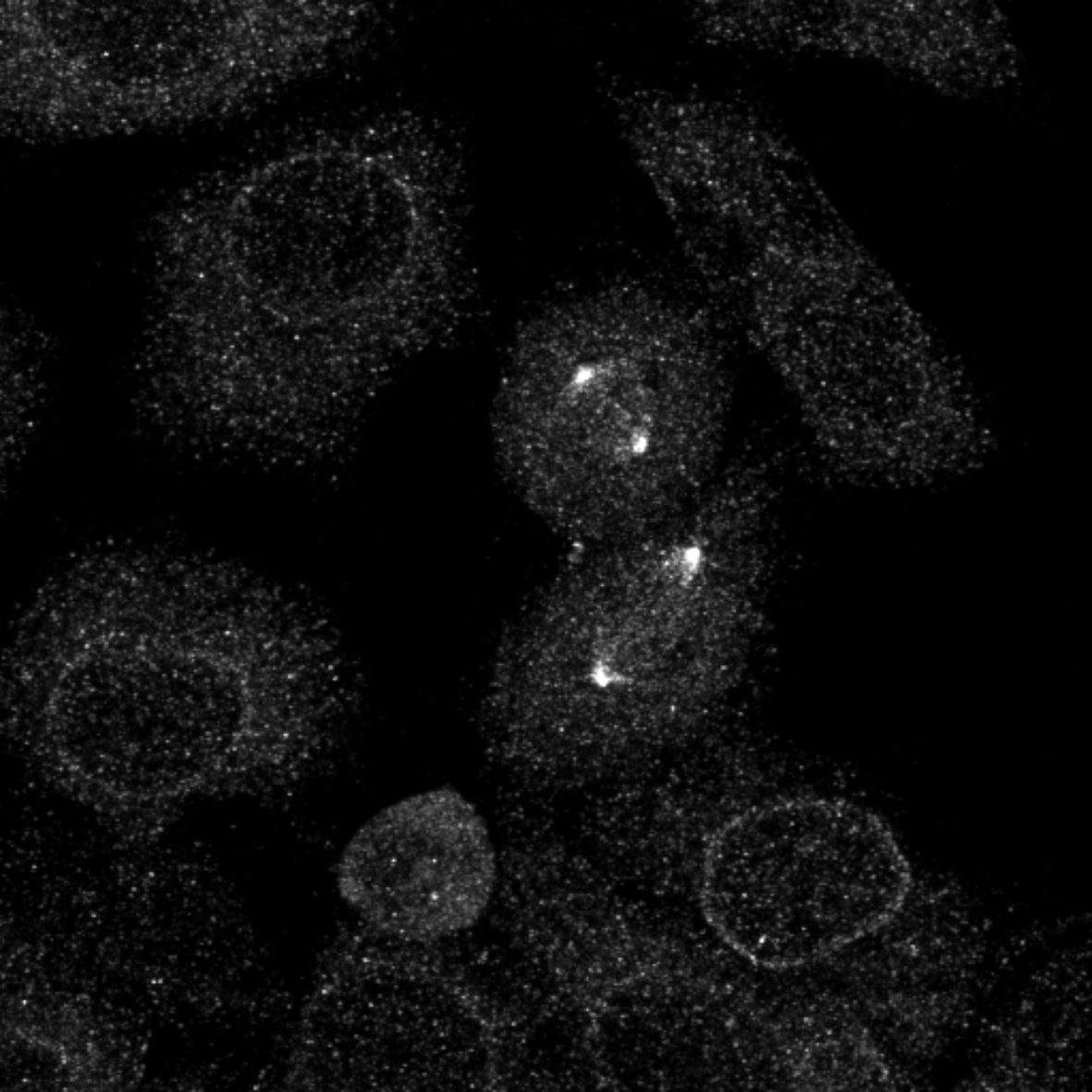

Supplement: Supplementary file 7 — Source Data Fig. 4 [file 44319_2024_106_MOESM7_ESM.zip › Figure 4/4J/-Doxycycline/TOG.tif]
