# Supplementary material for: CKAP5 stabilizes CENP-E at kinetochores by regulating microtubule-chromosome attachments
Source: EMBO Rep. 2024 Feb 29;25(4):1909–35. doi: 10.1038/s44319-024-00106-9 (PMC11014917; doi:10.1038/s44319-024-00106-9)
Supplement: Supplementary file 11 — Expanded View Figures [file 44319_2024_106_MOESM11_ESM.pdf]

Expanded View Figures

Figure EV1. CKAP5 depletion induces chromosome misalignment and removal of CENP-E from kinetochores.

(**A**) Representative immunofluorescence images showing level of CKAP5 depletion and chromosome misalignment phenotype. (**B**) Quantification of chromosome misalignment phenotypes ($n > 100$ cells in each case) in CKAP5-depleted cells compared to mock-treated (control esiRNA) cells from three independent experiments. Data represents mean $+/-$ SEM. (**C**, **D**) Western blots showing level of CKAP5 depletion and corresponding cellular levels of CENP-E. (**E**) Representative immunofluorescence images showing removal of CENP-E from partially aligned KTs of inducible Cas-9 expressing CKAP5 knockout HeLa cells (regions shown in box). (**F**, **G**) Immunofluorescence images showing loss of CENP-E localization at the partially aligned metaphase KTs (regions show in box) and prometaphase KTs of U2OS cells depleted of endogenous CKAP5. (**H**) Representative immunofluorescence images showing rescue of CENP-E localization at aligned KTs in CKAP5-Spindly co-depleted HeLa Kyoto cells compared to control metaphase and CKAP5-depleted cells (regions shown in box). (**I**) Dot plot showing mean intensity of endogenous CENP-E at aligned KTs (highlighted with boxes) normalized with that of ACA where each dot represents individual KT ($n = 400$–550) quantified from multiple cells from three independent experiments. ****$P < 0.0001$ by one-way ANOVA. Data represents mean $+/-$ SEM. (**J**) Western blot showing levels of CKAP5 and Spindly depletion in HeLa Kyoto cells. (**K**) Western blot showing endogenous CKAP5 depletion levels in the cells expressing GFP-tagged CKAP5-FL vs. different deletion constructs. (**L**) Immunofluorescence images showing chromosome phenotype in CKAP5-depleted cells (HeLa Kyoto) and in cells expressing fusion construct consisting of CKAP5$^{853\text{-}2032}$-GFP and CKAP5-shRNA. (**M**) Plot showing percentage of cells ($n = 73$ cells) with less than 10 polar chromosomes in CKAP5$^{853\text{-}2032}$-GFP-CKAP5-shRNA cells compared to CKAP5 esiRNA-treated cells (HeLa Kyoto). (**N**) Representative immunofluorescence image of HeLa Kyoto cell expressing CKAP5$^{1\text{-}1429}$-GFP in endogenous CKAP5-depleted background showing no recognizable KT or spindle localization. Scale bar of all images = 5 μm. Data Information: Chromosome congression defect and level of CKAP5 depletion (red) by immunofluorescence imaging (**A**). Rectangular boxes show aligned KTs with normal CENP-E localization in control vs. reduced CENP-E localization in CKAP5-depleted conditions (**E**, **F**). Doxycycline-inducible CKAP5 KO HeLa cells in (**E**) and U2OS cells in (**F**). CKAP5-depleted cells shows reduced CENP-E levels (green) at KTs compared to control cells when arrested in prometaphase by Eg5 kinesin inhibition (**G**). Rectangular boxes show region (aligned KTs) considered for CENP-E intensity analysis. Rescue in CENP-E levels (green) are observed upon co-depletion of spindly and CKAP5 (**H**). DAPI staining of chromosomes showing reduction in number of polar chromosomes upon expression of CKAP5$^{853\text{-}2032}$-GFP compared to that in CKAP5-depleted condition (**L**). CKAP5$^{1\text{-}1429}$-GFP (green) showing no localization in HeLa Kyoto cells when compared to CKAP5-FL-GFP (green) under endogenous CKAP5-depleted condition (**N**).

© The Author(s)

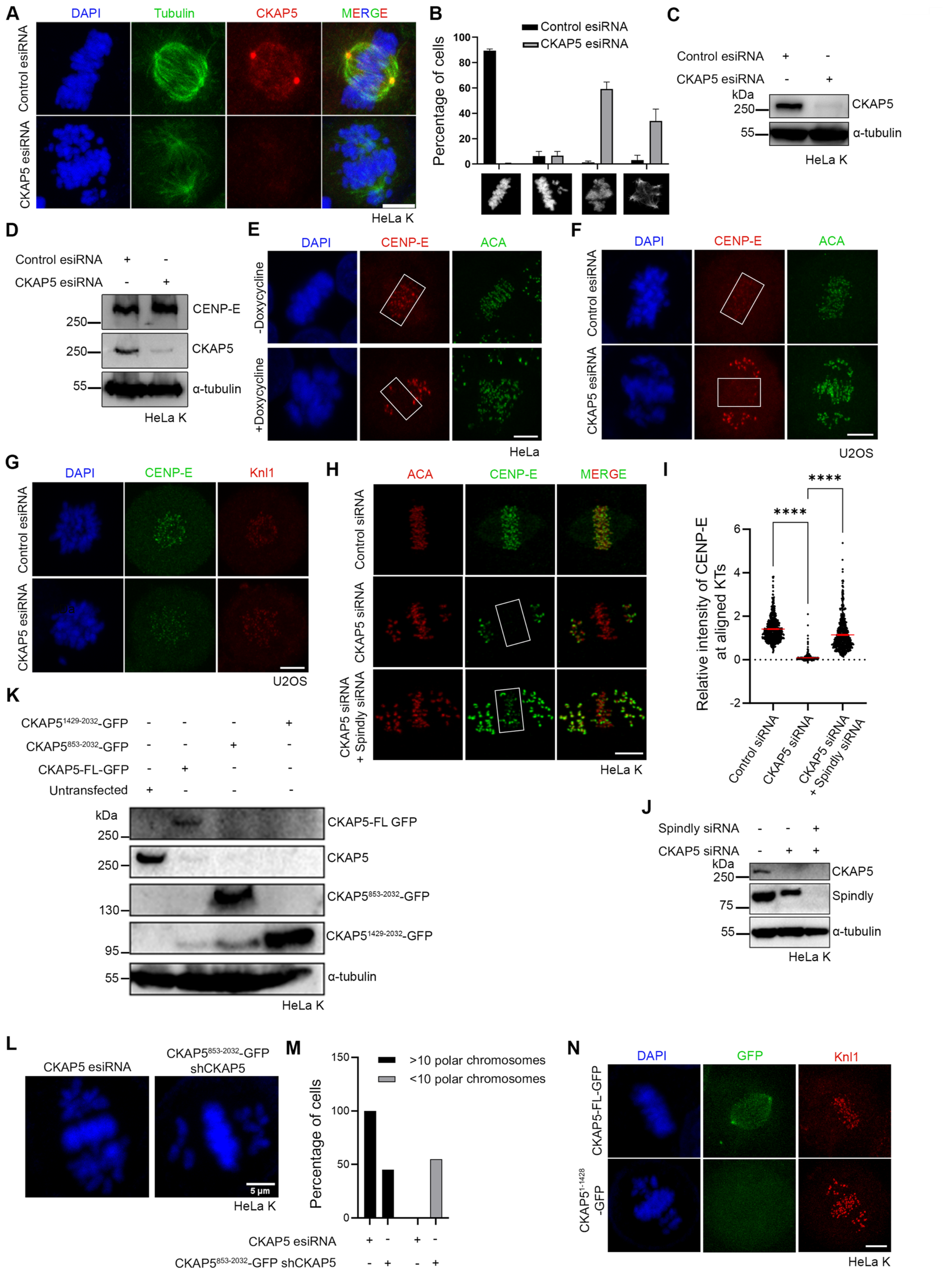

A
DAPI
Tubulin
CKAP5
MERGE
Control esiRNA
CKAP5 esiRNA
HeLa K
B
Control esiRNA
CKAP5 esiRNA
Percentage of cells
100
80
60
40
20
0
C
Control esiRNA + -
CKAP5 esiRNA - +
kDa
250
55
CKAP5
α-tubulin
HeLa K
D
Control esiRNA + -
CKAP5 esiRNA - +
250
250
55
CENP-E
CKAP5
α-tubulin
HeLa K
E
DAPI
CENP-E
ACA
-Doxycycline
+Doxycycline
HeLa
F
DAPI
CENP-E
ACA
Control esiRNA
CKAP5 esiRNA
U2OS
G
DAPI
CENP-E
Knl1
Control esiRNA
CKAP5 esiRNA
U2OS
H
ACA
CENP-E
MERGE
Control siRNA
CKAP5 siRNA
CKAP5 siRNA + Spindly siRNA
HeLa K
I
Relative intensity of CENP-E at aligned KTs
****
****
6
4
2
0
-2
Control siRNA
CKAP5 siRNA
CKAP5 siRNA + Spindly siRNA
J
Spindly siRNA - - +
CKAP5 siRNA - + +
kDa
250
75
55
CKAP5
Spindly
α-tubulin
HeLa K
K
CKAP5¹⁴²⁹⁻²⁰³²-GFP - - - +
CKAP5⁸⁵³⁻²⁰³²-GFP - - + -
CKAP5-FL-GFP - + - -
Untransfected + - - -
kDa
250
250
130
95
55
CKAP5-FL GFP
CKAP5
CKAP5⁸⁵³⁻²⁰³²-GFP
CKAP5¹⁴²⁹⁻²⁰³²-GFP
α-tubulin
HeLa K
L
CKAP5 esiRNA
CKAP5⁸⁵³⁻²⁰³²-GFP shCKAP5
5 µm
HeLa K
M
>10 polar chromosomes
<10 polar chromosomes
Percentage of cells
150
100
50
0
CKAP5 esiRNA + - + -
CKAP5⁸⁵³⁻²⁰³²-GFP shCKAP5 - + - +
N
DAPI
GFP
Knl1
CKAP5-FL-GFP
CKAP5¹⁻¹⁴²⁸ -GFP
HeLa K

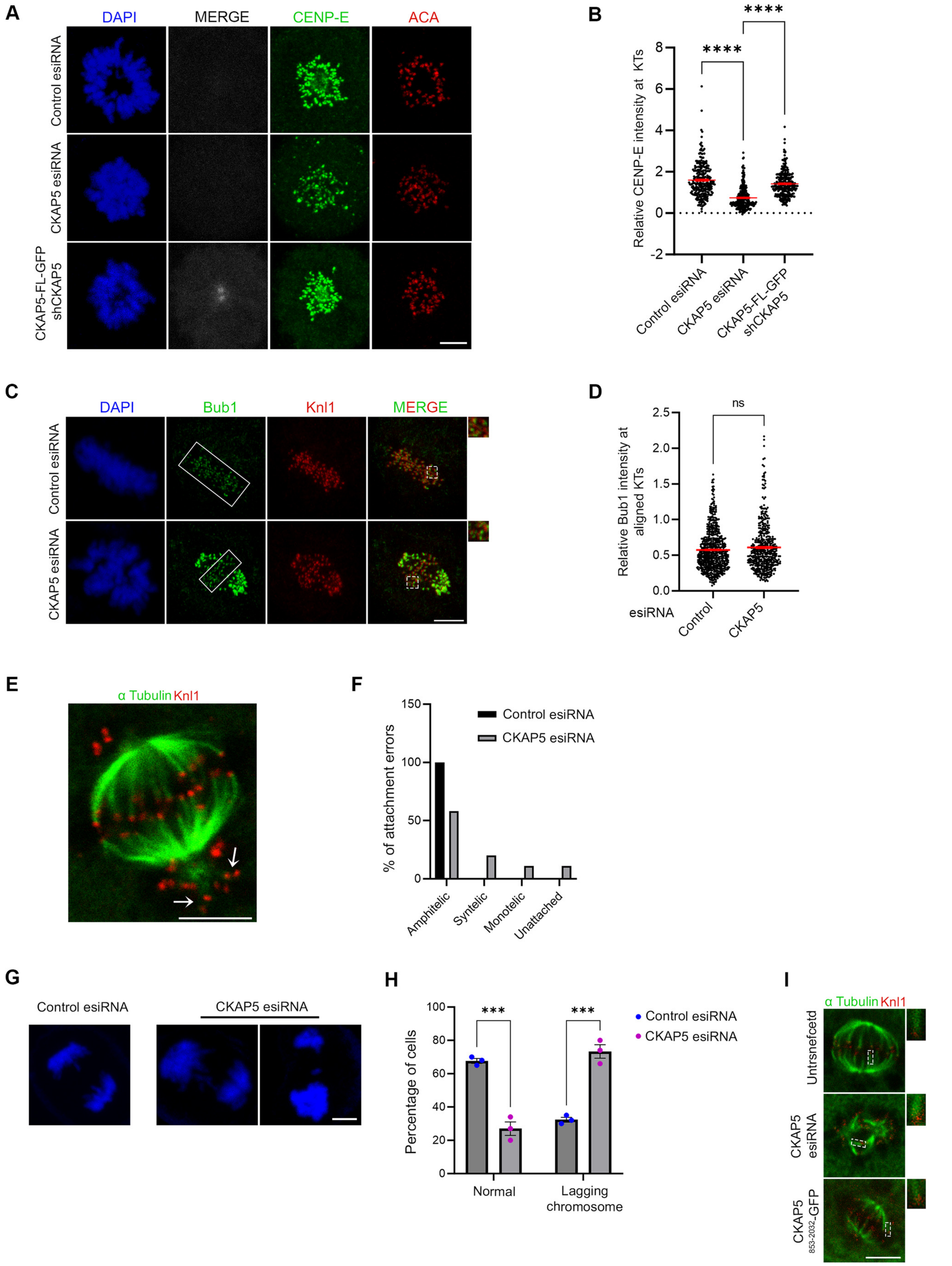
A
DAPI
MERGE
CENP-E
ACA
Control esiRNA
CKAP5 esiRNA
CKAP5-FL-GFP shCKAP5
B
****
****
Relative CENP-E intensity at KTs
Control esiRNA
CKAP5 esiRNA
CKAP5-FL-GFP shCKAP5
C
DAPI
Bub1
Knl1
MERGE
Control esiRNA
CKAP5 esiRNA
D
ns
Relative Bub1 intensity at aligned KTs
esiRNA
Control
CKAP5
E
α Tubulin Knl1
F
Control esiRNA
CKAP5 esiRNA
% of attachment errors
Amphitelic
Syntelic
Monotelic
Unattached
G
Control esiRNA
CKAP5 esiRNA
H
***
***
Control esiRNA
CKAP5 esiRNA
Percentage of cells
Normal
Lagging chromosome
I
α Tubulin Knl1
Untrsnefcetd
CKAP5 esiRNA
CKAP5 853-2032-GFP

 © The Author(s)

Figure EV2. CKAP5 depletion results in kinetochore-microtubule attachment errors and lagging chromosomes.

(**A**) Representative immunofluorescence images showing rescue of CENP-E (green) localization at prometaphase KTs of HeLa Kyoto cells expressing CKAP5-FL-GFP (grey) when compared to CKAP5-depleted cells. ACA (red) was used as KT marker. (**B**) Dot plot showing mean intensity of endogenous CENP-E at these KTs normalized with that of ACA where each dot represents individual KT (*n* ~270) quantified from multiple cells from three independent experiments. ****$P < 0.0001$ by one-way ANOVA. (**C**) Representative immunofluorescence image showing no significant change in localization of Bub1 at aligned KTs of control vs. CKAP5-depleted HeLa Kyoto cells. Insets show enlarged view of region marked by dotted boxes in the merge images. (**D**) Dot plots showing mean intensities of Bub1/Knl1 (*n* ~400 KTs) quantified from individual kinetochores of multiple cells from three independent experiments. (**E**) Representative immunofluorescence image showing syntelic attachments (marked with arrowheads) of polar chromosomes. (**F**) Plot showing percentage of amphitelic, syntelic, monotelic and unattached KTs (144 KT pairs from multiple cells) of pole-proximal chromosomes in CKAP5-depleted HeLa Kyoto cells. (**G**) Anaphase lagging chromosomes shown by CKAP5-depleted HeLa Kyoto cells. (**H**) Graph showing increased percentage of cells showing anaphase laggards upon depletion of CKAP5. More than 100 cells were analyzed from three independent experiments. ***$P = 0.0007$ by two-way ANOVA. (**I**) Representative immunofluorescence images (single plane) showing cold stable microtubule staining (α Tubulin in green; KNL1 in red) in HeLa Kyoto cells expressing CKAP5 esiRNA and CKAP5$^{853\text{-}2032}$-GFP compared to un-transfected HeLa Kyoto cells. Region in dashed boxes is enlarged in inset. Scale bar of all images = 5 µm. All data represents mean +/- SEM. Data Information: Expression of CKAP5-FL-GFP (grey) rescued CENP-E levels (green) at prometaphase KTs under endogenous CKAP5-depleted condition (**A**). Rectangular boxes show region (aligned KTs) taken for Bub1 intensity analysis. Dotted boxes in merged images showing few KTs enlarged in inset. Bub1 (green) levels showed no difference (**C**). Arrows show KTs of polar chromosomes with syntelic attachments (**E**). DAPI staining of anaphase chromosomes of control and CKAP5-depleted cells. CKAP5-depleted cells showed multiple lagging chromosomes (**G**). Dotted rectangular boxes are enlarged in insets showing increased MT (green) thickness in CKAP5-depleted condition and rescued MT intensity upon expression of CKAP5$^{853\text{-}2032}$-GFP (**I**).

© The Author(s)

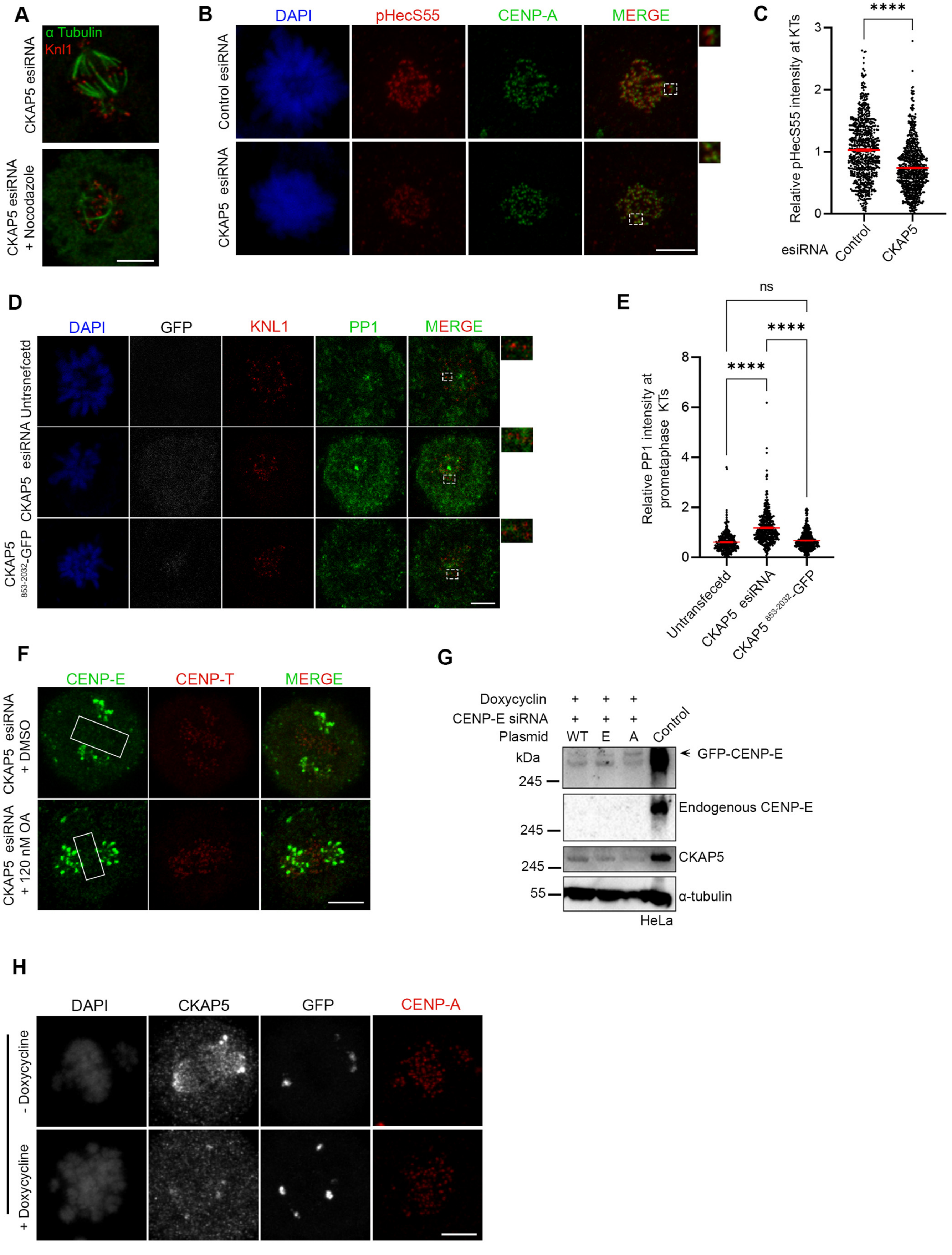
A
α Tubulin
Knl1
CKAP5 esiRNA
CKAP5 esiRNA + Nocodazole
B
DAPI
pHecS55
CENP-A
MERGE
Control esiRNA
CKAP5 esiRNA
C
****
Relative pHecS55 intensity at KTs
3
2
1
0
esiRNA
Control
CKAP5
D
DAPI
GFP
KNL1
PP1
MERGE
Untrsnefcetd
CKAP5 esiRNA
CKAP5 853-2032-GFP
E
ns
****
****
Relative PP1 intensity at prometaphase KTs
8
6
4
2
0
Untransfecetd
CKAP5 esiRNA
CKAP5 853-2032-GFP
F
CENP-E
CENP-T
MERGE
CKAP5 esiRNA + DMSO
CKAP5 esiRNA + 120 nM OA
G
Doxycyclin + + +
CENP-E siRNA + + +
Plasmid WT E A Control
kDa
GFP-CENP-E
245
Endogenous CENP-E
245
CKAP5
245
55
α-tubulin
HeLa
H
DAPI
CKAP5
GFP
CENP-A
GFP-CENP-E-T422A + CENP-E siRNA
- Doxycycline
+ Doxycycline

Figure EV3. CKAP5 depletion leads to increased PP1 activity at the partially aligned kinetochores.

(**A**) Representative immunofluorescence image showing partial destabilization of MTs in CKAP5-depleted HeLa Kyoto cells using 3.3 μM nocodazole for 10 min. (α Tubulin in green; KNL1 in red). (**B**) Immunofluorescence images showing pHecS55 (red) and CENP-A (green) at prometaphase kinetochores of control and CKAP5-depleted HeLa Kyoto cells. Insets show the enlarged view of the dashed boxes. (**C**) Dot plot showing mean fluorescence intensity of pHecS55 normalized to that of CENP-A quantified from individual kinetochores (*n* ~700 KTs) of multiple cells from three independent experiments. ****$P < 0.0001$ by Student's *t* test. (**D**) Immunofluorescence images (single plane) showing PP1 (green) and KNL1 (red) at prometaphase KTs of HeLa Kyoto cells expressing CKAP5 esiRNA and $CKAP5^{853\text{-}2032}$-GFP compared to un-transfected cells. Regions in dashed boxes are enlarged in insets. (**E**) Dot plot showing mean fluorescence intensity of PP1 from individual kinetochores (*n* ~400 KTs) normalized with that of KNL1. KTs from individual z planes of multiple cells were analyzed from three independent experiments. ****$P < 0.0001$ by one-way ANOVA. (**F**) Representative immunofluorescence image showing localization of CENP-E in CKAP5 esiRNA-treated cells incubated with DMSO or 120 nM okadaic acid. Boxes show region of aligned KTs. (**G**) Western blot showing endogenous CENP-E depletion and doxycycline-induced depletion of CKAP5 in doxycycline-inducible CKAP5 KO HeLa. (WT-Wildtype; A-T422A; E- T422E). (**H**) Representative immunofluorescence images showing KT localization of GFP-CENP-E-T422A in the presence or absence of doxycycline in doxycycline-inducible CKAP5 KO HeLa cells under endogenous CENP-E depleted background. All data represent mean $+/-$ SEM. Scale bar of all images = 5 μm. Data Information: MTs (green) show partial destabilization upon treatment with 3.3 μM nocodazole for 10 min (**A**). Dotted boxes in merged image are enlarged in insets showing increased levels of pHecS55 (red) at prometaphase KTs upon CKAP5 depletion (**B**). Dotted boxes in merged image show few KTs that are enlarged in insets. PP1 levels (green) at prometaphase KTs were rescued upon expression of $CKAP5^{853\text{-}2032}$-GFP (**D**). Rectangular boxes show level of CENP-E (green) at aligned KTs. No change in CENP-E levels were observed upon treatment with low concentration of okadaic acid to inhibit PP2A (**F**). GFP-CENP-E-T422A showed no localization to aligned KTs in the presence or absence of CKAP5 (**H**).

© The Author(s)

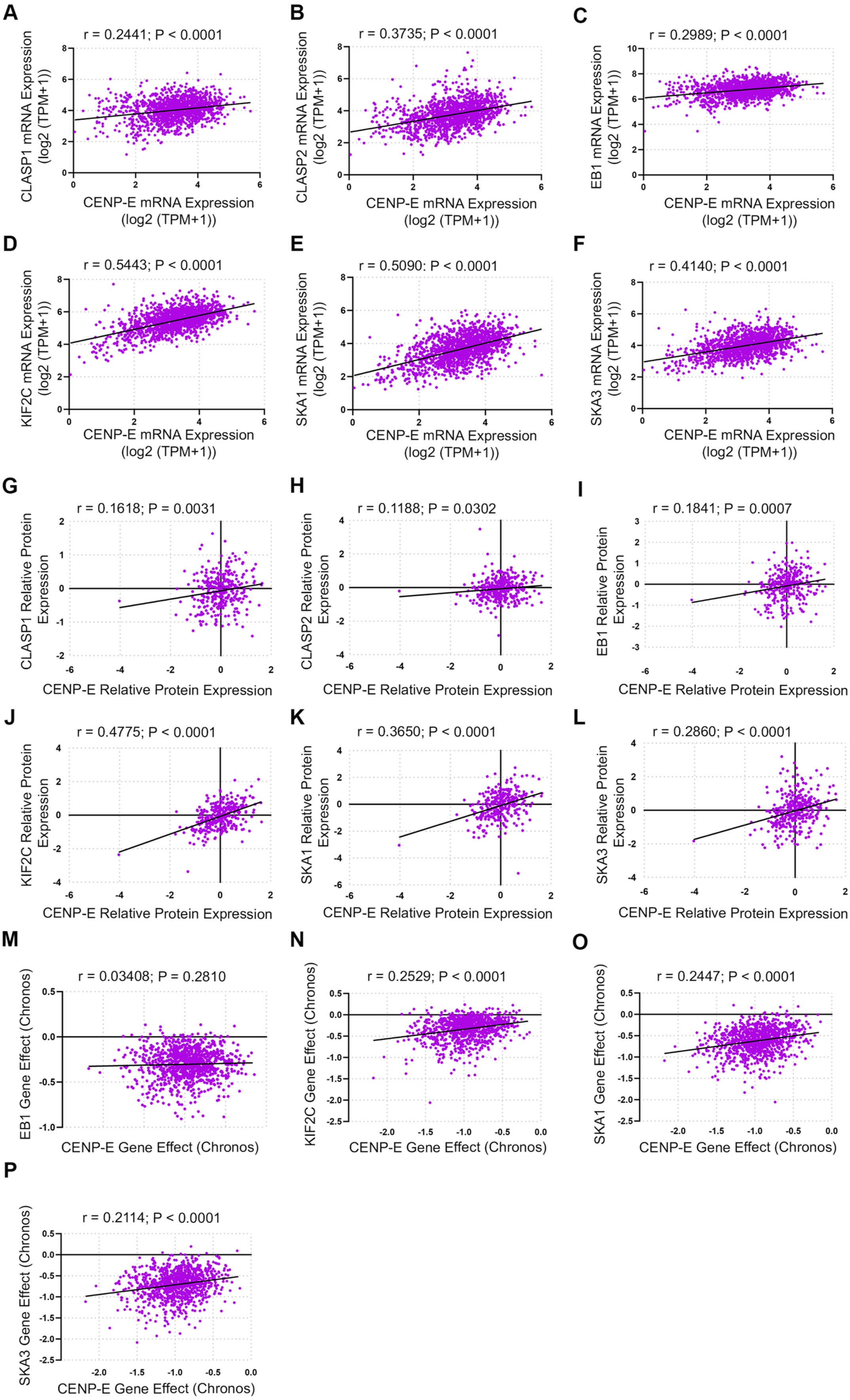
A
r = 0.2441; P < 0.0001
CLASP1 mRNA Expression (log2 (TPM+1))
CENP-E mRNA Expression (log2 (TPM+1))
B
r = 0.3735; P < 0.0001
CLASP2 mRNA Expression (log2 (TPM+1))
CENP-E mRNA Expression (log2 (TPM+1))
C
r = 0.2989; P < 0.0001
EB1 mRNA Expression (log2 (TPM+1))
CENP-E mRNA Expression (log2 (TPM+1))
D
r = 0.5443; P < 0.0001
KIF2C mRNA Expression (log2 (TPM+1))
CENP-E mRNA Expression (log2 (TPM+1))
E
r = 0.5090: P < 0.0001
SKA1 mRNA Expression (log2 (TPM+1))
CENP-E mRNA Expression (log2 (TPM+1))
F
r = 0.4140; P < 0.0001
SKA3 mRNA Expression (log2 (TPM+1))
CENP-E mRNA Expression (log2 (TPM+1))
G
r = 0.1618; P = 0.0031
CLASP1 Relative Protein Expression
CENP-E Relative Protein Expression
H
r = 0.1188; P = 0.0302
CLASP2 Relative Protein Expression
CENP-E Relative Protein Expression
I
r = 0.1841; P = 0.0007
EB1 Relative Protein Expression
CENP-E Relative Protein Expression
J
r = 0.4775; P < 0.0001
KIF2C Relative Protein Expression
CENP-E Relative Protein Expression
K
r = 0.3650; P < 0.0001
SKA1 Relative Protein Expression
CENP-E Relative Protein Expression
L
r = 0.2860; P < 0.0001
SKA3 Relative Protein Expression
CENP-E Relative Protein Expression
M
r = 0.03408; P = 0.2810
EB1 Gene Effect (Chronos)
CENP-E Gene Effect (Chronos)
N
r = 0.2529; P < 0.0001
KIF2C Gene Effect (Chronos)
CENP-E Gene Effect (Chronos)
O
r = 0.2447; P < 0.0001
SKA1 Gene Effect (Chronos)
CENP-E Gene Effect (Chronos)
P
r = 0.2114; P < 0.0001
SKA3 Gene Effect (Chronos)
CENP-E Gene Effect (Chronos)

 © The Author(s)

Figure EV4. Correlation between CKAP5 and CENP-E with respect to other spindle/kinetochore proteins.

(**A–F**) Correlation of mRNA expression of CENP-E and multiple spindle/kinetochore proteins. (**A**) CLASP1, (**B**) CLASP2, (**C**) EB1, (**D**) KIF2C, (**E**) SKA1, (**F**) SKA3. The correlation is similar across proteins and similar to the correlation between CKAP5 and CENP-E mRNA expression (Fig. 6E). (**G–L**) Correlation of protein expression of CENP-E and multiple spindle/kinetochore proteins—(**G**) CLASP1, (**H**) CLASP2, (**I**) EB1, (**J**) KIF2C, (**K**) SKA1, (**L**) SKA3. The correlation is similar across proteins and similar to the correlation between CKAP5 and CENP-E protein expression (Fig. 6F). (**M–P**) Correlation of sensitivity to CRISPR gene perturbation of CENP-E and multiple spindle/kinetochore proteins. All data is normalized to the mRNA expression of the proliferation marker MKI67 as a covariate using a linear model. (**M**) EB1, (**N**) KIF2C, (**O**) SKA1, (**P**) SKA3. The correlation is similar across proteins and to the correlation between CKAP5 and CENP-E CRISPR perturbation sensitivity (Fig. 6G). All data is normalized to the mRNA expression of the proliferation marker MKI67 as a covariate using a linear model. Two-sided Pearson's correlation was used for all the data analysis.

© The Author(s)
